# Distinct Non-conservative Behavior of Dissolved Organic Matter after Mixing Solimões/Negro and Amazon/Tapajós River Waters

**DOI:** 10.1021/acsestwater.2c00621

**Published:** 2023-06-12

**Authors:** Siyu Li, Mourad Harir, Philippe Schmitt-Kopplin, Fausto Machado-Silva, Michael Gonsior, David Bastviken, Alex Enrich-Prast, Juliana Valle, Norbert Hertkorn

**Affiliations:** † Research Unit Analytical Biogeochemistry, Helmholtz Munich, Ingolstaedter Landstrasse 1, Neuherberg 85764, Germany; ‡ Chair of Analytical Food Chemistry, Technische Universität München, Alte Akademie 10, Freising-Weihenstephan 85354, Germany; § Program in Geosciences—Environmental Geochemistry, Chemistry Institute, Fluminense Federal University, Niteroi 24020-141, Brazil; ∥ Department of Environmental Sciences, University of Toledo, Toledo, Ohio 43606, United States; ⊥ Chesapeake Biological Laboratory, University of Maryland Center for Environmental Science, Solomons, Maryland 20688, United States; # Department of Thematic Studies—Environmental Change and Biogas Solutions Research Center (BSRC), Linköping University, Linköping SE-581 83, Sweden; ∇ Multiuser Unit of Environmental Analysis, University Federal of Rio de Janeiro, Rio de Janeiro 11070-100, Brazil; ○ Department of Thematic Studies—Environmental Change, Linköping University, Linköping SE-581 83, Sweden

**Keywords:** FT-ICR MS, NMR, mixing zones, transport, transformation, decomposition, biogeochemical cycling

## Abstract

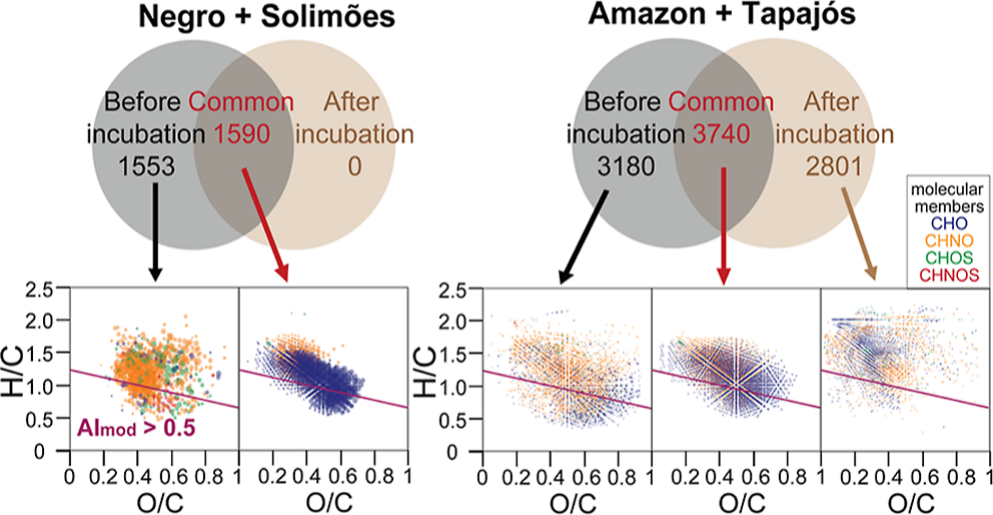

Positive and negative electrospray ionization Fourier transform ion cyclotron resonance mass spectrometry and ^1^H NMR revealed major compositional and structural changes of dissolved organic matter (DOM) after mixing two sets of river waters in Amazon confluences: the Solimões and Negro Rivers (S + N) and the Amazon and Tapajós Rivers (A + T). We also studied the effects of water mixing ratios and incubation time on the composition and structure of DOM molecules. NMR spectra demonstrated large-scale structural transformations in the case of S + N mixing, with gain of pure and functionalized aliphatic units and loss of all other structures after 1d incubation. A + T mixing resulted in comparatively minor structural alterations, with a major gain of small aliphatic biomolecular binding motifs. Remarkably, structural alterations from mixing to 1d incubation were in essence reversed from 1d to 5d incubation for both S + N and A + T mixing experiments. Heterotrophic bacterial production (HBP) in endmembers S, N, and S + N mixtures remained near 0.03 μgC L^–1^ h^–1^, whereas HBP in A, T, and A + T were about five times higher. High rates of dark carbon fixation took place at S + N mixing in particular. In-depth biogeochemical characterization revealed major distinctions between DOM biogeochemical changes and temporal evolution at these key confluence sites within the Amazon basin.

## Abstract 2

Minimal research studied DOM processing in the world’s largest river confluences at a molecular level. This study reports extensive structural changes of DOM molecules after mixing Amazon River waters with distinct characteristics and conducting short-term culture, contrary to the common viewpoint that Amazon DOM is largely inert.

## Introduction

The Amazon basin is the largest tropical drainage basin in the world, covering an area of approximately 7,000,000 km^2^.^1^ The Amazon River transports ∼36.1 Tg C yr^–1^ of organic carbon (of which 60–70% is dissolved) to the Atlantic Ocean.^2^ The Amazon River originates in the Andes cordillera, from where it carries large amounts of sediment. Along its flow path, it connects with several major tributaries of distinct chemical characteristics: “Blackwaters” that are relatively acidic (pH ∼ 5), low in total cations, and rich in dissolved organic matter (DOM), such as the Negro River; “Whitewaters” that show a near-neutral pH and are relatively rich in total cations and suspended sediments, such as the Solimões and Madeira River, and “Clearwaters” that are characterized by low suspended sediment loads, and high light transparency, such as the Tapajós River.^3,4^ DOM cycling varies greatly in those chemical environments.^5^ “Clearwaters” offer greater light transparency than the other types of Amazon waters and showed lower concentration of chromophoric dissolved organic matter (CDOM), the most readily photodegraded component of DOM molecules. In contrast, CDOM is the most abundant DOM fraction in “Blackwaters”. In a previous study, sunlight exposure over 27 h showed that at least 15% of Rio Negro DOM was photoreactive.^6^ The suspended sediments and cations in “Whitewaters” contribute to shielding from sunlight,^7^ and transform DOM through mineral particle adsorption and complexation,^8^ thereby affecting bioavailability as well.^9^ The confluences of major Amazon basin rivers can be regarded as effluents of entire aquatic ecosystems, and mixing causes abrupt changes in environmental conditions including the temperature, density, flow characteristics, pH, concentration of ions and (mineral and organic) particles, DOM composition, and molecular structure.^10,11^ Steep gradients develop for all these features in mixing zones, with distinct spatial and temporal evolution of interdependent variables. Opportunities arise for many non-conservative effects upon riverine mixing, making those places hot spots for organic matter processing and microbial metabolism influencing the dynamics of carbon pools in natural systems.^12,13^

DOM is a highly complex mixture of thousands to millions of individual molecules and is ubiquitous in terrestrial and aquatic ecosystems.^5,10,14−16^ DOM is an integral component of food webs, acting as both metabolic waste and substrate to heterotrophs, involving critical ecosystem processes such as nutrient availability and growth efficiency of aquatic organisms, and ultimately the cycling of carbon and other elements.^17,18^ Several abiotic processes, such as photochemistry, redox chemistry, mineral sorption, and desorption, affect the composition and structure of DOM molecules.^8,19^ Photochemical reactions in oxic environments contribute to oxygenation of DOM molecules, mainly by introduction of hydroxyl and carboxy functional groups;^20−22^ heteroatoms N and S in DOM molecules introduce alternative selectivity in photoprocessing of DOM molecules.^23^ Photoproduction of small oxygenated molecules, including photomineralization to CO_2_, that are or resemble biochemical metabolites^22^ will enhance microbial activity.^24^ Coupled photochemical and microbial DOM processing is context-dependent, structure-selective, and subject to synergy and competition.^25^

Most studies report distinct non-conservative behavior of DOM after riverine mixing affected by factors like phase partitioning,^14,26^ respiration,^27,28^ trace metal complexation,^10^ and discharge.^29^ However, the effects of physical mixing of different Amazon basin riverine waters on the composition and structure of its DOM at molecular resolution remain ill-constrained. We used ultrahigh-resolution negative and positive mode electrospray ionization (ESI[±]) Fourier transform ion cyclotron resonance mass spectrometry (FT-ICR MS), as well as ^1^H nuclear magnetic resonance spectroscopy (NMR) to study the composition and reactivity of DOM. We investigated the mixing of water samples from two major Amazon mixing zones, i.e., Solimões and Negro Rivers (S + N), and Amazon and Tapajós rivers (A + T). We conducted controlled mixing and incubation experiments with different mixing ratios and times of incubation after mixing, followed by biogeochemical and molecular characterization of the original and cultured samples to study the temporal evolution of DOM molecular composition and structure. We also measured the heterotrophic bacterial production (HBP) and dark carbon fixation (DCF) to assess microbial activities before and after the water mixing. We investigated how DOM molecular features change following mixing of the endmember river waters with different biogeochemical characteristics and investigated the factors that lead to the changes.

## Materials and Methods

### Sample Collection

**Figure 1:**
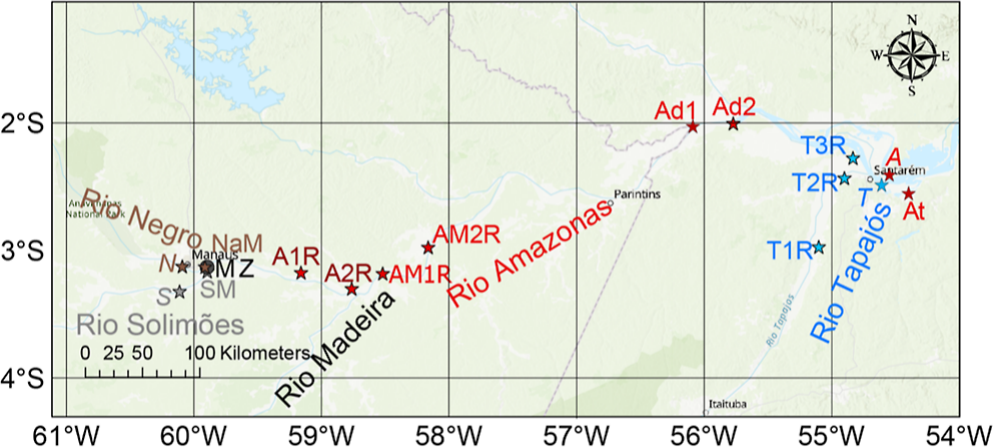
Surface water sampling locations. Samples Solimões (*S*) and Negro (*N*), Amazon River (*A*), and Tapajós River (*T*) refer to endmembers used for laboratory mixing and incubation experiments; all the other samples belong to the regional sample set.

We collected water samples at 17 sites in the Amazon basin eastward between April 2nd and May 25th in 2014 during an exceptional high-water period (Figure 1, Table S1). We obtained water samples by boat just below the surface and collected 10 L of each sample. Solid-phase extraction (SPE) was carried out in the field immediately after sampling using 1 g cartridges of PPL resin.^30^ The SPE-extracted DOM (SPE-DOM) in methanol was stored in the freezer (−20 °C) until FT-ICR MS and ^1^H NMR analyses. For more sampling and experimental details, see Supporting Information.

### Sample Processing

The four endmember samples *S*, *N*, *A*, and *T* were used for laboratory mixing and incubation experiments (Table S2). Mixing and incubation experiments with unfiltered endmembers were conducted on the bank or ship directly after sampling by mixing variable ratios of different waters (80:20, 60:40, 50:50, 40:60, and 20:80, total volume 500 mL) in Pyrex bottles, followed by one day (1d) and five days (5d) dark incubation at in situ temperature (30 °C) and SPE in the field. We also made a mixture of identical volumes of *S* and *N* and let it stand in dark for 30 min, named S50N50_30min.

Moreover, the endmember samples *S* and *N* were separated into “filtered water” and “filtered suspended solids” based on the treatment of the endmember samples before mixing (Tables S1 and S2). The waters and suspended solids were isolated by filtration of identical volumes of water from each river using 0.2 μM Whatman GF/F glass fiber filter (precombusted at 450 °C). The mixture of filtered Negro water and filtered Solimões suspended solids was named FNSS; the mixture of filtered Solimões water and filtered Negro suspended solids was named FSSN; the mixture of filtered Solimões water and filtered Negro water was named FSFN. FNSS and FSSN were left to sit in the dark for 1 day at in situ temperature (30 °C) and/or on ice (0 °C).

### Water Analysis

Water parameters were measured on part of the samples (Table S5). Water conductivity, temperature, and dissolved oxygen were measured in situ with a portable instrument (Hanna Instruments, Metrohm electrode, and PRO-ODO YSI). Dissolved and particulate organic carbon, inorganic nutrients, and pH were determined by standard procedures, as described in Supporting Information.

### FT-ICR MS Analysis

ESI[±] FT-ICR mass spectra of SPE-DOM were acquired using a 12 T Bruker Solarix mass spectrometer (Bruker Daltonics, Bremen, Germany) and an Apollo II ESI source.^15^ Data processing used Compass Data Analysis 5.0 (Bruker, Bremen, Germany) and formula assignment used an in-house software (NetCalc);^31^ and further details are described in Supporting Information.

### ^1^H NMR Analysis

800 MHz ^1^H NMR spectra of SPE-DOM were acquired with a Bruker ADVANCE III NMR spectrometer operating at 800.35 MHz (*B*_0_ = 18.8 tesla) at 283 K from redissolved dried SPE-DOM in CD_3_OD, as described in the Supporting Information. ^1^H NMR spectra enabled quantification of key structural units in SPE-DOM, including pure and oxygenated aliphatics, carboxyl-rich alicyclic molecules,^20^ olefins, and aromatics.

### Microbial Analysis

Microbial activity was measured in part of the samples (Table S10). Heterotrophic bacterial production (HBP) refers to the assimilatory metabolism consuming preferentially labile DOM while refractory DOM is mostly respired^32^ and was determined by the measurement of protein incorporation of radio-labeled ^3^H-Leucine. Dark carbon fixation (DCF) is inorganic light-independent C-uptake, mostly performed by chemosynthetic microorganisms using redox reactions with energetic yields to recycle inorganic carbon,^33^ and was determined by the incorporation of ^14^CO_2_. More details of HBP and DCF measurements are described in Supporting Information.

### Statistical Analysis

Principal component analysis (PCA) was performed using Simca-P (version 11.5, UmetricsAB, Umeå, Sweden). Hierarchical cluster analysis (HCA) used Hierarchical Clustering Explorer 3.0. Details are described in Supporting Information.

## Results

### Changes in DOM Molecular Composition after S + N Mixing in FT-ICR MS

Mass spectra of Solimões (S) and Negro River (N) SPE-DOM showed skewed near-Gaussian distributions for thousands of mass peaks ranging from *m*/*z* 150–950 (Figure S1), indicative of high molecular diversity.^5,10,11,34^ However, mass spectra of S + N SPE-DOM developed recognizable patterns of putative microbial-derived signatures, showed a large-scale shift to lower *m*/*z* in ESI[+] mass spectra (Figure S1), and had a considerably lower number of assigned molecular formulae in ESI[±] mass spectra (Tables S3 and S4). The average *m*/*z* of S + N SPE-DOM was significantly lower than that of *S* and *N* SPE-DOM, while its average atomic ratios (e.g., O/C, H/C, S/C, and N/C) varied more slightly after mixing (Tables S3 and S4).

**Figure 2:**
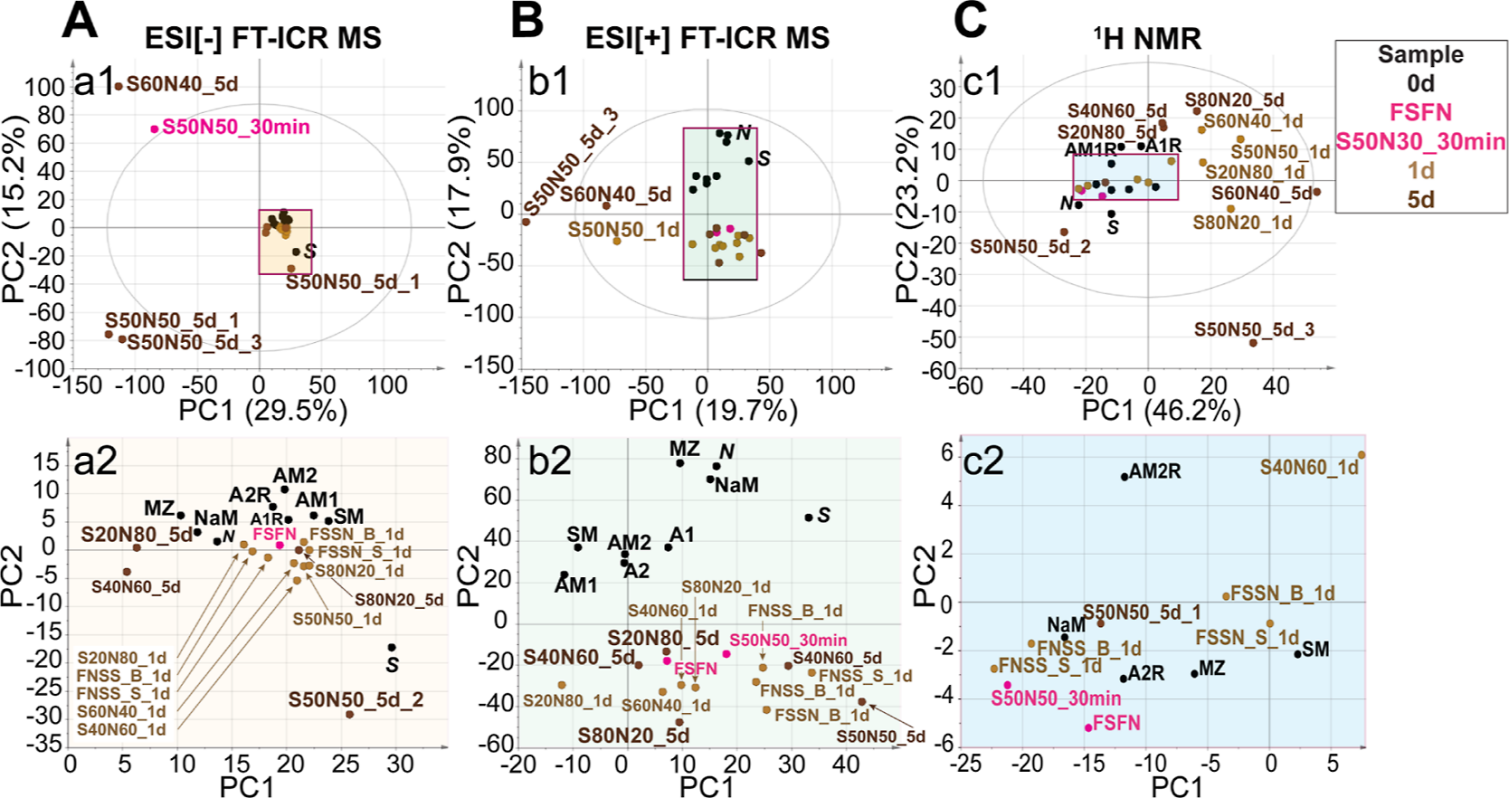
PCA of assigned SPE-DOM molecular formulae in unmixed and mixed S + N waters pre- and post-incubation, according to sample analysis methods (A) ESI[-] and (B) ESI[+] FT-ICR mass spectrometry and (C) ^1^H NMR spectroscopy. a2/b2/c2 depict an enlarged view of samples in the purple rectangles in a1/b1/c1. Loading vectors are shown in Figure S2.

To assess major variation in DOM, we performed PCA on its assigned molecular formulae (Figure 2A,B). S60N40_5d and S50N50_5d were separated by the first principal component (PC1) of ESI[−] and ESI[+] MS-based PCA because of the distinct abundance of less oxygenated compounds, in accordance with HCA (see Figure S3 and its footnote). The second principal component (PC2) of ESI[−] MS-based PCA placed S + N between S and other unmixed samples, whereas PC2 of ESI[+] MS-based PCA showed clear separation of river SPE-DOM and incubated S + N SPE-DOM due to the decrease of high molecular weight CHO and CHNO compounds. However, the mixing ratio of endmembers (*S* and *N*) did not show a link to the PCA distribution of incubated S + N. Moreover, the original samples other than *S* and *N* showed higher PC2 in ESI[−] MS-based PCA and were placed between endmembers (*S* and *N*) and incubated with S + N in PC2 of ESI[+] MS-based PCA. Furthermore, the 1d- and 5d-incubated samples were not distinct in PCA and HCA (Figures 2A,B and S3), suggesting that S + N SPE-DOM composition did not record a continuous trend in temporal evolution.

We observed a remarkable loss of molecular signatures after S + N mixing, with around half of molecular formulae disappearing immediately after mixing (Figure S4). Additionally, no unique molecular formulae were found in 0d-/1d-/5d-incubated SPE-DOM that were not present in *S* and *N* endmembers of SPE-DOM. Hence, no new molecular formulae appeared between 1d and 5d of incubation, which may reflect fast DOM transformation within 1d.

### Changes in DOM Molecular Composition after S + N Mixing in ^1^H NMR Spectra

^1^H NMR spectra of *S* and *N* SPE-DOM showed smooth bulk envelopes, reflecting superpositions of millions of carbon atomic environments typical of processed aqueous DOM,^15^ whereas S + N SPE-DOM showed a large number of additional superimposed small resonances (Figure S5). Although, ^1^H NMR section integrals of half incubated S + N SPE-DOM were placed approximately in the middle of endmember samples *S* and *N* (Table S6), the other half showed a higher abundance of pure and functionalized aliphatic protons (δ_H_ ∼ 0.5–2.35 ppm), at the expense of oxygenated aliphatic and unsaturated protons (δ_H_ > 3.1 ppm) (Figures 2C and S2C and Table S6). The incubated S + N samples were separated in PC2 of ^1^H NMR-PCA due to the different content of aromatic protons (δ_H_ > 6.6 ppm) (Figures 2C and S2C).

Difference ^1^H NMR spectra of endmember *S* and *N* SPE-DOM revealed a higher abundance of pure aliphatics (CCC**H** units), a lower relative abundance of aromatic (C_ar_**H**, especially polyphenols), direct (OC**H**), and remotely oxygenated (OCC**H**) units in *S*, compared with *N* SPE-DOM (Figures S6A and S7A). Furthermore, more extensively oxygenated aliphatic and aromatic C**H** units were more abundant in *S* than in *N* SPE-DOM. The river samples A1 and A2 downstream of the S + N confluence showed an increase of methylene and alkyl protons (δ_H_ ∼ 1.2 ppm), as well as branched and functionalized aliphatics, including simple carboxylic acids (δ_H_ ∼ 1.2–2.1 ppm), polyols (δ_H_ ∼ 3.2–3.6 ppm), and a decline of highly oxygenated aliphatics (δ_H_ > 3.6 ppm) and C_sp2_**H** units (δ_H_ > 6 ppm) (Figure S8).

After mixing *S* and *N* for 30 min, visible increases of branched purely aliphatic molecules (δ_H_: 1.3–1.5 ppm), some carboxylic acids (δ_H_ ∼ 2.0–2.4 ppm, methyl ethers (δ_H_: 3.6–3.75 ppm) and olefins (δ_H_ ∼ 5.6–6.5 ppm) were observed (Figures S6B and S7B). After 1d, a very substantial increase of pure and functionalized aliphatics (CCC**H** and OCC**H** units, indicative of carboxylic acids), equivalent to ∼50% ^1^H NMR section integral, had occurred across the board (Figures 5A, S6C); the relative abundance of oxygenated aliphatic units (OC**H** units) decreased, while a suite of alkyl and carboxylated aromatic molecules increased (Figures 5A, S6C, and S7C). From 1d to 5d, a remarkable reversal of DOM processing had, in essence, restored the initial status of (S + N) SPE-DOM, with an increase in pure and functionalized aliphatics (δ_H_ ∼ 0.5–2.4 ppm), loss of methoxy ethers and esters (δ_H_ ∼ 3.4–4.0 ppm), and about all other directly and remotely oxygenated aliphatic molecules (δ_H_ > 2.4 ppm) (Figure S6E). Hence, much of the initially produced aliphatic molecules were labile under the given conditions and became either consumed or reoxidized within four days.

### Effects of Particles in S + N Mixing and 1d Incubation

**Figure 3:**
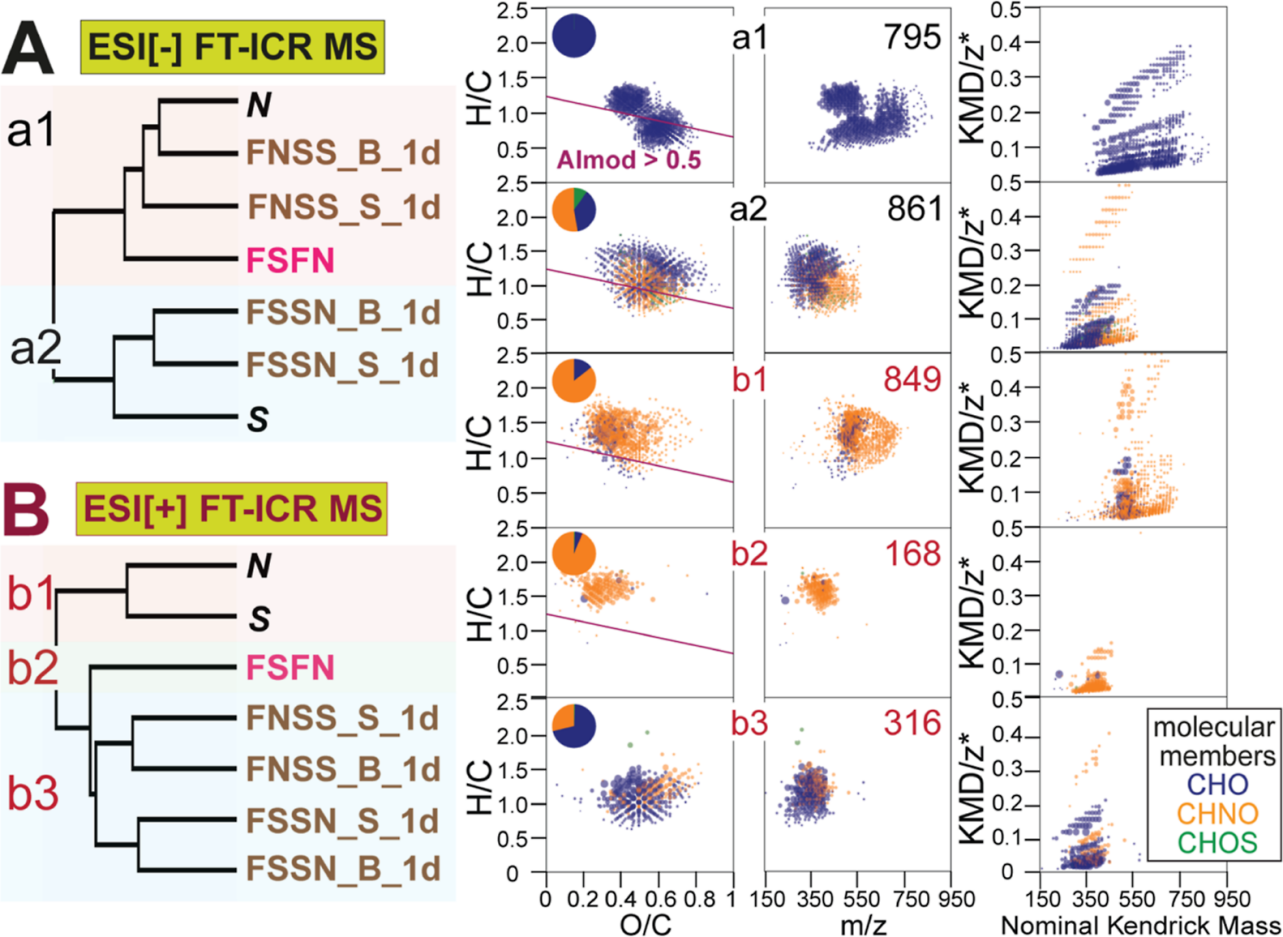
HCA of assigned SPE-DOM molecular formulae in part of the unmixed and mixed S + N waters pre- and post-incubation; (A) ESI[−] and (B) ESI[+] FT-ICR MS. Van Krevelen, mass-edited H/C, and KMD/z* diagrams^35,36^ show CHO (blue), CHNO (orange), and CHOS (green) molecular classes that were relatively more abundant in clusters a1/a2, b1/b2/b3, respectively. The pie plots depict percentages of the counts of different molecular classes. The numbers show counts of molecular compositions. The molecular formulae positioned below the purple line in the van Krevelen diagrams have a modified aromaticity index (AI_mod_)^37^ higher than 0.5.

The clustering in ESI[−] MS-based HCA indicated that the contact between water and suspended particles had negligible effects on DOM composition (Figure 3A). FSFN showed a higher similarity to *N* than to *S*, conforming to the higher concentration of DOC (dissolved organic carbon) in Negro (9.96 mg/L) than in Solimões (4.56 mg/L; Table S5). ESI[+] MS-based HCA separated *S* and *N*, FSFN, 1d incubated S + N into three clusters, with distinction of FSSN samples alike ESI[−] derived HCA (Figure 3), suggesting that degradation of some more saturated, lower molecular weight CHNO compounds and synthesis of lower mass (*m*/*z* < 450) CHO compounds with average H/C and O/C ratios occurred during 1d incubation (Figure 3b2).

### Changes in DOM Molecular Composition after A + T Mixing in FT-ICR MS

ESI[±] FT-ICR mass spectra of A + T SPE-DOM developed intense mass peaks superimposed to the bulk signature (Figure S9), indicative of efficient production of specific molecular signatures in parallel with large-scale transformation of DOM molecules. The average *m*/*z*, average O/C ratio, and count of molecular formulae substantially declined, whereas the average H/C ratio increased after the 5d-incubation (Tables S7 and S8).

**Figure 4:**
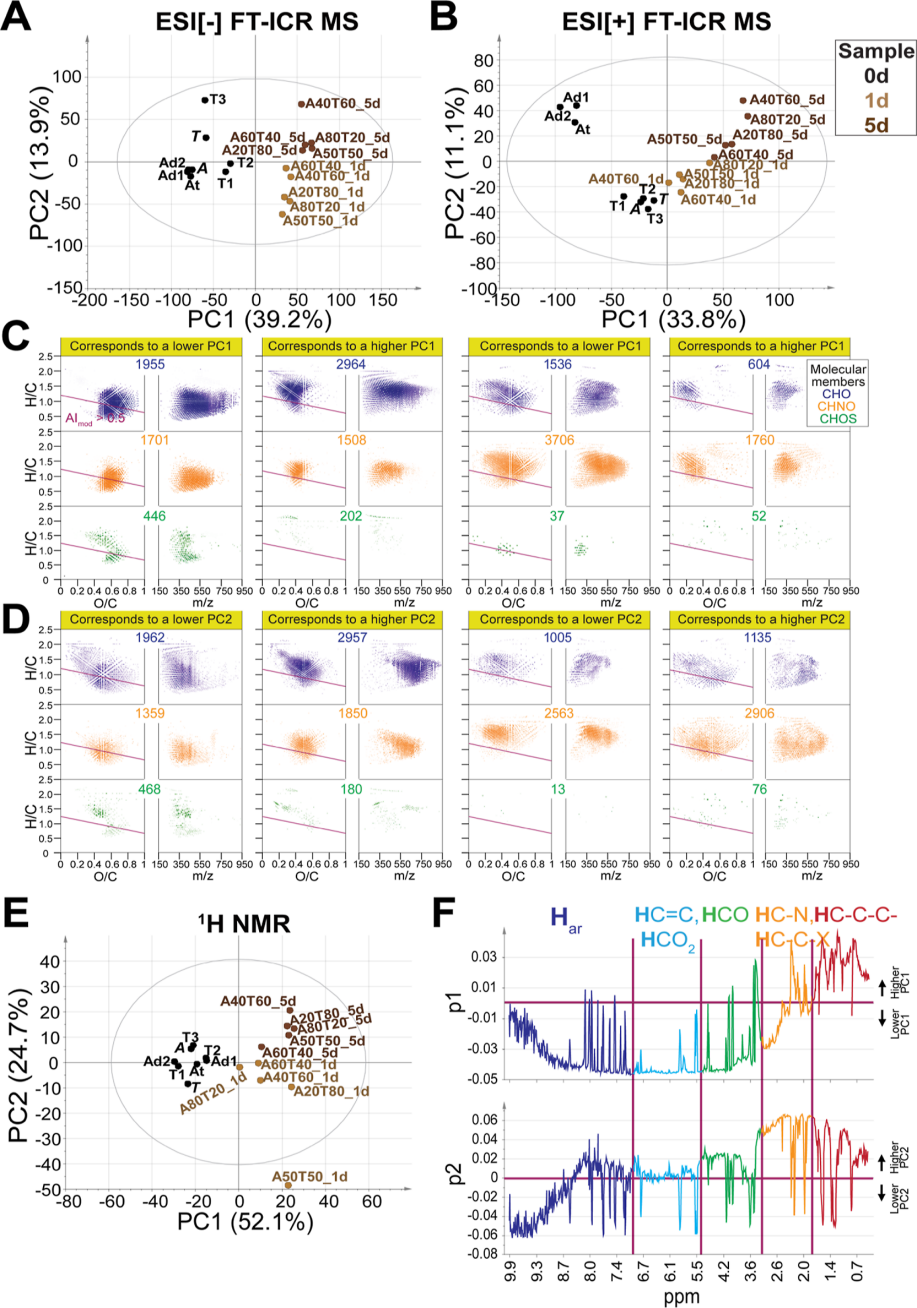
PCA of assigned SPE-DOM molecular formulae in unmixed and mixed A + T waters pre- and post-incubation. Panels (A,B,E) show PCA scatter plots. Panels (C,D) show correlation patterns of PC1 and PC2 loading vectors (p1 and p2) in van Krevelen and mass-edited H/C diagrams, with the color code representing CHO (blue), CHNO (orange), and CHOS (green) molecular classes. The numbers show counts of compounds. The molecular formulae positioned below the purple line in the van Krevelen diagrams have a modified aromaticity index (AI_mod_)^37^ higher than 0.5. Panel F shows the correlation patterns of p1 and p2 in ^1^H NMR space, with fundamental molecular structures indicated.

In both ESI[−] and ESI[+] MS-based PCA (Figure 4), PC1 showed a clear trend: Amazon < Tapajós < 1d-incubated A + T < 5d-incubated A + T, due to increased abundance in compounds with a lower O/C ratio, a lower *m*/*z*, and a modified aromaticity index (AI_mod_)^37^ less than 0.5. Moreover, 1d-incubated A + T < 5d-incubated A + T were separated by PC2 and had different abundances in high molecular weight CHO and CHNO molecules. The molecular patterns indicated extensive transformation from more oxygenated, high mole-cular weight, higher aromaticity compounds to less oxygenated, low molecular weight, higher aliphaticity compounds during 5d-incubation. Additionally, *A* was separated from other Amazon samples and showed high similarity to Tapajós samples in ESI[+] MS-based PCA. Moreover, the mixing ratio of endmembers (*A* and *T*) did not show a link to the PCA distribution of incubated A + T, which was consistent with the result of mixing S + N.

HCA clearly separated unmixed and mixed A + T samples (Figure S10), in line with PCA results. 1118 oxygen-rich and polyphenolic CHO/CHNO compounds decreased, whereas 1228 more aliphatic and more saturated CHO/CHNO compounds increased in relative abundance after the 1d-/5d-incubation. KMD/z* diagrams revealed extended CH_2_-based homologous series for CHO compounds that increased/decreased during incubation and shorter ones for CHNO compounds (Figure S10).

We found that nearly half of the molecular formulae disappeared after A + T mixing and 1d-incubation, in line with S + N mixing (Figure S11). The count of molecular formulae that disappeared and newly appeared after A + T mixing were about equal (∼3000). Furthermore, ∼1000 molecular signatures remained after 1d and disappeared after 5d (Figure S11a4,b4).

### Changes in DOM Molecular Composition after A + T Mixing in ^1^H NMR

Incubation caused the emergence of sharp ^1^H NMR resonances of putative biotic origin, which contributed ∼3% to the total ^1^H NMR integral in 1d incubated A + T SPE-DOM and ∼1% in 5d incubated A + T SPE-DOM (Figure S12). The 1d incubation produced strong ^1^H NMR resonances containing methylene-rich aliphatic molecules (δ_H_ ∼ 1.25 ppm), an admixture of aliphatic branching (δ_H_ ∼ 1.3–1.5 ppm) that is probably associated with carboxylic acids RCOOH (HOOC-C**H**α-C**H**β; δ_H_ ∼ 2.3–2.45 ppm; 1.6–1.75 ppm), olefins (δ_H_ ∼ 5.2–5.5 ppm/5.8 ± 0.1 ppm), and carboxylated benzene derivatives (δ_H_ > 7.3 ppm)^38^ (Figures S13B and S14B).

The abundance of purely aliphatic protons (CCC**H** units; δ_H_ ∼ 0.5–1.9 ppm) largely increased within 1d from 34% to 42% at the expense of about all other units (Table S9). The carboxylic acids with supposedly more “simple” aliphatic branching motifs that were produced within 1d declined between 1d and 5d (Figure S13D). So, temporal evolution SPE-DOM from 1d to 5d of S + N and A + T shared aspects of synthesis after 1d and receding to the original state after 5d. However, alterations within A + T concerned a rather limited suite of methylene-rich molecules in comparison with S + N which concerned an overall change in different molecular structures.

PC1 and PC2 in ^1^H NMR-based PCA explained ∼77% of the total variance (Figure 4E) and showed similar separation compared to ESI[−] MS-based PCA (Figure 4A). The mixing ratios of endmembers did not show an impact on the molecular composition of incubated A + T SPE-DOM from ^1^H NMR-based PCA.

### Changes in Microbial Activity after S + N and A + T Mixing

We measured the rates of two microbial metabolic processes in unmixed and mixed waters. HBP refers to assimilatory metabolism consuming preferentially labile DOM-like proteins,^32^ and DCF refers to light-independent inorganic carbon uptake mostly performed by chemosynthetic microorganisms.^33^ HBP in endmembers *S*, *N*, and in the S + N mixing zone, as well as in the one-hour incubated equal volume S + N mixtures, remained nearly constant around 0.03 μgC L^–1^ h^–1^ (Table S10). However, HBP in A1 and A2 downstream of the S + N confluence showed over 100 times higher HBP rates. The HBP rates in endmembers *A*, *T*, and in the A + T mixing zone were around 0.15 μgC L^–1^ h^–1^, approximately five times higher than in the S + N mixing zone, in line with previous research that showed high rates of microbial respiration and bacterial growth in the Amazon-Tapajós confluence.^39,40^

The rates of DCF were much higher than HBP in all the water samples, with ∼200 times higher in S + N confluence and ∼10 times higher in A + T confluence. DCF rates were two times higher in *N* (1.66 μgC L^–1^ h^–1^) compared to *S* (0.76 μgC L^–1^ h^–1^) and increased ∼10 times in the S + N mixing zone. DCF rates in *A* and *T* were ∼0.45 μgC L^–1^ h^–1^ and increased ∼4 times in the A + T mixing zone.

Our measurements of HBP and DCF in the Amazon River are consistent with rates observed in other aquatic systems worldwide (Table S11). Our observations of biomass production ranging from nanograms to milligrams of carbon per hour are similar to those reported in previous studies conducted in the Amazon and other aquatic systems. Earlier studies in the Amazon have reported HBP varying from 0.1 to 7 μgC L^–1^ h^–1^, indicating higher values during high waters^12,41−43^ and in mixing zones.^12^ We observed our highest HBP in the Amazon River downstream of the confluence of the Tapajos River, which has been associated with increased rates of phytoplankton productivity, including labile organic compounds for microbial growth.^43^ On the other hand, we observed the highest DCF in the Amazon River after the confluence of the Negro and Solimoes Rivers, where we observed a high concentration of DIC of approximately 1300 μM and high assimilatory ratios. The mixing zone is often reported as a hotspot for microbial processes due to the mixing of microbial communities and river compounds,^12,13^ creating opportunities for high rates of carbon assimilation via distinct processes, including chemosynthesis using the energetic yields of oxidation of reduced compounds.^42^ Although our survey represents all available measurements in the Amazon River, we found other studies in different aquatic systems where rates vary from nanograms of carbon per hour in lakes.^44−46^ High rates of DCF were observed in karstic systems,^47−49^ which could be related to the high concentration of inorganic carbon and reduced compounds in soil and groundwater, creating opportunities for microbial growth using the energetic yields of redox reactions to promote chemosynthesis since HBP was observed at lower rates in these systems.^50,51^ In river estuaries, turbulent mixing promotes an increase in DCF^47,52,53^ and HBP,^54−56^ which could also be associated with reduced compounds from bottom sediments meeting oxic surface waters.

## Discussion

### Molecular Characteristics of Endmembers

Blackwater *N* DOM showed higher average *m/z* ratios (Tables S3 and S4) and higher aromaticity (Table S6 and Figure S5) compared to DOM of other rivers, in agreement with previous studies.^5,57^ Ertel et al. assigned 70% of degraded humic and fulvic acid fluxes at the S + N confluence to Negro inputs.^57,58^ High molecular weight aromatic structures probably represent the polar fraction of lignin and tannin degradation products, e.g., through biotic and abiotic oxidation.^58,59^*N* DOM showed higher proportions of =C**H** and C_ar_**H** units (δ_H_ > 5.2 ppm) and remotely (OCC**H**) and directly oxygenated (OC**H**) aliphatic molecules (δ_H_ > 2.4 ppm) (Table S6 and Figure S5).

*S* and *A* SPE-DOM showed higher average N/C and S/C ratios and had a larger contribution of moderately unsaturated and saturated compounds (Tables S3 and S4). Nitrogen-containing compounds could be preferentially sorbed and transported by the sediment-rich waters.^60^

Clearwater *T* SPE-DOM showed higher proportions of CHNO compounds and methylene- and alkyl-rich aliphatic structures (Figure S13 and Table S9) than other endmember DOM (Tables S7 and S8), in line with higher proportions of primary production and microbial processing.^11^^1^H NMR spectra of *T* SPE-DOM showed a distinct visual appearance, owing to a few sharp and abundant resonances representing aliphatic molecules with limited branching, acetic acid (δ_H_ ∼ 1.88 ppm) and formic acid (δ_H_ ∼ 8.08 ppm), with an overall ^1^H NMR integral below ∼2% (Figure S12). The strong contribution of small aliphatic molecules in *T* DOM may originate from the general proximity of its DOM molecules to the structural space of biomolecules and metabolites,^11^ including some biodegradation of fresh materials, as suggested by Roth et al.^61^

The endmember samples *S*, *N*, *A*, and *T* showed distinct characteristics, including the intensity distribution of H/C, O/C, N/C, S/C ratios, *m*/*z*, aliphaticity, and aromaticity, in line with previous reports.^12,19^ Furthermore, ^1^H NMR spectra showed the main distinction in relative abundance of methoxy-related substructures in the order *S* > *N* (Figure S6A) and *A* > *T* (Figures S13A and S14A). The main distinction between *S* and *N* SPE-DOM largely covered methyl ethers (δ_H_ ∼ 3.6–3.8 ppm), whereas the difference between *A* and *T* SPE-DOM covered methyl esters and methyl ethers (δ_H_ ∼ 3.6–4.1 ppm).^15^ This may imply distinct initial abundance and processing of more “ether-rich” lignins and more “ester-rich” tannins,^62^ but the scavenging of reactive DOM molecules by methanol^63^ cannot be excluded.

### Distinct Structural Evolution after S + N and A + T Mixing

**Figure 5:**
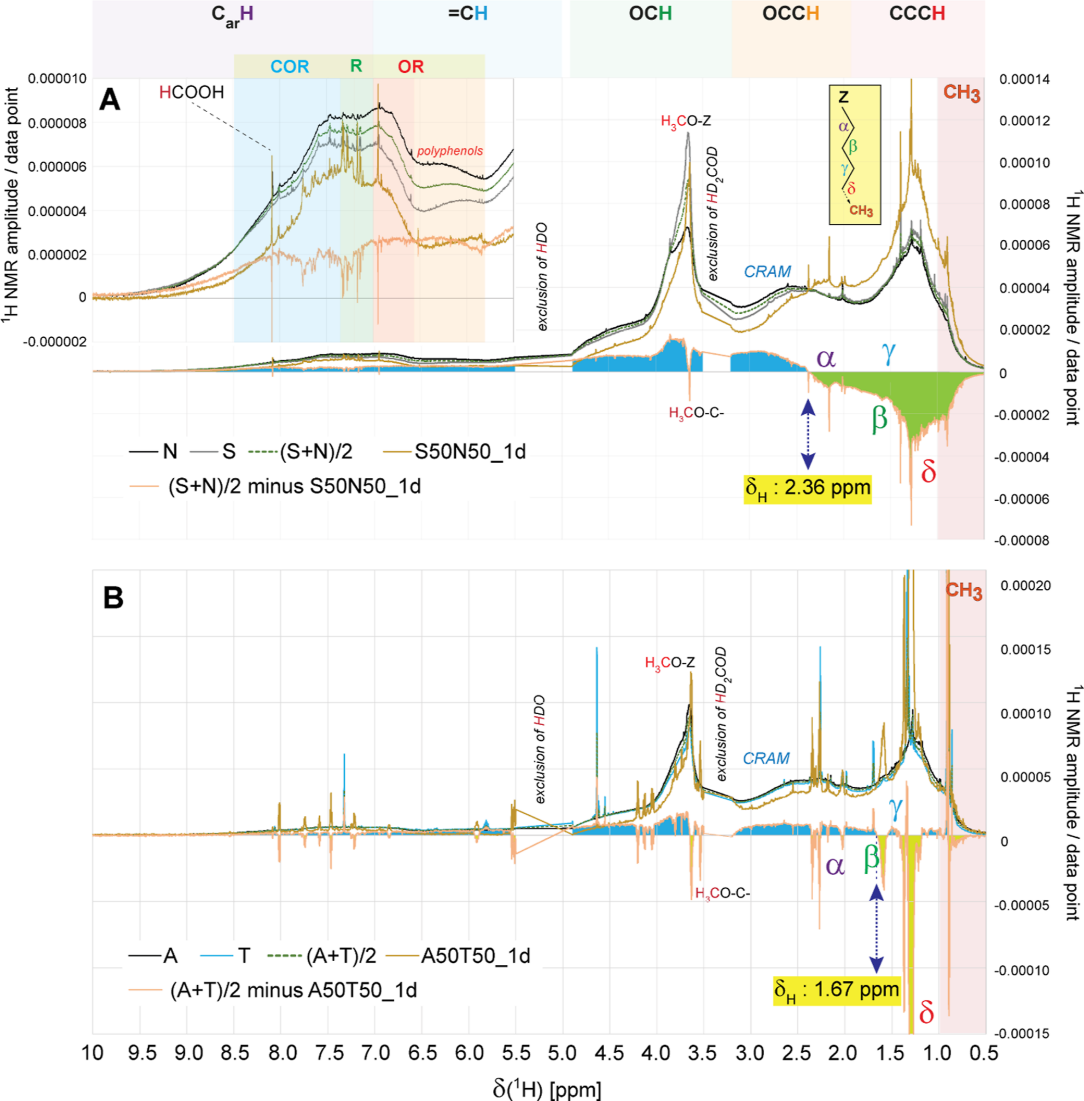
Different ^1^H NMR spectra of (A) (S + N)/2 minus S50N50_1d and (B) (A + T)/2 minus A50T50_1d, with annotations of key substructures, demonstrate the fundamental distinction in molecular evolution of S + N and A + T after 1d-incubation (cf. text). Incubation of S + N caused alterations across all atomic environments, whereas incubation of A + T referred primarily to a limited set of basic methylene- and alkyl-rich aliphatic molecules (cf. text). Yellow-shaded δ_H_ denotes a significant crossover of aliphatic molecules which were consumed (blue shade) or produced (green shade) after 1d, denoting a higher molecular diversity and degree of functionalization in newly produced S + N molecules compared with those of A + T.

In A + T, a suite of labile lipid-like molecules with prominent alkyl- and methylene-rich carboxylic acids of limited structural diversity and few oxygenated aliphatic and aromatic structural motifs had been produced after 1d and largely disappeared after 5d of incubation. In comparison, the structural changes after 1d incubation of S + N DOM comprised a much larger structural diversity of molecules, covering the entire range of aliphatic (CCC**H**, OCC**H**, and OC**H** units) and C_sp2_**H** units (aromatics and olefins) with a smooth distribution of all structural motifs (atomic environments), implying a more thorough diversification of atomic environments in S + N compared with A + T following mixing and incubation. This probably reflects an overall larger distinction of DOM molecules in original *S* and *N* waters compared with *A* and *T* waters, originating from the high relative abundance of highly oxygenated aromatic structures in *N* waters (Table S6). Gain was observed for a very wide range of pure and functionalized aliphatic units (δ_H_ ∼ 0.5–2.36 ppm), and loss was observed for all other atomic environments in the case of S + N incubation (Figure 5A). While the rather high proportions of the above-mentioned methylene- and alkyl-rich molecules produced after 1d in the case of A + T incubation may have masked other alterations on visual inspection of ^1^H NMR spectra, appreciable distinction between 1d incubation for A + T versus S + N also applied for less intense ^1^H NMR resonances. The crossover points for difference spectra “endmember minus 1d incubation” was displaced from δ_H_ ∼ 2.36 ppm (S + N) to δ_H_ ∼ 1.67 ppm (A + T), implying that less oxygenated aliphatic molecules were preferentially degraded after 1d incubation in the case of A + T. The structural evolution from 1d to 5d incubation was in essence a reversal of the structural changes from original samples to 1d incubation for both S + N and A + T mixing experiments: A + T samples diversified their pure and remotely oxygenated aliphatic molecular structures (δ_H_ ∼ 0.5–3 ppm) and lost methylene- and alkyl-rich aliphatic molecules with simple branching motifs (Figure S12); at day 5, A + T DOM comprised higher proportions of pure aliphatic molecules (δ_H_ ∼ 0.8–1.7 ppm) and lower shares of methoxy groups (δ_H_ ∼ 3.5–4.1 ppm) than the average of A + T (Figure S12C). For S + N, the evolution from 1d to 5d incubation appeared as a very congruent reversal compared with the evolution from average S + N to 1d incubation for all aliphatic units (δ_H_ ∼ 0.5–5 ppm (Figure S6). The distinction between the average of S + N and 5d incubation were very minor, like the small increase of alkyls C_*n*_H_2*n*+1_ (δ_H_ ∼ 0.5–1.0 ppm) and methoxy groups (δ_H_ ∼ 3.4–3.7 ppm) (Figure S6D,E). This remarkably fast processing of DOM for both mixing experiments has likely been promoted by tropical temperatures (∼30 °C).

The large-scale depletion of high molecular weight compounds after mixing was significant for both S + N and A + T, especially for nitrogen-containing compounds that were better ionized in ESI[+] MS (Figure S1 and Table S7). This shift of mass was also pronounced for FSFN and S50N50_30min, suggesting an immediate change following mixing. Unlike A + T SPE-DOM that showed continuous molecular changes over 5d (Figure 4), S + N SPE-DOM changed immediately after mixing but showed no further continuous trends over 5d (Figure 2). This sudden change in DOM properties probably results from the abiotic, preferential sorption of hydrophilic, polyphenol-rich Negro DOM by Solimões suspended particles.^10,64,65^ Additionally, the large depletion of POC (particulate organic carbon) at Solimões-Negro mixing zone suggested that the DOC-POC transition significantly affected the DOM composition in the confluence (Table S5). Coagulation heterogeneity, including the coating of minerals with specific DOM fractions and microbes,^66^ likely contributed to the observed heterogeneity in S + N DOM (Figure 2).

We observed conservative behavior of DOC concentration downstream of the S + N confluence, in line with two previous studies,^10,67^ which suggest that organic nanoparticulate or colloidal matter did not readily coagulate to form larger aggregates during mixing.^67^ Another reason suggested by Simon et al. is the occupation of mineral sorption sites by DOM molecules.^10^

The abundant *m*/*z* ions in incubated A + T SPE-DOM occupied van Krevelen positions congruent with the abundant mass peaks in phytoplankton and microbial exudates (Figure S10a2,b2).^68,69^ The Tapajós River hosts higher proportions of cyanobacteria (∼12%) compared to the Amazon River (∼5%), where actinobacteria and betaproteobacteria dominate the microbial community.^70^ “Positive priming effect” was suggested to happen when algal-rich Tapajós waters mix with turbid Amazon main stem waters, accelerating the decomposition of more recalcitrant organic matter.^32^ The molecular transformation of CHO, CHNO, and CHOS molecules toward lower *m/z* and lower O/C ratios with concomitantly higher H/C ratios in A + T SPE-DOM during 5d incubation is consistent with a simple notion of DOM degradation from larger to smaller molecules, which was expressed in the familiar size-reactivity continuum model,^71^ but here, it expands to relatively small DOM molecules and/or metabolites. Moreover, ^1^H NMR spectra indicated that polyphenolic molecules were largely removed in A + T SPE-DOM after 5d (Figure S12B and Table S9), suggesting rapid processing of terrestrial organic matter (e.g., leached from litter decay processes) that had been largely hydrologically mobilized in aquatic–terrestrial transition zones.^72,73^ This conforms to a study showing that the breakdown of vascular plant-derived organic matter to CO_2_ increased six-fold at the Amazon-Tapajós confluence compared to that in the Amazon River.^74^

### Non-conservative DOM Behavior after Mixing

A study by Simon and co-workers observed a conservative behavior of SPE-DOM in the natural and experimental mixing of Solimões and Negro river waters based on negative ionization mode FT-ICR MS,^10^ opposing the often-presumed non-conservative behavior of DOM and our results. In our study, the mixing ratios of both endmembers (S + N and A + T) did not show linear correlations with DOM composition based on ESI[±] FT-ICR MS and ^1^H NMR spectra. Simon et al. sampled during the dry period, while the samples from this study were taken during a maximum flood pulse. The different sampling season presumably affects composition and structure of dissolved constituents leached from rewetted soils and may influence the endmember water characteristics and microbial community.^4^ Moreover, Simon and co-workers mixed samples under continuous shaking, while we mixed initially and then let the sample settle, incubated for 1d and/or 5d. The differences in sample treatment may affect DOM composition, for instance through sorption and desorption between DOM and particles. Besides, the combination of SPE and ESI[−] FT-ICR MS favors observance of carboxylic acids and does not cover the entire spectrum of dissolved organic molecules.^75−77^ Our joint use of both complementary electrospray modes FT-ICR MS and ^1^H NMR methods and the exhaustive analysis provide valuable DOM molecular information with improved detail. ESI[−] FT-ICR MS preferentially detected high-mass oxygen-rich CHO compounds, whereas ESI[+] FT-ICR MS primarily detected aliphatic CHNO compounds.^34^ Our previous study on the SPE-DOM in Amazon main stem and tributaries showed that nearly half of CHO and CHNO *m*/*z* ions were found in both ESI modes at near-average H/C and O/C ratios.^11^ Moreover, 800 MHz ^1^H NMR spectra provided near-quantitative data of major atomic environments in DOM molecules with excellent S/N ratio and resolution, supplying relevant information about the fate of aliphatic and aromatic moieties that are poorly ionized in ESI mass spectrometry.^78^

### Effects of Biotic and Abiotic Factors on DOM Transformation

Abiotic reactions, such as photochemical processing (oxidation and mineralization) and metal-dependent complexation, coagulation, and redox chemistry, will have significant impact on the DOM composition in the Amazon River.^21,64^ Structure-selective sorption of DOM on mineral surfaces^8,11^ has removed substantial proportions of polyphenols present in N waters together with minerals present in S waters. This removal of light absorbing and light scattering ingredients in the S + N mixing zone opens previously unavailable opportunities for photo-processing of DOM molecules. However, many photoproduced oxygenated small molecules will readily integrate in biochemical pathways, foodwebs,^22,25^ and transient particulate organic carbon, making traceability to specific mechanisms difficult.

Multistep processing of DOM along the flow path and mixing zones of many ungauged Amazon River tributaries implies that the carbon atoms that are eventually delivered to the Atlantic Ocean have repeatedly changed their atomic environments on their journey.^61^ However, general congruence of biochemical pathways across all organisms, and similarities of fundamental abiotic processing regimes in the extended tropical ecosystem of the Amazon basin will rather realign thermodynamic and kinetic boundary conditions at locations of exceptional importance, leading to thorough redirection for the synthesis and degradation of DOM molecules. This is more likely than an unspecific continual entropy-driven diversification of DOM molecules from land to sea. Overall, DOM evolution in the Amazon Basin will diversify beyond simple trajectories of synthesis and degradation, as described in the standard river continuum concept^79^ and/or small river catchments.^80^ Nevertheless, we expect that a certain set of rather unreactive and difficult to observe aliphatic molecules with structural features resisting expedient biotic and abiotic degradation might become selectively enriched along the Amazon River flow path. Plausible structural features comprise fused and bridged polycycloalkyls with structural oxygen and nitrogen atoms connected to more than a single carbon atom, overall distant from common biomolecular binding motifs that are subject to expedient enzymatic degradation.

### Environmental Implications

According to the ‘‘regional chromatography” hypothesis,^57,81^ the majority of reactive components within the DOC pool are attenuated by minerals before reaching higher-order confluences, such as the S + N confluence. We hypothesized that individual *S*, *N*, *A*, and *T* Amazon DOM had reached a steady state under given biogeochemical conditions. However, the assemblages of biogeochemically distinct river waters initiated rapid and widespread molecular alteration of DOM upon mixing, in part by fast abiotic reactions. This averaged physiochemical properties and reassembled microbial community composition, with potentially considerable effects on DOM processing and mineralization, as well as the composition and reactivity of remaining organic molecules.^82^ Pronounced changes in DOM composition during incubation suggest substantial diagenetic modification of organic matter, even if the complete mineralization of DOM may not be significant.^83,84^ Nevertheless, continuous oxidation of the residual DOM molecules is likely to generate oxygen-rich CO_2_ and H_2_O molecules in the river confluence and subsequent river channels, contributing to a nominal reduction of leftover DOM molecules even in the case of oxidative processing.^84^

Rivers provide a direct link between terrestrial and marine carbon cycles. However, very little terrestrial DOM appears to accumulate in the global ocean.^85−87^ Incubation experiments with turbid water from close to the river mouth suggested that photo- and bio-alteration leave significant molecular and carbon isotopic imprints on the terrigenous DOM. Nonetheless, quantitative removal by bio- and photo-degradation appeared to proceed at a relatively slow pace because no significant decrease of dissolved organic matter was found within five days of the incubation.^32^ However, 9–30% of DOC was lost after the five-day incubation with less turbid water from the intermediate and outer plume, suggesting that the introduction of reactive algal DOM in the intermediate plume may thus have primed the microbial degradation of terrigenous DOM.^32,88^ Additionally, sorption of terrigenous DOM to sinking particles acts as an important DOC sink in the Amazon plume.^32^ These results, along with our results, demonstrate that the DOM along the Amazon River-to-ocean continuum was not in a steady state as suggested by the “regional chromatography” hypothesis. Moreover, river confluences and a less turbid offshore plume are likely important carbon sinks in the Amazon River due to biotic processes (e.g., biodegradation) and abiotic processes (e.g., photodegradation and sorption to particles).

The higher heterotrophic bacterial activity in A + T compared with S + N (Table S10) suggested that the molecular changes in A + T SPE-DOM during 5d-incubation referred more to bioavailable alkyl-rich molecules than in the case of S + N, which were more oxygenated on average. Moreover, DCF rates noticeably increased in both mixing experiments and amounted up to ∼95% of the total HBP after the mixing. The results indicate that non-photosynthetic carbon fixation can represent a substantial contribution to an autochthonous source of organic matter in river confluences.^89^ DCF had been reported to contribute approximately 80% of the total HBP in Swedish boreal lake sediments.^33^ Moreover, it has been shown that the heterotrophic metabolism of bacteria is suddenly intensified after the input of organic matter.^90^ The water–particle interface in river confluences could be sites of intensive biogeochemical activity, creating steep chemical gradients that provide a microenvironment with a high chemolithotrophic rate.^91^ The simultaneous presence of oxidized and reduced organic molecules supplies energy and substrates needed to support DCF by chemoautotrophic microbes.

## Conclusions

Ecological models and biogeochemical characterization have established river mixing zones as hotspots of microbial activity, but molecular DOM degradation pathways are still poorly addressed in river networks, preventing an accurate estimate of carbon sources and sinks. We observed extensive but molecularly distinct structural alterations of DOM upon mixing and follow-up incubation of Solimões/Negro on the one hand and Tapajós/Amazon waters on the other hand. Molecular distinction of DOM structural evolution will apply in essence to all river conflucences, and educated modeling eventually has to take these mechanistic details of DOM processing into account.

Relevant molecular resolution concerning the fate, transport, and transformation of DOM in natural and engineered systems is not attained without the use of complementary high-resolution structure-selective analytical and biogeochemical methods. Characterization of molecularly heterogeneous and polydisperse DOM by complementary structural spectroscopy like, e.g., [±]ESI mass spectrometry and NMR spectroscopy provides distinct aspects of its composition and structure. In this study, [+]ESI mass spectra provided relevant coverage of CHNO molecules, ^1^H NMR spectra demonstrated major distinctions in the structural evolution of DOM in the two S + N and A + T mixing zones, not visible in mass spectra. Joint use of structural and classical biogeochemical analysis of DOM molecular evolution will clearly diversify our perception and help us appreciate the specificity of DOM processing according to sampling points. Unifying these informative findings will remain a challenge for integrative modeling studies of the global carbon and other element cycles.

## Supporting Information Available

Details of sampling, analyses, interpretations, ESI[-] and ESI[+] FT-ICR mass spectra; loading vectors; water parameters; HCA; common and unique molecular signatures; ^1^H NMR spectra; ^1^H NMR section integrals; difference ^1^H NMR spectra; and HBP and DCF (PDF)

## References

[ref1] Hess L. L.; Melack J. M.; Affonso A. G.; Barbosa C.; Gastil-Buhl M.; Novo E. M. L. M. Wetlands of the lowland Amazon basin: Extent, vegetative cover, and dual-season inundated area as mapped with JERS-1 synthetic aperture radar. Wetlands 2015, 35, 745–756. 10.1007/s13157-015-0666-y.

[ref2] Richey J. E.; Hedges J. I.; Devol A. H.; Quay P. D.; Victoria R.; Martinelli L.; Forsberg B. R. Biogeochemistry of carbon in the Amazon River. Limnol. Oceanogr. 1990, 35, 352–371. 10.4319/lo.1990.35.2.0352.

[ref3] Junk W. J.; Piedade M. T. F.; Schöngart J.; Wittmann F. A classification of major natural habitats of Amazonian white-water river floodplains (várzeas). Wetlands Ecol. Manage. 2012, 20, 461–475. 10.1007/s11273-012-9268-0.

[ref4] McClain M. E.; Naiman R. J. Andean Influences on the Biogeochemistry and Ecology of the Amazon River. BioScience 2008, 58, 325–338. 10.1641/b580408.

[ref5] Gonsior M.; Valle J.; Schmitt-Kopplin P.; Hertkorn N.; Bastviken D.; Luek J.; Harir M.; Bastos W.; Enrich-Prast A. Chemodiversity of dissolved organic matter in the Amazon Basin. Biogeosciences 2016, 13, 4279–4290. 10.5194/bg-13-4279-2016.

[ref6] Amon R. M. W.; Benner R. Photochemical and microbial consumption of dissolved organic carbon and dissolved oxygen in the Amazon River system. Geochim. Cosmochim. Acta 1996, 60, 1783–1792. 10.1016/0016-7037(96)00055-5.

[ref7] Ríos-Villamizar E. A.; Adeney J. M.; Piedade M. T. F.; Junk W. J. New insights on the classification of major Amazonian river water types. Sustain. Water Resour. Manag. 2020, 6, 83 10.1007/s40899-020-00440-5.

[ref8] Subdiaga E.; Orsetti S.; Haderlein S. B. Effects of Sorption on Redox Properties of Natural Organic Matter. Environ. Sci. Technol. 2019, 53, 14319–14328. 10.1021/acs.est.9b04684. 31742392

[ref9] Duarte R. M.; Menezes A. C. L.; da Silveira Rodrigues L.; de Almeida-Val V. M. F.; Val A. L. Copper sensitivity of wild ornamental fish of the Amazon. Environ. Ecotoxicol. Environ. Saf. 2009, 72, 693–698. 10.1016/j.ecoenv.2008.10.003. 19058850

[ref10] Simon C.; Osterholz H.; Koschinsky A.; Dittmar T. Riverine mixing at the molecular scale – An ultrahigh-resolution mass spectrometry study on dissolved organic matter and selected metals in the Amazon confluence zone (Manaus, Brazil). Org. Geochem. 2019, 129, 45–62. 10.1016/j.orggeochem.2019.01.013.

[ref11] Li S.; Harir M.; Schmitt-Kopplin P.; Gonsior M.; Enrich-Prast A.; Bastviken D.; Valle J.; Machado-Silva F.; Hertkorn N. Comprehensive assessment of dissolved organic matter processing in the Amazon River and its major tributaries revealed by positive and negative electrospray mass spectrometry and NMR spectroscopy. Sci. Total Environ. 2023, 857, 159620 10.1016/j.scitotenv.2022.159620. 36280052

[ref12] Farjalla V. F.; et al. Are the mixing zones between aquatic ecosystems hot spots of bacterial production in the Amazon River system?. Hydrobiologia 2014, 728, 153–165. 10.1007/s10750-014-1814-8.

[ref13] McClain M. E.; Boyer E. W.; Dent C. L.; Gergel S. E.; Grimm N. B.; Groffman P. M.; Hart S. C.; Harvey J. W.; Johnston C. A.; Mayorga E.; et al. Biogeochemical hot spots and hot moments at the interface of terrestrial and aquatic ecosystems. Ecosystems 2003, 6, 301–312. 10.1007/s10021-003-0161-9.

[ref14] Seidel M.; Dittmar T.; Ward N. D.; Krusche A. V.; Richey J. E.; Yager P. L.; Medeiros P. M. Seasonal and spatial variability of dissolved organic matter composition in the lower Amazon River. Biogeochemistry 2016, 131, 281–302. 10.1007/s10533-016-0279-4.

[ref15] Hertkorn N.; Harir M.; Cawley K. M.; Schmitt-Kopplin P.; Jaffé R. Molecular characterization of dissolved organic matter from subtropical wetlands: a comparative study through the analysis of optical properties, NMR and FTICR/MS. Biogeosciences 2016, 13, 2257–2277. 10.5194/bg-13-2257-2016.

[ref16] Hertkorn N.; Frommberger M.; Witt M.; Koch B. P.; Schmitt-Kopplin P.; Perdue E. M. Natural Organic Matter and the Event Horizon of Mass Spectrometry. Anal. Chem. 2008, 80, 8908–8919. 10.1021/ac800464g. 19551926

[ref17] Drake T. W.; Hemingway J. D.; Kurek M. R.; Peucker-Ehrenbrink B.; Brown K. A.; Holmes R. M.; Galy V.; Moura J. M.; Mitsuya M.; Wassenaar L. I.; et al. The Pulse of the Amazon: Fluxes of Dissolved Organic Carbon, Nutrients, and Ions From the World’s Largest River. Global Biogeochem. Cycles 2021, 35, e2020GB006895 10.1029/2020gb006895.

[ref18] Santos-Junior C. D.; Sarmento H.; de Miranda F. P.; Henrique-Silva F.; Logares R. Uncovering the genomic potential of the Amazon River microbiome to degrade rainforest organic matter. Microbiome 2020, 8, 151 10.1186/s40168-020-00930-w. 33126925 PMC7597016

[ref19] Lundeen R. A.; Janssen E. M. L.; Chu C.; Mcneill K. Environmental Photochemistry of Amino Acids, Peptides and Proteins. Chimia 2014, 68, 812–817. 10.2533/chimia.2014.812. 26508490

[ref20] Hertkorn N.; Benner R.; Frommberger M.; Schmitt-Kopplin P.; Witt M.; Kaiser K.; Kettrup A.; Hedges J. I. Characterization of a major refractory component of marine dissolved organic matter. Geochim. Cosmochim. Acta 2006, 70, 2990–3010. 10.1016/j.gca.2006.03.021.

[ref21] Schmitt-Kopplin P.; Hertkorn N.; Schulten H. R.; Kettrup A. Structural Changes in a Dissolved Soil Humic Acid during Photochemical Degradation Processes under O_2_ and N_2_ Atmosphere. Environ. Sci. Technol. 1998, 32, 2531–2541. 10.1021/es970636z.

[ref22] Gonsior M.; Hertkorn N.; Conte M. H.; Cooper W. J.; Bastviken D.; Druffel E.; Schmitt-Kopplin P. Photochemical production of polyols arising from significant photo-transformation of dissolved organic matter in the oligotrophic surface ocean. Mar. Chem. 2014, 163, 10–18. 10.1016/j.marchem.2014.04.002.

[ref23] Harir M.; Cawley K. M.; Hertkorn N.; Schmitt-Kopplin P.; Jaffé R. Molecular and spectroscopic changes of peat-derived organic matter following photo exposure: Effects on heteroatom composition of DOM. Sci. Total Environ. 2022, 838, 155790 10.1016/j.scitotenv.2022.155790. 35550890

[ref24] Fudyma J. D.; Chu R. K.; Graf Grachet N.; Stegen J. C.; Tfaily M. M. Coupled Biotic-Abiotic Processes Control Biogeochemical Cycling of Dissolved Organic Matter in the Columbia River Hyporheic Zone. Front. Water 2021, 2, 574962 10.3389/frwa.2020.574692.

[ref25] Huang X.; et al. Transformation of algal-dissolved organic matter via sunlight-induced photochemical and microbial processes: interactions between two processes. Environ. Sci. Pollut. Res. 2023, 30, 52969–52981. 10.1007/s11356-023-26024-2. 36843169

[ref26] Moreira-Turcq P. F.; Seyler P.; Guyot J. L.; Etcheber H. Characteristics of organic matter in the mixing zone of the Rio Negro and Rio Solimões of the Amazon River. Hydrol. Processes 2003, 17, 1393–1404. 10.1002/hyp.1291.

[ref27] Mayorga E.; Aufdenkampe A. K.; Masiello C. A.; Krusche A. V.; Hedges J. I.; Quay P. D.; Richey J. E.; Brown T. A. Young organic matter as a source of carbon dioxide outgassing from Amazonian rivers. Nature 2005, 436, 538–541. 10.1038/nature03880. 16049484

[ref28] Abril G.; Martinez J. M.; Artigas L. F.; Moreira-Turcq P.; Benedetti M. F.; Vidal L.; Meziane T.; Kim J. H.; Bernardes M. C.; Savoye N.; et al. Amazon River carbon dioxide outgassing fuelled by wetlands. Nature 2014, 505, 395–398. 10.1038/nature12797. 24336199

[ref29] Ward N. D.; Sawakuchi H. O.; Neu V.; Less D. F. S.; Valerio A. M.; Cunha A. C.; Kampel M.; Bianchi T. S.; Krusche A. V.; Richey J. E.; et al. Velocity-amplified microbial respiration rates in the lower Amazon River. Limnol. Oceanogr. Lett. 2018, 3, 265–274. 10.1002/lol2.10062.

[ref30] Dittmar T.; Koch B.; Hertkorn N.; Kattner G. A simple and efficient method for the solid-phase extraction of dissolved organic matter (SPE-DOM) from seawater. Limnol. Oceanogr.: Methods 2008, 6, 230–235. 10.4319/lom.2008.6.230.

[ref31] Tziotis D.; Hertkorn N.; Schmitt-Kopplin P. Kendrick-Analogous Network Visualisation of Ion Cyclotron Resonance Fourier Transform Mass Spectra: Improved Options for the Assignment of Elemental Compositions and the Classification of Organic Molecular Complexity. Eur. J. Mass Spectrom. 2011, 17, 415–421. 10.1255/ejms.1135. 22006638

[ref32] Seidel M.; Yager P. L.; Ward N. D.; Carpenter E. J.; Gomes H. R.; Krusche A. V.; Richey J. E.; Dittmar T.; Medeiros P. M. Molecular-level changes of dissolved organic matter along the Amazon River-to-ocean continuum. Mar. Chem. 2015, 177, 218–231. 10.1016/j.marchem.2015.06.019.

[ref33] Santoro A. L.; Bastviken D.; Gudasz C.; Tranvik L.; Enrich-Prast A. Dark carbon fixation: an important process in lake sediments. PLoS One 2013, 8, e65813 10.1371/journal.pone.0065813. 23776549 PMC3679121

[ref34] Hertkorn N.; Harir M.; Koch B. P.; Michalke B.; Schmitt-Kopplin P. High-field NMR spectroscopy and FTICR mass spectrometry: powerful discovery tools for the molecular level characterization of marine dissolved organic matter. Biogeosciences 2013, 10, 1583–1624. 10.5194/bg-10-1583-2013.

[ref35] Hsu C. S.; Qian K.; Chen Y. C. An innovative approach to data analysis in hydrocarbon characterization by on-line liquid chromatography-mass spectrometry. Anal. Chim. Acta 1992, 264, 79–89. 10.1016/0003-2670(92)85299-l.

[ref36] Valle J.; Harir M.; Gonsior M.; Enrich-Prast A.; Schmitt-Kopplin P.; Bastviken D.; Hertkorn N. Molecular differences between water column and sediment pore water SPE-DOM in ten Swedish boreal lakes. Water Res. 2020, 170, 115320 10.1016/j.watres.2019.115320. 31837638

[ref37] Koch B. P.; Dittmar T. From mass to structure: an aromaticity index for high-resolution mass data of natural organic matter. Rapid Commun. Mass Spectrom. 2006, 20, 926–932. 10.1002/rcm.7433.

[ref38] Lechtenfeld O. J.; Hertkorn N.; Shen Y.; Witt M.; Benner R. Marine sequestration of carbon in bacterial metabolites. Nat. Commun. 2015, 6, 6711 10.1038/ncomms7711. 25826720

[ref39] Bianchi T. S.; Ward N. D. Editorial: The Role of Priming in Terrestrial and Aquatic Ecosystems. Front. Earth Sci. 2019, 7, 321 10.3389/feart.2019.00321.

[ref40] Ward N. D.; Sawakuchi H. O.; Richey J. E.; Keil R. G.; Bianchi T. S. Enhanced Aquatic Respiration Associated With Mixing of Clearwater Tributary and Turbid Amazon River Waters. Front. Earth Sci. 2019, 7, 101 10.3389/feart.2019.00101.

[ref41] Benner R.; Opsahl S.; Chin-Leo G.; Richey J. E.; Forsberg B. R. Bacterial carbon metabolism in the Amazon River system. Limnol. Oceanogr. 1995, 40, 1262–1270. 10.4319/lo.1995.40.7.1262.

[ref42] Vidal L. O.; Abril G.; Artigas L. F.; Melo M. L.; Bernardes M. C.; Lobão L. M.; Reis M. C.; Moreira-Turcq P.; Benedetti M.; Tornisielo V. L.; et al. Hydrological pulse regulating the bacterial heterotrophic metabolism between Amazonian mainstems and floodplain lakes. Front. Microbiol. 2015, 6, 1054 10.3389/fmicb.2015.01054. 26483776 PMC4588699

[ref43] Wissmar R.; Richey J. E.; Stallard R. F.; Edmond J. M. Plankton metabolism and carbon processes in the Amazon River, its tributaries, and floodplain waters, Peru-Brazil, May-June 1977. Ecology 1981, 62, 1622–1633. 10.2307/1941517.

[ref44] Enrich-Prast A. Chemosynthesis. In Encyclopedia of Inland Waters, 2nd ed.; Mehner T.; Tockner K., Eds.; Elsevier, 2022; pp 118–135, ISBN 9780128220412.

[ref45] Signori C. N.; Valentin J. L.; Pollery R. C. G.; Enrich-Prast A. Temporal variability of dark carbon fixation and bacterial production and their relation with environmental factors in a tropical estuarine system. Front. Aquat. Microbiol. 2018, 41, 1089–1101. 10.1007/s12237-017-0338-7.

[ref46] Signori C. N.; Felizardo J. P. d. S.; Enrich-Prast A. Bacterial production prevails over photo- and chemosynthesis in a eutrophic tropical lagoon. Science 2020, 243, 106889 10.1016/j.ecss.2020.106889.

[ref47] Casamayor E. O.; García-Cantizano J.; Mas J.; Pedrós-Alió C. Primary production in estuarine oxic/anoxic interfaces: contribution of microbial dark CO2 fixation in the Ebro River Salt Wedge Estuary. Mar. Ecol.: Prog. Ser. 2001, 215, 49–56. 10.3354/meps215049.

[ref48] Noguerola I.; Picazo A.; Llirós M.; Camacho A.; Borrego C. M. Diversity of freshwater *Epsilonproteobacteria* and dark inorganic carbon fixation in the sulphidic redoxcline of a meromictic karstic lake. FEMS Microbiol. Ecol. 2015, 91, fiv086 10.1093/femsec/fiv086. 26195601

[ref49] Di Nezio F.; et al. Anoxygenic photo-and chemo-synthesis of phototrophic sulfur bacteria from an alpine meromictic lake. FEMS Microbiol. Ecol. 2021, 97, fiab010 10.1093/femsec/fiab010. 33512460 PMC7947596

[ref50] Overmann J.; Beatty J. T.; Hall K. J. Purple sulfur bacteria control the growth of aerobic heterotrophic bacterioplankton in a meromictic salt lake. Appl. Environ. Microbiol. 1996, 62, 3251–3258. 10.1128/aem.62.9.3251-3258.1996. 16535399 PMC1388937

[ref51] Saini J. S.; Hassler C.; Cable R.; Fourquez M.; Danza F.; Roman S.; Tonolla M.; Storelli N.; Jacquet S.; Zdobnov E. M.; et al. Bacterial, Phytoplankton, and Viral Distributions and Their Biogeochemical Contexts in Meromictic Lake Cadagno Offer Insights into the Proterozoic Ocean Microbial Loop. Mbio 2022, 13, e0005222 10.1128/mbio.00052-22. 35726916 PMC9426590

[ref52] Andersson M. G.; Brion N.; Middelburg J. Comparison of nitrifier activity versus growth in the Scheldt estuary—a turbid, tidal estuary in northern Europe. Aquat. Microb. Ecol. 2006, 42, 149–158. 10.3354/ame042149.

[ref53] Bräuer S. L.; Kranzler K.; Goodson N.; Murphy D.; Simon H. M.; Baptista A. M.; Tebo B. M. Dark carbon fixation in the Columbia River’s estuarine turbidity maxima: molecular characterization of red-type cbbL genes and measurement of DIC uptake rates in response to added electron donors. Estuaries Coasts 2013, 36, 1073–1083. 10.1007/s12237-013-9603-6.

[ref54] Calderón-Paz J. I.; García-Cantizano J.; Vaqué D.; Pedrós-Alió C. Heterotrophic bacterial production in systems of the northern Spanish Mediterranean Region. Int. Ver. Theor. Angew. Limnol., Verh. 1993, 25, 739–742. 10.1080/03680770.1992.11900236.

[ref55] Crump B. C.; Baross J.; Simenstad C. Dominance of particle-attached bacteria in the Columbia River estuary, USA. Aquat. Microb. Ecol. 1998, 14, 7–18. 10.3354/ame014007.

[ref56] Goosen N. K.; van Rijswijk P.; Kromkamp J.; Peene J. Regulation of annual variation in heterotrophic bacterial production in the Schelde estuary (SW Netherlands). Aquat. Microb. Ecol. 1997, 12, 223–232. 10.3354/ame012223.

[ref57] Ertel J. R.; Hedges J. I.; Devol A. H.; Richey J. E.; Ribeiro M. d. N. G. Dissolved humic substances of the Amazon River system. Limnol. Oceanogr. 1986, 31, 739–754. 10.4319/lo.1986.31.4.0739.

[ref58] Johannsson O. E.; Smith D. S.; Sadauskas-Henrique H.; Cimprich G.; Wood C. M.; Val A. L. Photo-oxidation processes, properties of DOC, reactive oxygen species (ROS), and their potential impacts on native biota and carbon cycling in the Rio Negro (Amazonia, Brazil). Hydrobiologia 2017, 789, 7–29. 10.1007/s10750-016-2687-9.

[ref59] Waggoner D. C.; Chen H.; Willoughby A. S.; Hatcher P. G. Formation of black carbon-like and alicyclic aliphatic compounds by hydroxyl radical initiated degradation of lignin. Org. Geochem. 2015, 82, 69–76. 10.1016/j.orggeochem.2015.02.007.

[ref60] Aufdenkampe A. K.; Hedges J. I.; Richey J. E.; Krusche A. V.; Llerena C. A. Sorptive fractionation of dissolved organic nitrogen and amino acids onto fine sediments within the Amazon Basin. Limnol. Oceanogr. 2001, 46, 1921–1935. 10.4319/lo.2001.46.8.1921.

[ref61] Roth V. N.; Lange M.; Simon C.; Hertkorn N.; Bucher S.; Goodall T.; Griffiths R. I.; Mellado-Vázquez P. G.; Mommer L.; Oram N. J.; et al. Persistence of dissolved organic matter explained by molecular changes during its passage through soil. Nat. Geosci. 2019, 12, 755–761. 10.1038/s41561-019-0417-4.

[ref62] Hagerman A. E.; et al. Tannins and lignins. Herbivores: Their Interact. Second. Plant Metab. 1991, 1, 355–388.

[ref63] Schmitt-Kopplin P.; Gelencsér A.; Dabek-Zlotorzynska E.; Kiss G.; Hertkorn N.; Harir M.; Hong Y.; Gebefügi I. Analysis of the unresolved organic fraction in atmospheric aerosols with ultrahigh resolution mass spectrometry and nuclear magnetic resonance spectroscopy: Organosulfates as photochemical smog constituents. Anal. Chem. 2010, 82, 8017–8026. 10.1021/ac101444r. 20879800

[ref64] Aucour A. M.; Tao F. X.; Moreira-Turcq P.; Seyler P.; Sheppard S.; Benedetti M. The Amazon River: behaviour of metals (Fe, Al, Mn) and dissolved organic matter in the initial mixing at the Rio Negro/Solimões confluence. Chem. Geol. 2003, 197, 271–285. 10.1016/s0009-2541(02)00398-4.

[ref65] Pérez M. A.; Moreira-Turcq P.; Gallard H.; Allard T.; Benedetti M. F. Dissolved organic matter dynamic in the Amazon basin: sorption by mineral surfaces. Chem. Geol. 2011, 286, 158–168. 10.1016/j.chemgeo.2011.05.004.

[ref66] Subdiaga E.; Harir M.; Orsetti S.; Hertkorn N.; Schmitt-Kopplin P.; Haderlein S. B. Preferential Sorption of Tannins at Aluminum Oxide Affects the Electron Exchange Capacities of Dissolved and Sorbed Humic Acid Fractions. Environ. Sci. Technol. 2020, 54, 1837–1847. 10.1021/acs.est.9b04733. 31894976

[ref67] Merschel G.; Bau M.; Dantas E. L. Contrasting impact of organic and inorganic nanoparticles and colloids on the behavior of particle-reactive elements in tropical estuaries: an experimental study. Geochim. Cosmochim. Acta 2017, 197, 1–13. 10.1016/j.gca.2016.09.041.

[ref68] Medeiros P. M.; Seidel M.; Ward N. D.; Carpenter E. J.; Gomes H. R.; Niggemann J.; Krusche A. V.; Richey J. E.; Yager P. L.; Dittmar T. Fate of the Amazon River dissolved organic matter in the tropical Atlantic Ocean. Global Biogeochem. Cycles 2015, 29, 677–690. 10.1002/2015gb005115.

[ref69] Miao Y.; Lv J.; Huang H.; Cao D.; Zhang S. Molecular characterization of root exudates using Fourier transform ion cyclotron resonance mass spectrometry. J. Environ. Sci. 2020, 98, 22–30. 10.1016/j.jes.2020.05.011. 33097154

[ref70] Doherty M.; Yager P. L.; Moran M. A.; Coles V. J.; Fortunato C. S.; Krusche A. V.; Medeiros P. M.; Payet J. P.; Richey J. E.; Satinsky B. M.; et al. Bacterial Biogeography across the Amazon River-Ocean Continuum. Front. Microbiol. 2017, 8, 882 10.3389/fmicb.2017.00882. 28588561 PMC5440517

[ref71] Benner R.; Amon R. M. The size-reactivity continuum of major bioelements in the ocean. Ann. Rev. Mar. Sci. 2015, 7, 185–205. 10.1146/annurev-marine-010213-135126. 25062478

[ref72] Bertassoli D. J.; Sawakuchi A. O.; Sawakuchi H. O.; Pupim F. N.; Hartmann G. A.; McGlue M. M.; Chiessi C. M.; Zabel M.; Schefuß E.; Pereira T. S.; et al. The Fate of Carbon in Sediments of the Xingu and Tapajós Clearwater Rivers, Eastern Amazon. Front. Mar. Sci. 2017, 4, 44 10.3389/fmars.2017.00044.

[ref73] Melo M. L.; Kothawala D. N.; Bertilsson S.; Amaral J. H.; Forsberg B.; Sarmento H. Linking dissolved organic matter composition and bacterioplankton communities in an Amazon floodplain system. Limnol. Oceanogr. 2019, 65, 63–76. 10.1002/lno.11250.

[ref74] Ward N. D.; Bianchi T. S.; Sawakuchi H. O.; Gagne-Maynard W.; Cunha A. C.; Brito D. C.; Neu V.; de Matos Valerio A.; da Silva R.; Krusche A. V.; et al. The reactivity of plant-derived organic matter and the potential importance of priming effects along the lower Amazon River. J. Geophys. Res. 2016, 121, 1522–1539. 10.1002/2016jg003342.

[ref75] Hawkes J. A.; Hansen C. T.; Goldhammer T.; Bach W.; Dittmar T. Molecular alteration of marine dissolved organic matter under experimental hydrothermal conditions. Geochim. Cosmochim. Acta 2016, 175, 68–85. 10.1016/j.gca.2015.11.025.

[ref76] Raeke J.; Lechtenfeld O. J.; Wagner M.; Herzsprung P.; Reemtsma T. Selectivity of solid phase extraction of freshwater dissolved organic matter and its effect on ultrahigh resolution mass spectra. Environ. Sci.: Processes Impacts 2016, 18, 918–927. 10.1039/c6em00200e. 27363664

[ref77] Li Y.; Harir M.; Uhl J.; Kanawati B.; Lucio M.; Smirnov K. S.; Koch B. P.; Schmitt-Kopplin P.; Hertkorn N. How representative are dissolved organic matter (DOM) extracts ? A comprehensive study of sorbent selectivity for DOM isolation. Water Res. 2017, 116, 316–323. 10.1016/j.watres.2017.03.038. 28359043

[ref78] Kujawinski E. B.; Del Vecchio R.; Blough N. V.; Klein G. C.; Marshall A. G. Probing molecular-level transformations of dissolved organic matter: insights on photochemical degradation and protozoan modification of DOM from electrospray ionization Fourier transform ion cyclotron resonance mass spectrometry. Mar. Chem. 2004, 92, 23–37. 10.1016/j.marchem.2004.06.038.

[ref79] Vannote R. L.; Minshall G. W.; Cummins K. W.; Sedell J. R.; Cushing C. E. The River Continuum Concept. Can. J. Fish. Aquat. Sci. 1980, 37, 130–137. 10.1139/f80-017.

[ref80] Kamjunke N.; Hertkorn N.; Harir M.; Schmitt-Kopplin P.; Griebler C.; Brauns M.; von Tümpling W.; Weitere M.; Herzsprung P. Molecular change of dissolved organic matter and patterns of bacterial activity in a stream along a land-use gradient. Water Res. 2019, 164, 114919 10.1016/j.watres.2019.114919. 31382154

[ref81] Devol A. H.; Hedges J. I. Organic matter and nutrients in the mainstem Amazon River. Biogeochem. Amazon Basin 2001, 15, 275–306. 10.1093/oso/9780195114317.003.0018.

[ref82] Schmidt M. W.; Torn M. S.; Abiven S.; Dittmar T.; Guggenberger G.; Janssens I. A.; Kleber M.; Kögel-Knabner I.; Lehmann J.; Manning D. A. C.; et al. Persistence of soil organic matter as an ecosystem property. Nature 2011, 478, 49–56. 10.1038/nature10386. 21979045

[ref83] Valle J.; Gonsior M.; Harir M.; Enrich-Prast A.; Schmitt-Kopplin P.; Bastviken D.; Conrad R.; Hertkorn N. Extensive processing of sediment pore water dissolved organic matter during anoxic incubation as observed by high-field mass spectrometry (FTICR-MS). Water Res. 2018, 129, 252–263. 10.1016/j.watres.2017.11.015. 29153878

[ref84] Einsiedl F.; Hertkorn N.; Wolf M.; Frommberger M.; Schmitt-Kopplin P.; Koch B. P. Rapid biotic molecular transformation of fulvic acids in a karst aquifer. Geochim. Cosmochim. Acta 2007, 71, 5474–5482. 10.1016/j.gca.2007.09.024.

[ref85] Eadie B.; Jeffrey L.; Sackett W. Some observations on the stable carbon isotope composition of dissolved and particulate organic carbon in the marine environment. Geochim. Cosmochim. Acta 1978, 42, 1265–1269. 10.1016/0016-7037(78)90120-5.

[ref86] Meyers-Schulte K. J.; Hedges J. I. Molecular evidence for a terrestrial component of organic matter dissolved in ocean water. Nature 1986, 321, 61–63. 10.1038/321061a0.

[ref87] Opsahl S.; Benner R. Distribution and cycling of terrigenous dissolved organic matter in the ocean. Nature 1997, 386, 480–482. 10.1038/386480a0.

[ref88] Bianchi T. S. The role of terrestrially derived organic carbon in the coastal ocean: a changing paradigm and the priming effect. Proc. Natl. Acad. Sci. U.S.A. 2011, 108, 19473–19481. 10.1073/pnas.1017982108. 22106254 PMC3241778

[ref89] Jiao N.; Herndl G. J.; Hansell D. A.; Benner R.; Kattner G.; Wilhelm S. W.; Kirchman D. L.; Weinbauer M. G.; Luo T.; Chen F.; Azam F. Microbial production of recalcitrant dissolved organic matter: long-term carbon storage in the global ocean. Nat. Rev. Microbiol. 2010, 8, 593–599. 10.1038/nrmicro2386. 20601964

[ref90] Baltar F.; Herndl G. J. Ideas and perspectives: Is dark carbon fixation relevant for oceanic primary production estimates?. Biogeosciences 2019, 16, 3793–3799. 10.5194/bg-16-3793-2019.

[ref91] Jorcin A.; Nogueira M. G. Temporal and spatial patterns based on sediment and sediment–water interface characteristics along a cascade of reservoirs (Paranapanema River, south-east Brazil). Lakes Reserv.: Res. Manag. 2005, 10, 1–12. 10.1111/j.1440-1770.2005.00254.x.

