# Supplementary material for: Distinct Non-conservative Behavior of Dissolved Organic Matter after Mixing Solimões/Negro and Amazon/Tapajós River Waters
Source: ACS ES T Water. 2023 Jun 12;3(8):2083–95. doi: 10.1021/acsestwater.2c00621 (PMC10425957; doi:10.1021/acsestwater.2c00621)
Supplement: Supplementary file 1 — ew2c00621_si_001.pdf [file ew2c00621_si_001.pdf]

Supporting Information for

Distinct non-conservative behavior of dissolved organic matter after mixing Solimões/Negro and Amazon/Tapajós River waters

AUTHOR NAMES

Siyu Li[a], Mourad Harir[a,b], Philippe Schmitt-Kopplin[a,b], Fausto Machado-Silva[c,d], Michael Gonsior[e], David Bastviken[f], Alex Enrich-Prast[f,g,h, i], Juliana Valle[a], Norbert Hertkorn[a,f]*

AUTHOR ADDRESS

[a] Research Unit Analytical Biogeochemistry, Helmholtz Munich, Ingolstaedter Landstrasse 1, 85764, Neuherberg, Germany.

[b] Chair of Analytical Food Chemistry, Technische Universität München; Alte Akademie 10, 85354, Freising-Weihenstephan, Germany.

[c] Program in Geosciences – Environmental Geochemistry, Chemistry Institute, Fluminense Federal University, 24020-141, Niteroi, Brazil.

[d] Department of Environmental Sciences, University of Toledo, Toledo, OH, 43606, USA.

[e] University of Maryland Center for Environmental Science, Chesapeake Biological Laboratory; Solomons, Maryland 20688, USA.

[f] Department of Thematic Studies – Environmental Change, Linköping University; SE-581 83 Linköping, Sweden.

[g] Biogas Solutions Research Center (BSRC), Linköping University; SE-581 83 Linköping, Sweden.

[h] Institute of Marine Science, Federal University of São Paulo, Santos, Brazil.

[i] Multiuser Unit of Environmental Analysis, University Federal of Rio de Janeiro, Rio de Janeiro, Brazil.

Number of pages: 34

Number of Tables: 11

Number of Figures: 14

TABLE OF CONTENTS

Details of Material and Methods **5**
1. Sample collection.. **5**
2. FT-ICR MS analysis.. **6**
3. ^{1}H NMR analysis.. **6**
4. Water analysis.. **7**
5. Microbial analysis.. **7**
6. Statistical Analysis.. **8**
Table S1. Description of sample information. **8**
Table S2. Description of samples in mixing and incubation experiments.............................. **9**
Figure S1. (A) ESI[-] and (B) ESI[+] FT-ICR mass spectra of SPE-DOM in unmixed and mixed S+N water pre- and post- incubation. The FT-ICR mass spectra are exemplified with samples *S*, *N*, FSFN, S50N50_30min, S50N50_1d, FSSN_B_1d, FNSS_B_1d, and S50N50_5d_1. The ESI[-] and ESI [+] FT-ICR mass spectra in (a1) and (b1) show the regular shaped signal distribution over a wide mass range (m/z 150-950), as well as distinct signatures at nominal neutral mass 400. Respective assignments of molecular compositions are provided for CHO (blue) and CHNO (orange) molecules. **10**
Table S3. ESI[-] FT-ICR MS derived counts of mass peaks and intensity-weighted average bulk parameters for all assigned molecular compositions present in SPE-DOM in unmixed and mixed S+N water pre- and post- incubation. FT-ICR MS derived bulk parameters comprise percentages of counts of CHO, CHNO, CHOS, and CHNOS molecular classes, computed experimental *m/z*, DBE/C, H/C, N/C, and S/C ratio. **11**
Table S4. ESI[+] FT-ICR MS derived counts of mass peaks and intensity-weighted average bulk parameters for all assigned molecular compositions present in SPE-DOM in unmixed and mixed S+N water pre- and post- incubation. FT-ICR MS derived bulk parameters comprise percentages of counts of CHO, CHNO, CHOS, and CHNOS molecular classes, computed experimental *m/z*, DBE/C, H/C, N/C, and S/C ratio. **12**
Figure S2. Loading vectors in PCA of SPE-DOM in unmixed and mixed S+N water pre- and post-incubation based on all assigned molecular formulae in (A, B) ESI[±] FT-ICR mass spectra and (C) ^{1}H NMR spectra . The scatter plots of the very PCA see Figure 2. Panels A and B show correlation patterns of PC1 and PC2 loading vectors (p1 and p2) in van Krevelen and mass-edited H/C diagrams, with the color code representing CHO (blue), CHNO (orange), and CHOS (green) molecular classes. The numbers show counts of compounds. The molecular formulae positioned below the purple line in the van Krevelen diagrams have modified aromaticity index (AI_{mod})[15] higher than 0.5. Panel C show correlation patterns of p1 and p2 in ^{1}H NMR space, with fundamental molecular structures indicated. **13**
Table S5. Water parameters in unmixed and mixed S+N and A+T waters pre- and post- incubation. **14**
Figure S3. HCA of assigned SPE-DOM molecular formulae in part of the unmixed and mixed S+N water pre- and post- incubation. Van Krevelen, mass-edited H/C, and KMD/z* diagrams[16] show CHO (blue), CHNO (orange), and CHOS (green) molecular classes that were relatively more abundant in clusters a1/a2/a3, b1/b2/b3, respectively. Bubble areas represent the normalized intensities of SPE-DOM in each cluster. The pie plots depict percentages of the counts of different molecular classes. The numbers show counts of compounds. The molecular formulae positioned

below the purple line in the van Krevelen diagrams have modified aromaticity index (AI_{mod})[15] higher than 0.5. .... **15**

Figure S4. Common and unique molecular signatures in SPE-DOM in the unmixed and mixed S+N water pre- and post- incubation. The numbers show counts of compounds. Van Krevelen and mass-edited H/C diagrams show CHO (blue), CHNO (orange), CHOS (green), and CHNOS (red) m/z ions that were shared before and after incubation (a1 and b1); unique in original water but were not in incubated water (a2 and b2). Not any single compound unique to incubated water did not present in original water. Bubble areas represent the normalized intensity of DOM. The pie plots depict percentages of the counts of different molecular classes. Molecular composition positioned below the purple line in the van Krevelen diagrams shows the compounds with a modified aromaticity index (AI_{mod}) higher than 0.5[15]. Panel C shows the normalized intensity of molecular signatures that were degraded during the incubation process (a2 and b2) in DOM of the Amazon mainstem. **16**

Figure S5. ^{1}H NMR spectra of SPE-DOM in the unmixed and mixed S+N water pre- and post-incubation. The ^{1}H NMR spectra are exemplified with samples *S*, *N*, FSFN, S50N50_30min, S50N50_1d, FSSN_B_1d, FNSS_B_1d, and S50N50_5d_1. ^{1}H NMR spectra are area normalized (800 MHz, CD_3OD; δH = 0.5-10.0 ppm with the exclusion of residual water and methanol NMR resonances). The ^{1}H NMR spectra referred to average abundance are edited by each normalized spectra subtract the average spectra (C). **17**

Table S6. ^{1}H NMR section integrals (percent of non-exchangeable protons, 800 MHz; CD_3OD, exclusion of residual water, and methanol)) and key substructures of SPE-DOM in the unmixed and mixed S+N water pre- and post- incubation. The fundamental substructures include aromatics C_{ar}**H**, δ_H ~ 7.0-10.0 ppm; olefins δ_H ~ 5.3-7.0 ppm; oxygenated aliphatic units (OC**H**) and "carbohydrate-like" and methoxy OC**H**$_3$ units δ_H ~ 3.2-4.9 ppm; branched aliphatic units (C**H**$_2$)$_n$, "acetate-analogue" and CRAM δ_H ~ 1.9-3.2 ppm; functionalized aliphatics δ_H ~ 1.35-1.9 ppm; polyethylene group δ_H ~ 1.25-1.35 ppm, OCC**H** units; pure aliphatics δ_H ~ 0.5-1.25 ppm, CCC**H** units. **18**

Figure S6. Difference ^{1}H NMR spectra (800 MHz, CD_3OD) of Solimões and Negro SPE-DOM according to status of incubation. (A) Solimões minus Negro, with Solimões (S), Negro (N), and Solimões minus Negro (S-N). (B) difference ^{1}H NMR spectrum after 30 minutes of incubation, with S, N, averaged Solimões and Negro ((S+N)/2), S50N50_30min, and (S+N)/2 minus S50N50_30min. (C) difference ^{1}H NMR spectrum after one day of incubation, with S, N, (S+N)/2, S50N50_1d, and (S+N)/2 minus S50N50_1d. (D) difference ^{1}H NMR spectrum after five days of incubation, with S, N, (S+N)/2, S50N50_5d, and (S+N)/2 minus S50N50_5d. (E) difference ^{1}H NMR spectrum of one day minus five days of incubation, with S50N50_1d, S50N50_5d, and (S50N50_1d minus S50N50_5d). **19**

Figure S7. Difference ^{1}H NMR spectra (800 MHz, CD_3OD) of Solimões and Negro SPE-DOM according to status of incubation, section of unsaturated C_{sp2}H protons δ_H ~ 5.5-10 ppm in Figure S6. (A) Solimões minus Negro, with Solimões (S), Negro (N), and Solimões minus Negro (S-N). (B) difference after 30 minutes of incubation, with S, N, averaged Solimões and Negro ((S+N)/2), S50N50_30min, and (S+N)/2 minus S50N50_30min. (C) difference after one day of incubation, with S, N, (S+N)/2, S50N50_1d, and (S+N)/2 minus S50N50_1d. (D) difference ^{1}H NMR spectrum after five days of incubation, with S, N, (S+N)/2, S50N50_5d, and (S+N)/2 minus S50N50_5d. (E) difference ^{1}H NMR spectrum one day minus five days of incubation, with S50N50_1d, S50N50_5d, and (S50N50_1d minus S50N50_5d). **20**

Figure S8. Difference ^{1}H NMR spectra (800 MHz, CD_3OD) of Solimões and Negro river waters, entire section $\delta_H \sim 0.5$-10 ppm), and proximate Amazon SPE-DOM samples A1 and A2 (sampling locations see Figure 1 and Table S1). (A) (averaged Solimões and Negro) river minus proximate downstream Amazon River sampling points A1 (orange, dotted line) and A2 (pink, dotted line), with Solimões (S, grey), Negro (N, black), and averaged Solimões and Negro ((S+N)/2, dotted green line). (B) same spectra, section of unsaturated $C_{sp2}H$ protons ($\delta_H \sim 5.5$-10 ppm). ………………………………………………………………………………………………**21**
Figure S9. (A) ESI[-] and (B) ESI[+] FT-ICR mass spectra of SPE-DOM in unmixed and mixed A+T water pre- and post- incubation. The FT-ICR mass spectra are exemplified with samples *A*, *T*, A50T50_1d, and A50T50_5d. The ESI[-] and ESI [+] FT-ICR mass spectra in (a1) and (b1) show the regular shaped signal distribution over a wide mass range (m/z 150-950), as well as distinct signatures at nominal neutral mass 400. Respective assignments of molecular compositions are provided for CHO (blue) and CHNO (orange) molecules. ……………………**22**
Table S7. ESI[-] FT-ICR MS derived counts of mass peaks and intensity-weighted average bulk parameters for all assigned molecular compositions present in unmixed and mixed A+T water pre- and post- incubation. FT-ICR MS derived bulk parameters comprise percentages of counts of CHO, CHNO, CHOS, and CHNOS molecular classes, computed experimental *m/z*, DBE/C, H/C, N/C, and S/C ratio. ………………………………………………………………………………**23**
Table S8. ESI[+] FT-ICR MS derived counts of mass peaks and intensity-weighted average bulk parameters for all assigned molecular compositions present in unmixed and mixed A+T water pre- and post- incubation. FT-ICR MS derived bulk parameters comprise percentages of counts of CHO, CHNO, CHOS, and CHNOS molecular classes, computed experimental *m/z*, DBE/C, H/C, N/C, and S/C ratios. ………………………………………………………………………………**24**
Figure S10. HCA of assigned SPE-DOM molecular formulae in the unmixed and mixed A+T water pre- and post-incubation. Van Krevelen, mass-edited H/C, and KMD/z* diagrams[16] show CHO (blue), CHNO (orange), and CHOS (green) molecular classes that were relatively more abundant in clusters a1/a2, b1/b2, respectively. The pie plots depict percentages of the counts of different molecular classes. The numbers show counts of compounds. The molecular formulae positioned below the purple line in the van Krevelen diagrams have modified aromaticity index (AI_{mod})[15] higher than 0.5. ……………………………………………………………………**25**
Figure S11. Common and unique molecular signatures in unmixed and mixed A+T water pre- and post- incubation. The numbers show counts of compounds. Van Krevelen and mass-edited H/C diagrams show CHO (blue), CHNO (orange), CHOS (green), and CHNOS (red) *m/z* ions that were shared before and after incubation (a1 and b1); unique in original water but were not in incubated water (a2 and b2); unique in incubated water but were not in original water (a3 and b3); unique in one-day incubated water (1d) but were not present in five-day incubated water (5d) (a4 and b4). There was no compound unique to 5d and not present 1d. The pie plots depict percentages of the counts of different molecular classes. The numbers show counts of compounds. The molecular formulae positioned below the purple line in the van Krevelen diagrams have modified aromaticity index (AI_{mod})[15] higher than 0.5. ……………………………………………………………………**26**
Figure S12. ^{1}H NMR spectra of SPE-DOM in unmixed and mixed A+T water pre- and post-incubation. The ^{1}H NMR spectra are exemplified with sample *A*, *T*, A50T50_1d, and A50T50_5d. ^{1}H NMR spectra are entire region normalized (800 MHz, CD_3OD; δ_H = 0.5-10.0 ppm with exclusion of residual water and methanol NMR resonances). The ^{1}H NMR spectra referred to average abundance in the four DOM are edited by each normalized spectra subtract the average spectra of the four DOM (C). ……………………………………………………………………**27**

Table S9. ^{1}H NMR section integrals (percent of non-exchangeable protons, 800 MHz; CD_3OD, exclusion of residual water, and methanol)) and key substructures of SPE-DOM in unmixed and mixed A+T water pre- and post- incubation. The fundamental substructures include aromatics C_{ar}**H**, δ_H ~ 7.0-10.0 ppm; olefins δ_H ~ 5.3-7.0 ppm; oxygenated aliphatic units (OC**H**) and "carbohydrate-like" and methoxy OC**H**$_3$ units δ_H ~ 3.2-4.9 ppm; branched aliphatic units (C**H**$_2$)$_n$, "acetate-analogue" and CRAM δ_H ~ 1.9-3.2 ppm; functionalized aliphatics δ_H ~ 1.35-1.9 ppm; polyethylene group δ_H ~ 1.25-1.35 ppm, OCC**H** units; pure aliphatics δ_H ~ 0.5-1.25 ppm, CCC**H** units. ……………………………………………………………………………………**28**
Figure S13. Difference ^{1}H NMR spectra (800 MHz, CD_3OD) of Amazon and Tapajós river SPE-DOM according to status of incubation (full vertical expansion cf. Figure S14). (A) Amazon minus Tapajós river, with Amazon (A), Tapajós (T), and Amazon minus Tapajós (A-T). (B) difference ^{1}H NMR spectrum after one day of incubation, with A, T, averaged Amazon and Tapajós ((A+T)/2), A50T50_1d, and (A+T)/2 minus A50T50_1d. (C) difference ^{1}H NMR spectrum after five days of incubation, with A, T, (A+T)/2, A50T50_5d, and (A+T)/2 minus A50T50_5d. (D) difference ^{1}H NMR spectrum one day minus five days of incubation, with A50T50_1d, A50T50_5d, and (A50T50_1d minus A50T50_5d). ……………………………………………………**29**
Figure S14. Difference ^{1}H NMR spectra (800 MHz, CD_3OD) of Amazon and Tapajós river SPE-DOM according to status of incubation. (A) Amazon minus Tapajós river, with Amazon (A), Tapajós (T), and Amazon minus Tapajós (A-T). (B) difference ^{1}H NMR spectrum after one day of incubation, with A, T, averaged Amazon and Tapajós ((A+T)/2), A50T50_1d, and (A+T)/2 minus A50T50_1d. (C) difference ^{1}H NMR spectrum after five days of incubation, with A, T, (A+T)/2, A50T50_5d, and (A+T)/2 minus A50T50_5d. (D) difference ^{1}H NMR spectrum one day minus five days of incubation, with A50T50_1d, A50T50_5d, and (A50T50_1d minus A50T50_5d). ……………………………………………………………………………**30**
Table S10. HBP and DCF in unmixed and mixed S+N and A+T waters pre- and post- incubation. SD, Standard Deviation; n, number of replicates; N.A., not available. …………………………**31**
Table S11. Ranges of heterotrophic bacterial production (HBP) and dark carbon fixation (DCF) in this study and in the Amazon River other aquatic systems. ……………………………………**32**

Details of Material and Methods

1. Sample collection

The Amazon River main stem originate in Andean mountains springs and for most of its flow path meanders across the lowland of Amazon tropical rainforest until its mouth in Atlantic Ocean, while collecting major tributaries on its way. The main stem of the Amazon River is named Ucayali while flowing in Peru and when it crosses Brazil's boundary it changes the name to Solimões. Downstream of the confluence with the Negro River in Manaus (Amazonas state, Brazil) it changes the name again to Amazon and keeps this name until its mouth on Atlantic Ocean in Brazil's coast. There are many large tributaries with different water characteristics. Three fundamental types of water were already described by Alfred Russel Wallace in 1853 in his trip to the Amazon basin, and these were called as white, clear and blackwaters[1]. The color varies depending on whether dissolved organic matter (black water) or suspended sediment (white water) is predominant[2]. The clear water rivers are transparent and supply a high phytoplankton production[3]. Both white and clear waters have more aquatic plants and floating meadows than black waters. In contrast, the black water Negro River is nearly devoid of such vegetation. Solimões contributed the most carbon to the Amazon River, about 500 kg C s^{-1} during the sampling high water period[2]. Madeira is the largest and Negro is the second largest tributary in Amazon

watershed. These two rivers together contribute more than 30% of discharge of the Amazon River main stem. Madeira is a primary tributary of Amazon in terms of sediment input. Sampling location coordinates and information of the water samples is described in Tables S1, S2.

2. FT-ICR MS analysis

Negative and positive electrospray ionization (ESI[±]) Fourier transform ion cyclotron resonance mass spectra (FT-ICR MS) were acquired using a 12T Bruker Solarix mass spectrometer (Bruker Daltonics, Bremen, Germany) and an Apollo II electrospray ionization (ESI) source[4]. Nebulizer gas pressure, drying gas pressure and the source heater temperature were 138 kPa, 103 kPa and 200 °C, respectively. The spectra were acquired with a time domain of 4 MW. For each sample, 500 broadband scans were accumulated in ESI[-] FT-ICR mass spectra, while 300 broadband scans were accumulated in ESI[+] FT-ICR mass spectra. All spectra were first externally calibrated on clusters of arginine in MeOH (0.57 mmol/L) and internally calibrated using appropriate reference mass lists of common natural organic matter molecules, reaching accuracy values lower than 500 ppb. Data processing was done using Compass Data Analysis 5.0 (Bruker, Bremen, Germany) and formula assignment by an in-house made software (NetCalc)[5]. The molecular formula assignments were based on the following elements: $^{1}H_{0\text{-}200}$, $^{12}C_{0\text{-}100}$, $^{16}O_{0\text{-}80}$, $^{32}S_{0\text{-}3}$, $^{14}N_{0\text{-}3}$ as well as the $^{13}C_{0\text{-}1}$ and $^{34}S_{0\text{-}1}$ isotopomers. The generated formulae were validated by setting sensible chemical constraints (N rule, $O/C < 1$, $H/C < 2n+2$ (C_nH_{2n+2})). Restriction on nitrogen atoms≤ 4 and sulfur atoms≤ 2 were applied based on previous studies[6,7]. Final elemental formulae were generated and categorized into groups containing CHO, CHNO, CHOS, and CHNOS molecular series, which were used to reconstruct the group-selective mass spectra. The intensity of mass peaks was normalized (total mass peak amplitude of assigned mass peaks = 100% for single sample). The average H/C, O/C, N/C, S/C atomic ratios, DBE/C (Double bond equivalent per carbon), mass-to-charge ratios (*m/z*) were computed from the intensity-weighted average of molecular formulae[4].

3. ^{1}H NMR analysis

All ^{1}H NMR spectra were acquired with a Bruker Advance III NMR spectrometer operating at 800.35 MHz (B_0 = 18.8 Tesla) at 283 K from redissolved dried DOM in CD_3OD (99.95% ^{2}H; Merck) with Bruker standard pulse sequences using 3.0 mm Bruker MATCH tubes. The reference ^{1}H NMR chemical shift of HD_2COD was 3.3 ppm. ^{1}H NMR spectra were recorded under solvent suppression with presaturation and 1 ms spin-lock (noesypr1d), 5 s acquisition time, 5 s relaxation delay (d1), typically 1024 scans and 1 Hz exponential line broadening. ^{1}H NMR section integrals were obtained by using the software AMIX at 0.01 ppm resolution, with exclusions of HDO and HD_2COD NMR resonances.

Difference ^{1}H NMR spectra used full data point resolution (1.5×10^{-4} ppm) and were computed from area normalized NMR spectra (δ_H: 0.5-10.0 ppm), with exclusion of methanol (δ_H: 3.2-3.4 ppm) and water section chemical shifts (δ_H: 4.8-5.5 ppm) by Bruker AMIX software; data tables were imported into EXCEL tables, and difference NMR spectra were computed in EXCEL and exported to Adobe Illustrator. Vertical axes denote fraction of NMR resonance amplitude with respect to total ^{1}H NMR integral (100%).

4. Water analysis

Water conductivity (Cond.), temperature (Temp.) and dissolved oxygen (DO) were measured in situ with portable instruments (Hanna Instruments, Metrohm electrode and PRO-ODO YSI). For the inorganic dissolved nutrients, the water was sampled with polypropylene bottles, filtered in silica filters (GF/F, Whatman) and placed in amber bottles conditioned in freezer to be analyzed in the laboratory. Ammonium (NH_4^+) concentrations were determined by colorimetric analysis with flux injection (FIA, FiaStar 5000). Nitrate (NO_3^-) and nitrite (NO_2^-) were measured using

nitrate reduction and phosphate (PO_4^{3-}) using molybdenum analytical method[8]. The dissolved organic carbon (DOC) was determined by persulfate digestion analyzed using high-temperature catalytic oxidation method (Sievers InnovOx analyzer, GE)[9]. The total organic carbon (TOC) used the same analytical method as DOC without water filtration.

5. Microbial analysis

HBP was estimated by the measurement of protein synthesis rates using ^{3}H-leucine[10], previously tested in the Amazon River mixing zones[11]. We added 150 µL of Leucine[^{3}H] (at the final concentration of 20 nM with the specific radioactivity of 10 Ci mmol^{-1}, Perkin Elmer) to 1.5 mL of river water in five replicates and two controls. We used formalin at a final concentration of 3.7% to stop the incubation after 45 minutes and to fix two controls before the leucine addition. In the laboratory, the bacterial protein was extracted using cold trichloroacetic acid (TCA) and ethanol protocol[12]. The precipitated protein pellet was re-suspended in the scintillation liquid (OptiphaseHiSafe, PerkinElmer) and leucine incorporation was calculated as the net decay per minute based in controls and the concentration factor calculated based on the leucine added[12]. Then, we calculate HBP converting leucine incorporation in carbon assimilation using the molecular weight of leucine, the proportion of leucine in total protein, the ratio of cellular carbon to protein, and the intracellular isotope dilution of Leucine[12].

DCF is a proxy of chemosynthesis that was estimated by the incorporation of dissolved inorganic carbon (^{14}C-DIC) as done for radiotracer photosynthesis measurements, but under dark conditions[13]. The incubations were performed by the addition of 10 µL of $NaH^{14}CO_3$ (Perkin Elmer, the specific activity of 52.5 mCi mmol^{-1}) in 50 mL of river water in amber bottles at each sampling site using five replicates and two controls. The incubations were stopped after 4 hours by adding formaldehyde to the final concentration of 3.7 %. In the laboratory, samples extracted using filtration in glass fiber filters (GF/F, Whatman) and cellulose acetate filters (0.2 µm pore, Sartorius Stedim Biotech), washed twice and placed with hydrochloric acid (HCl) 1 M to remove traces of inorganic carbon. Finally, filters activity were counted using scintillation liquid (OptiphaseHiSafe, PerkinElmer) and scintillation counter 24 h later (Tricarb 2800, Perkin Elmer). We estimated dissolved inorganic carbon (DIC) in the water using the acidified headspace method[14] using 12 mL of water sample into small, sealed glass vials (25 mL) prefilled with 50 µL phosphoric acid (20%), which was empirically tested to lower the pH (~2). In the laboratory, samples were analyzed for CO_2 using gas chromatography (Agilent Technologies, USA, 7890A) with a 1.8 m × 3.175 mm Porapak Q 80/100 column from Supelco, a methanizer converting CO_2 to methane and determination by a flame ionization detector (FID) by manual injection. The CO_2 concentration measured in the headspace is then recalculated to DIC because the lowered pH (~2) drives the DIC into CO_2 with partitioning between the water and gas phases according to Henry's law. Then, DCF the fraction of assimilated ^{14}C was calculated as the sum of both filters times the total DIC levels present. Then, DCF was calculated as the DIC incorporation per time for each sample based on the net decay per minute based on controls and the isotopic discrimination factor[13].

6. Statistical analysis

Principal component analysis (PCA) was performed using Simca-P (version 11.5, UmetricsAB, Umeå, Sweden) to identify the dominant modes of variability in DOM composition of AZ-DOM. FT-ICR Mass spectra were arranged with the samples as observations and the peak areas of the assigned FT-ICR mass peaks as the response variables. ^{1}H NMR spectra were arranged with the samples as observations and the NMR resonances as the response variables (800 MHz ^{1}H NMR, CD_3OD, area-normalized from 0.5-10.0 ppm; 0.01 ppm bucket resolution; with the exclusion of

residual water and methanol NMR resonances). Before multivariate statistics were performed, the response variables were centered and scaled to unit variance. The based weight was computed as 1/sqrt (standard deviation of the response variables).
Hierarchical Cluster Analysis (HCA) was performed using the Hierarchical Clustering Explorer 3.0 (HCE; http://www.cs.umd.edu/hcil/multi-cluster/). Average Linkage (UPGMA) method was used to cluster the dataset and Euclidean distance was used as the similarity/distance measure. Based on the HCA, we used the "profile search" tool from HCE 3.0, choosing a search method (model-based), a distance measure (Pearson's r) and a threshold (0.9).

Table S1. Description of sample information.

Sample	River	Description	Longitude (°W)	Latitude (°S)	Water type
S	Solimões	used for incubation	-60.0957	-3.3136	white
SM	Solimões	Solimões close to SN mixing zone	-59.8949	-3.1647	turbid
N	Negro	used for incubation	-60.0902	-3.1242	black
NaM	Negro	Negro after Manaus	-59.9126	-3.1292	black
MZ	SN mixing zone	SN mixing zone	-59.8958	-3.1301	turbid
A1	Amazon	before Madeira River inflow	-60.1596	-2.7933	turbid
A2	Amazon	before Madeira River inflow	-58.7621	-3.2992	turbid
AM1	Amazon	after Madeira River inflow	-58.5192	-3.1802	turbid
AM2	Amazon	after Madeira River inflow	-58.5513	-3.1492	turbid
Ad1	Amazon	Amazon downstream	-55.7717	-2.0033	turbid
Ad2	Amazon	Amazon downstream	-55.4316	-2.0180	turbid
A	Amazon	used for incubation	-54.7185	-2.4041	turbid
At	Amazon	a small river branch in Amazon after Tapajós inflow	-54.4518	-2.4916	turbid
T1	Tapajós	Tapajós upstream	-55.1013	-2.9714	clear
T2	Tapajós	Tapajós downstream	-54.9001	-2.4315	clear
T3	Tapajós	Tapajós downstream	-54.8332	-2.2742	clear
T	Tapajós	used for incubation	-54.7219	-2.4056	clear

Table S2. Description of samples in mixing and incubation experiments.

Sample	water mixture	incubation time
FSFN	50% Solimões + 50% Negro	0 minutes
S50N50_30min	50% Solimões + 50% Negro	30 minutes
S20N80_1d	20% Solimões + 80% Negro	one day
S40N60_1d	40% Solimões + 60% Negro	one day
S50N50_1d	50% Solimões + 50% Negro	one day
S60N40_1d	60% Solimões +40% Negro	one day
S80N20_1d	80% Solimões + 20% Negro	one day
FSSN_B-1d	Solimões filtered water + Negro filtered suspended solids	one day at in situ temperature (30 °C)
FSSN_S-1d	Solimões filtered water + Negro filtered suspended solids	one day on ice (0 °C)
FNSS_B-1d	Negro filtered water + Solimões filtered suspended solids	one day at in situ temperature (30 °C)
FNSS_S-1d	Negro filtered water + Solimões filtered suspended solids	one day on ice (0 °C)
S20N80_5d	20% Solimões + 80% Negro	five days
S40N60_5d	40% Solimões + 60% Negro	five days
S50N50_5d_1	50% Solimões + 50% Negro	five days
S50N50_5d_2	50% Solimões + 50% Negro	five days
S50N50_5d_3	50% Solimões + 50% Negro	five days
S60N40_5d	60% Solimões + 40% Negro	five days
S80N20_5d	80% Solimões + 20% Negro	five days
A20T80_1d	20% Amazon + 80% Tapajós	one day
A40T60_1d	40% Amazon + 60% Tapajós	one day
A50T50_1d	50% Amazon + 50% Tapajós	one day
A60T40_1d	60% Amazon + 40% Tapajós	one day
A80T20_1d	80% Amazon + 20% Tapajós	one day
A20T80_5d	20% Amazon + 80% Tapajós	five days
A40T60_5d	40% Amazon + 60% Tapajós	five days
A50T50_5d	50% Amazon + 50% Tapajós	five days
A60T40_5d	60% Amazon + 40% Tapajós	five days
A80T20_5d	80% Amazon + 20% Tapajós	five days

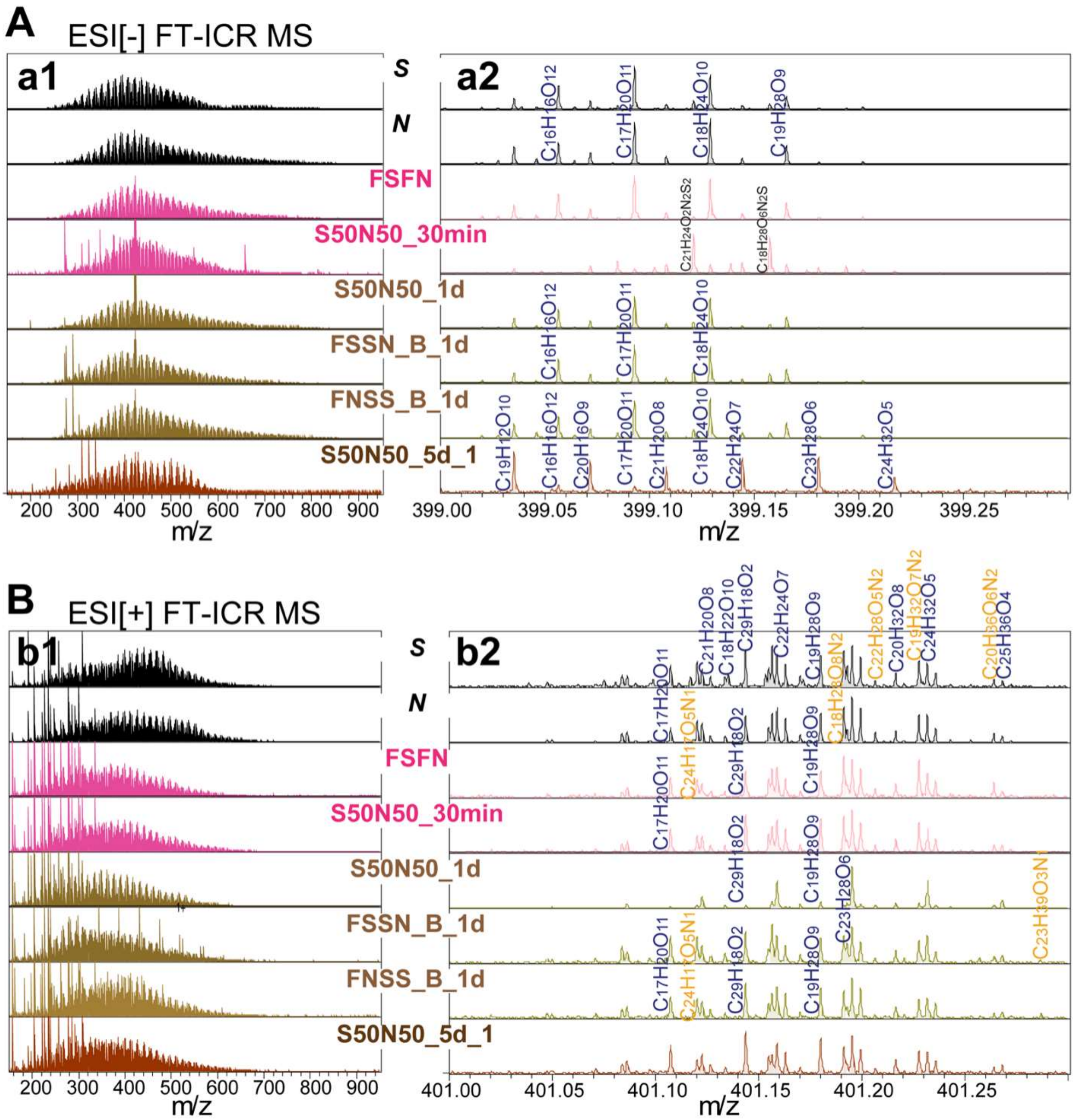

Figure S1. (A) ESI[-] and (B) ESI[+] FT-ICR mass spectra of SPE-DOM in unmixed and mixed S+N water pre- and post- incubation. The FT-ICR mass spectra are exemplified with samples *S*, *N*, FSFN, S50N50_30min, S50N50_1d, FSSN_B_1d, FNSS_B_1d, and S50N50_5d_1. The ESI[-] and ESI [+] FT-ICR mass spectra in (a1) and (b1) show the regular shaped signal distribution over a wide mass range (m/z 150-950), as well as distinct signatures at nominal neutral mass 400. Respective assignments of molecular compositions are provided for CHO (blue), CHNO (orange), and few CHNOS (black) molecules (a2).

Table S3. ESI[-] FT-ICR MS derived counts of mass peaks and intensity-weighted average bulk parameters for all assigned molecular compositions present in SPE-DOM in unmixed and mixed S+N water pre- and post- incubation. FT-ICR MS derived bulk parameters comprise percentages of counts of CHO, CHNO, CHOS, and CHNOS molecular classes, computed experimental *m/z*, DBE/C, H/C, N/C, and S/C ratios.

Sample	total counts of mass peaks	CHO%	CHNO%	CHOS%	CHNOS%	*m/z*	DBE/C	H/C	O/C	N/C×10^{-2}	S/C ×10^{-3}
S	5043	59.5	32.4	7.2	1.0	460.2	0.53	1.06	0.55	0.76	1.12
SM	4408	65.7	29.0	5.1	0.2	479.9	0.52	1.06	0.54	0.60	0.79
N	4233	74.4	23.9	1.7	0.0	496.5	0.53	1.04	0.54	0.31	0.23
NaM	3781	76.9	21.8	1.4	0.0	498.3	0.53	1.04	0.54	0.29	0.17
MZ	4424	73.4	24.8	1.8	0.0	492.3	0.52	1.05	0.53	0.33	0.23
A1	4675	68.7	27.7	3.6	0.0	488.9	0.52	1.05	0.54	0.45	0.43
A2	4468	69.0	27.3	3.7	0.0	485.1	0.52	1.07	0.54	0.47	0.64
AM1	4120	63.8	30.4	5.5	0.3	467.9	0.52	1.08	0.54	0.74	1.01
AM2	4522	67.2	28.6	4.1	0.1	482.9	0.51	1.07	0.54	0.55	0.59
FSFN	3719	74.3	23.7	2.0	0.0	488.5	0.53	1.04	0.55	0.40	0.43
S50N50_30min	2284	78.9	19.6	1.5	0.0	477.9	0.45	1.19	0.43	0.35	0.86
S20N80_1d	3777	75.8	23.0	1.2	0.0	492.7	0.53	1.04	0.54	0.33	0.33
S40N60_1d	3886	73.5	24.6	2.0	0.0	487.3	0.53	1.04	0.55	0.37	0.53
S50N50_1d	4188	70.9	26.8	2.3	0.0	485.3	0.53	1.04	0.55	0.43	0.35
S60N40_1d	4157	70.6	26.1	3.3	0.0	484.8	0.53	1.04	0.55	0.45	0.51
S80N20_1d	4205	69.1	27.4	3.5	0.0	479.1	0.52	1.05	0.54	0.48	0.53
FSSN_B_1d	4017	66.7	28.4	4.9	0.0	470.8	0.51	1.08	0.54	0.57	1.18
FSSN_S_1d	3707	67.4	28.6	3.9	0.1	469.5	0.52	1.07	0.55	0.58	0.75
FNSS_B_1d	3689	75.9	22.7	1.4	0.0	489.6	0.52	1.05	0.55	0.31	0.34
FNSS_S_1d	3699	76.2	22.1	1.8	0.0	492.4	0.52	1.05	0.54	0.30	0.64
S20N80_5d	4251	71.1	26.4	2.5	0.1	483.8	0.52	1.05	0.53	0.39	0.35
S40N60_5d	4260	69.6	27.1	3.3	0.0	477.5	0.52	1.05	0.53	0.44	0.42
S50N50_5d_1	1719	93.6	5.4	1.1	0.0	439.6	0.51	1.08	0.41	0.90	0.93
S50N50_5d_2	4229	66.4	27.4	6.2	0.1	455.7	0.54	1.03	0.56	0.51	0.87
S50N50_5d_3	1555	94.0	4.4	1.7	0.0	431.8	0.51	1.07	0.42	0.87	2.17
S60N40_5d	2599	77.0	20.6	2.4	0.1	477.1	0.43	1.23	0.41	0.43	0.70
S80N20_5d	4301	67.5	28.1	4.4	0.0	482.6	0.53	1.05	0.54	0.51	0.61

Footnote: 1) FSFN and S50N50_30min show much less numerous assigned mass peaks, higher %CHO and lower %CHNO molecules; 2) Small decrease of average m/z at 5d; 3) Some decline of O/C ratio at 5d; 4) The average S/C ratio was higher in FSSN_B_1d, showing that more S-containing molecules in the mixture of filtered Solimões River water and suspended solids from the Negro River after incubation at in situ temperature; 5) The lower S/C ratio in FSSN_S_1d than in FSSN_B_1d was likely because the production of S-containing molecules was slowed down under lower processing.

Table S4. ESI[+] FT-ICR MS derived counts of mass peaks and intensity-weighted average bulk parameters for all assigned molecular compositions present in SPE-DOM in unmixed and mixed S+N water pre- and post- incubation. FT-ICR MS derived bulk parameters comprise percentages of counts of CHO, CHNO, CHOS, and CHNOS molecular classes, computed experimental *m/z*, DBE/C, H/C, N/C, and S/C ratios.

Sample	total counts of mass peaks	CHO%	CHNO%	CHOS%	CHNOS%	*m/z*	DBE/C	H/C	O/C	$N/C\times10^{-2}$	$S/C\times10^{-3}$
S	4967	29.5	70.2	0.1	0.2	439.2	0.40	1.34	0.41	3.89	0.02
SM	5172	28.5	71.0	0.2	0.3	413.2	0.38	1.38	0.37	4.05	0.06
N	5727	30.0	69.9	0.1	0.0	442.5	0.39	1.35	0.40	3.63	0.03
NaM	5428	28.9	70.9	0.2	0.0	439.2	0.39	1.36	0.39	3.66	0.03
MZ	5808	28.6	71.2	0.2	0.0	437.7	0.38	1.38	0.39	3.76	0.05
A1	5159	30.0	69.7	0.1	0.2	422.3	0.39	1.37	0.39	3.95	0.03
A2	4949	29.6	70.1	0.2	0.0	417.1	0.39	1.37	0.38	4.11	0.04
AM1	5081	27.7	71.6	0.2	0.5	407.2	0.39	1.38	0.38	4.25	0.06
AM2	5377	29.2	70.1	0.2	0.5	419.1	0.39	1.37	0.39	4.03	0.06
FSFN	3272	35.2	64.7	0.1	0.0	392.9	0.39	1.36	0.38	3.78	0.06
S50N50_30min	3759	35.1	64.8	0.1	0.1	400.0	0.40	1.35	0.40	3.51	0.11
S20N80_1d	2385	39.5	60.3	0.2	0.0	382.6	0.38	1.39	0.36	3.44	0.21
S40N60_1d	2876	39.3	60.3	0.4	0.0	384.9	0.38	1.38	0.38	3.45	0.34
S50N50_1d	3378	46.1	53.6	0.3	0.0	385.0	0.40	1.32	0.31	1.85	0.10
S60N40_1d	2732	37.5	62.4	0.2	0.0	378.8	0.38	1.38	0.37	3.57	0.18
S80N20_1d	3084	39.4	60.4	0.2	0.0	386.7	0.39	1.37	0.38	3.49	0.13
FSSN_B_1d	3837	34.7	64.9	0.2	0.2	396.5	0.41	1.33	0.41	3.62	0.13
FSSN_S_1d	3827	40.4	59.3	0.2	0.1	391.0	0.43	1.29	0.41	2.98	0.12
FNSS_B-_1d	3343	39.3	60.6	0.1	0.0	405.9	0.41	1.32	0.42	3.23	0.16
FNSS_S_1d	3355	38.4	61.4	0.2	0.0	400.8	0.40	1.34	0.4	3.25	0.13
S20N80_5d	3424	37.9	61.9	0.2	0.1	401.2	0.38	1.38	0.38	3.42	0.11
S40N60_5d	3058	37.7	62.1	0.2	0.0	388.2	0.37	1.4	0.37	3.43	0.12
S50N50_5d_1	3692	36.6	63.3	0.1	0.0	403.1	0.40	1.34	0.41	3.39	0.08
S50N50_5d_2	3831	43.4	56.4	0.2	0.1	401.0	0.43	1.28	0.42	2.66	0.08
S50N50_5d_3	2771	42.7	57.2	0.0	0.0	388.5	0.38	1.37	0.27	2.90	0.03
S60N40_5d	3238	34.8	63.6	1.5	0.1	393.0	0.35	1.43	0.29	3.11	1.40
S80N20_5d	2820	35.6	61.9	2.3	0.1	378.3	0.39	1.37	0.37	3.70	2.41

Footnote: 1) Strong decrease of counts: original > 1d, 5d; 2) Strong increase of %CHO, decrease of %CHNO and some decrease of N/C ratio from original to 1d, 5d; 3) Decrease of m/z from original to 1d, 5d; 4) Increase of S/C ratio and %CHOS.

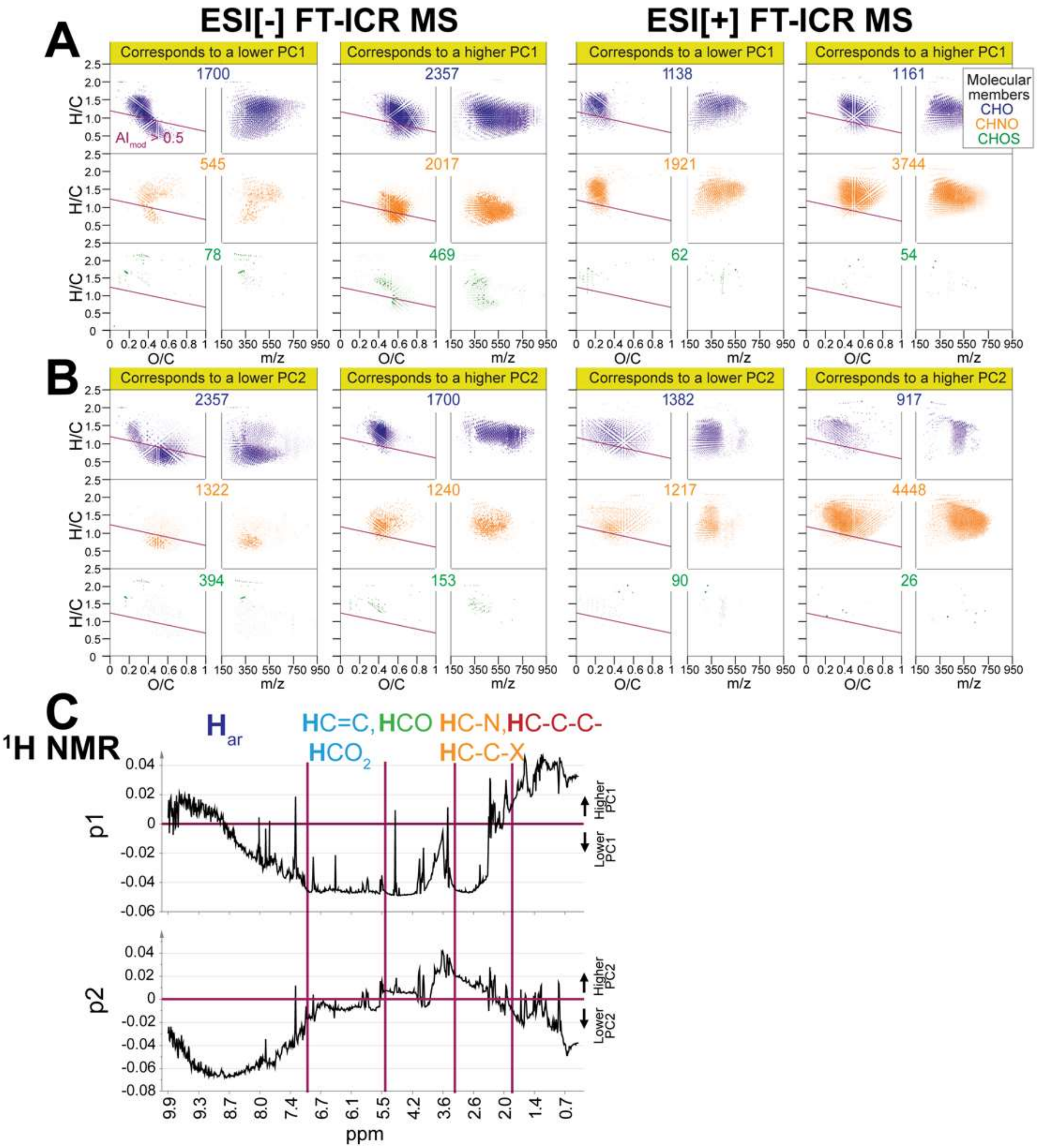

Figure S2. Loading vectors in PCA of SPE-DOM in unmixed and mixed S+N water pre- and post-incubation based on all assigned molecular formulae in (A, B) ESI[±] FT-ICR mass spectra and (C) ^{1}H NMR spectra . The scatter plots of the very PCA see Figure 2. Panels A and B show correlation patterns of PC1 and PC2 loading vectors (p1 and p2) in van Krevelen and mass-edited H/C diagrams, with the color code representing CHO (blue), CHNO (orange), and CHOS (green) molecular classes. The numbers show counts of compounds. The molecular formulae positioned below the purple line in the van Krevelen diagrams have modified aromaticity index (AI_{mod})[15] higher than 0.5. Panel C show correlation patterns of p1 and p2 in ^{1}H NMR space, with fundamental molecular structures indicated.

Table S5. Water parameters in unmixed and mixed S+N and A+T waters pre- and post- incubation.

Sample	Temp. (ºC)	pH	DOC (mg/L)	POC (mg/L)	TOC (mg/L)	Cond. (mg/L)	DO (mg/L)	NH_4^+ (µM)	NO_3^- (µM)	NO_2^- (µM)	PO_4^{3-} (µM)
S	28.0	7.0	4.56	13.94	18.50	84	6.4	0.42	0.26	0.10	0.10
N	29.0	4.7	9.96	12.54	22.50	17	6.2	0.68	0.41	0.09	0.10
A1	28.8	6.4	5.36	1.44	6.80	85	4.5	0.53	0.94	0.08	0.24
A2	28.5	6.4	4.85	0.90	5.82	79	3.9	0.63	0.69	0.05	0.69
Ad1	27.5	6.5	4.16	0.23	4.39	56	5.0	0.88	0.59	0.15	0.22
Ad2	29.1	6.5	4.68	0.19	4.87	51	4.9	0.73	0.71	0.12	0.11
A	29.9	6.8	5.51	0.90	6.41	69	6.0	2.32	0.33	0.13	0.10
At	27.8	6.8	3.63	0.39	4.02	35	7.2	0.47	1.55	0.04	0.15
T1	26.9	5.7	3.11	0.23	3.34	37	5.8	0.42	0.57	0.10	0.12
T2	29.5	6.6	3.48	0.17	3.52	34	7.8	0.41	0.47	0.02	0.11
T3	29.9	6.8	5.51	0.85	6.41	61	6.0	2.32	0.33	0.13	0.10
T	29.2	6.6	7.03	0.17	7.20	55	4.7	2.36	0.71	0.12	0.14
S20N80_1d	27.2	6.79	N.A.	N.A.	N.A.	25.6	N.A.	0.59	0.52	0.08	0.11
S40N60_1d	28.0	7.04	N.A.	N.A.	N.A.	34.8	N.A.	0.62	0.49	0.10	0.13
S60N40_1d	28.1	7.25	N.A.	N.A.	N.A.	49.4	N.A.	0.56	0.44	0.10	0.11
S80N20_1d	N.A.	7.43	N.A.	N.A.	N.A.	60.6	N.A.	0.62	0.49	0.07	0.12
S20N80_5d	29.1	6.48	N.A.	N.A.	N.A.	25.2	N.A.	1.35	0.75	0.13	0.11
S40N60_5d	29.1	6.74	N.A.	N.A.	N.A.	40.9	N.A.	1.23	0.69	0.13	0.11
S60N40_5d	28.7	7.8	N.A.	N.A.	N.A.	57.6	N.A.	1.22	0.71	0.12	0.13
S80N20_5d	29.1	7.18	N.A.	N.A.	N.A.	68.9	N.A.	0.94	0.69	0.12	0.12

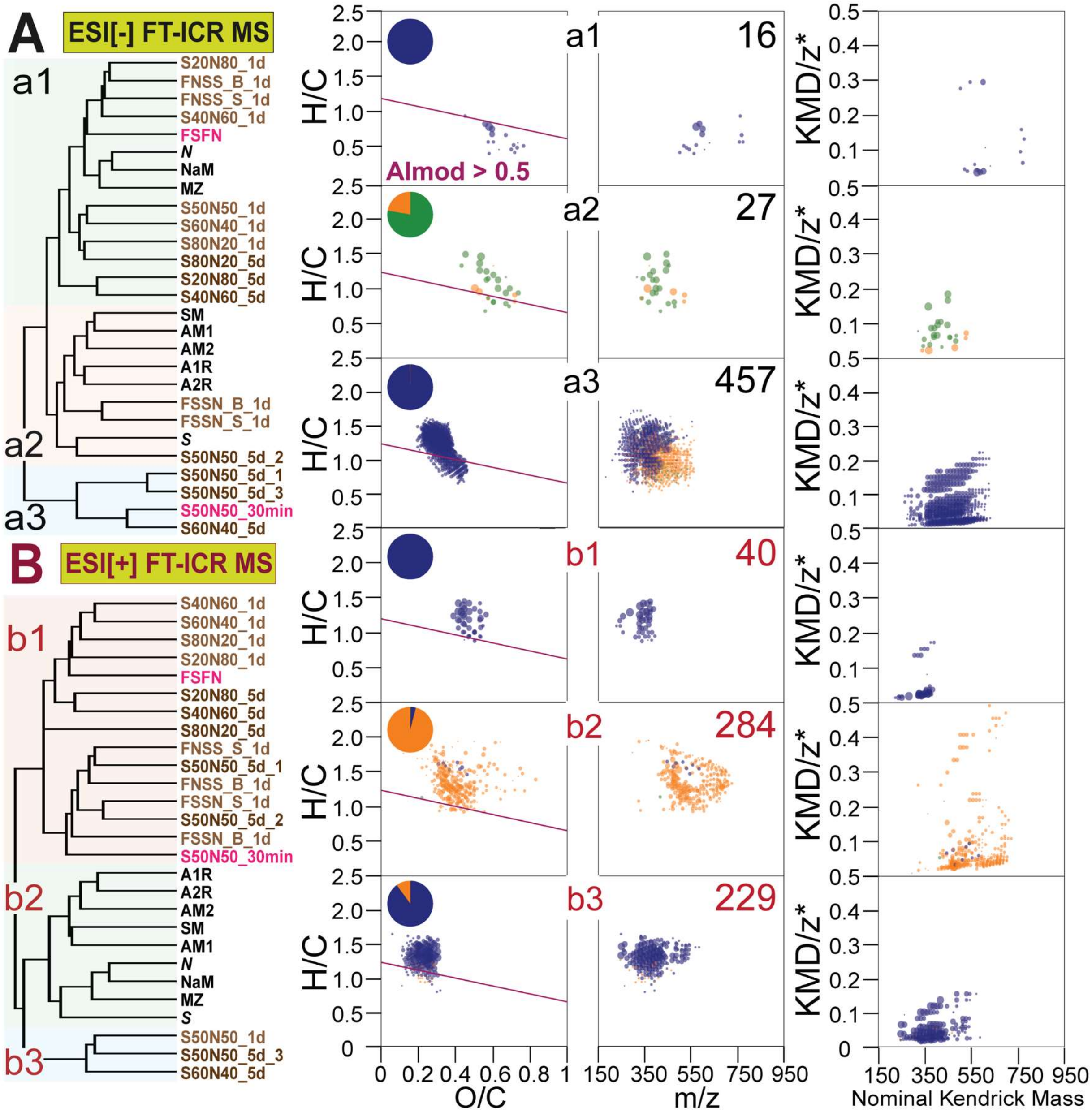

Figure S3. HCA of assigned SPE-DOM molecular formulae in part of the unmixed and mixed S+N water pre- and post- incubation. Van Krevelen, mass-edited H/C, and KMD/z* diagrams[16] show CHO (blue), CHNO (orange), and CHOS (green) molecular classes that were relatively more abundant in clusters a1/a2/a3, b1/b2/b3, respectively. Bubble areas represent the normalized intensities of SPE-DOM in each cluster. The pie plots depict percentages of the counts of different molecular classes. The numbers show counts of compounds. The molecular formulae positioned below the purple line in the van Krevelen diagrams have modified aromaticity index (AI_{mod})[15] higher than 0.5.

Footnote: 1) ESI[±] MS-derived HCA separated samples in clusters a3 and b3 owing to their higher abundance in less oxygenated CHO compounds, in line with the PCA results (Figure 2AB); 2) ESI[+] MS-derived HCA separated river water samples (b2) and experimentally mixed water samples (b1,b3). SPE-DOM in river waters were more abundant in some CHNO ions (*m/z* 450-700) than SPE-DOM in mixed waters.

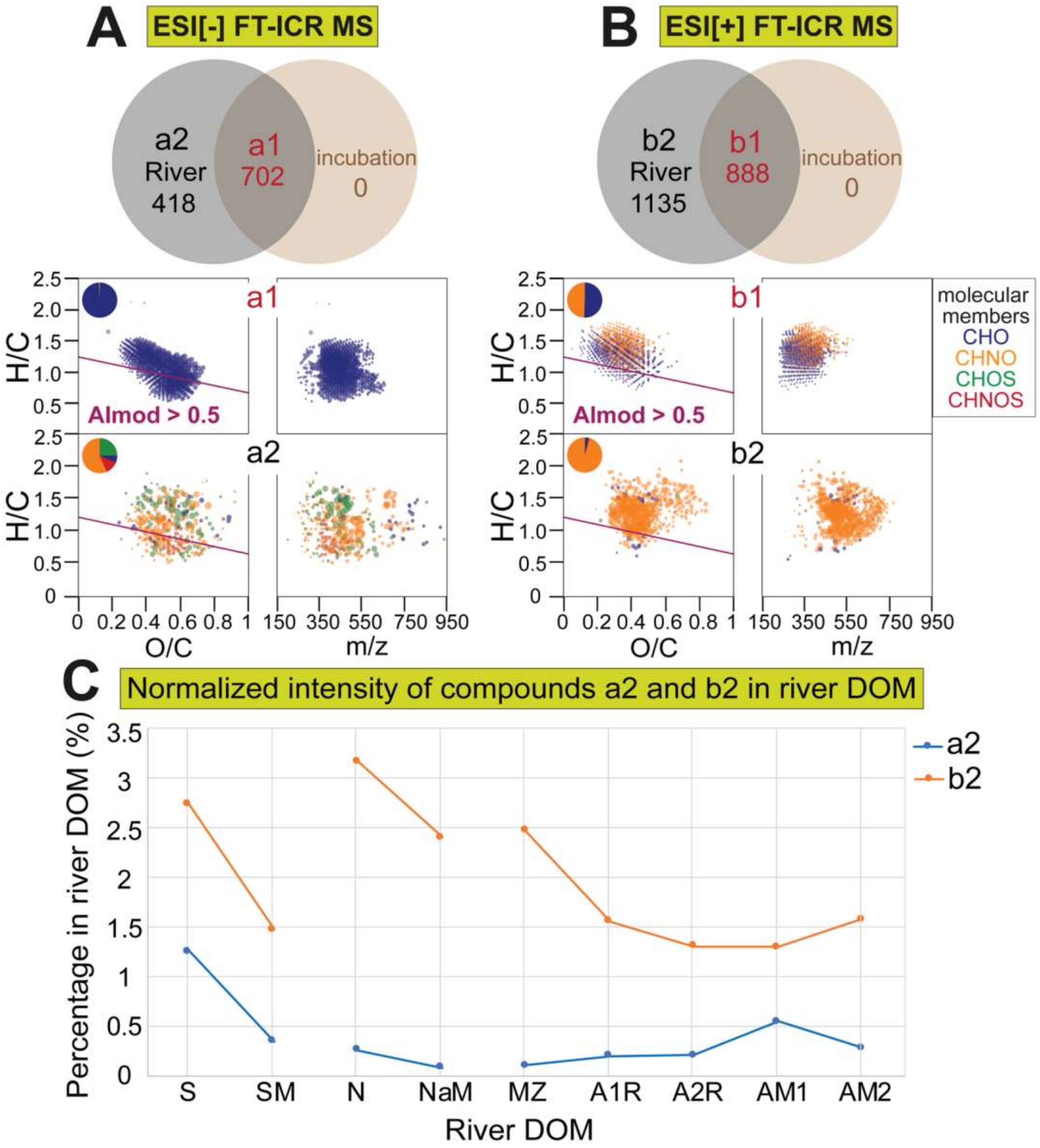

Figure S4. Common and unique molecular signatures in SPE-DOM in the unmixed and mixed S+N water pre- and post- incubation. The numbers show counts of compounds. Van Krevelen and mass-edited H/C diagrams show CHO (blue), CHNO (orange), CHOS (green), and CHNOS (red) m/z ions that were shared before and after incubation (a1 and b1); unique in original water but were not in incubated water (a2 and b2). Not any single compound unique to incubated water did not present in original water. Bubble areas represent the normalized intensity of DOM. The pie plots depict percentages of the counts of different molecular classes. Molecular composition positioned below the purple line in the van Krevelen diagrams shows the compounds with a modified aromaticity index (AI_{mod}) higher than 0.5[15]. Panel C shows the normalized intensity of molecular signatures that were degraded during the incubation process (a2 and b2) in DOM of the Amazon mainstem.

Figure S4. Footnote: 1) Two sets of overlapping CHO molecules with good coverage of the compositional space with (A) average H/C rand O/C ratios (*m/z* ~350~570) and (B) more unsaturated and highly oxygenated lignin-like molecules (*m/z* ~350~620); 2) A set of more unsaturated, low mass (*m/z* < 380) CHO molecules and another set of more saturated CHNO molecules with limited range of oxygenation and mass; 3) Interspersed set of CHNO, CHOS, CHNOS molecules with high chemodiversity covering large areas in van Krevelen diagrams; 4) Two sets of considerably unsaturated CHO molecules with distinct oxygenation.

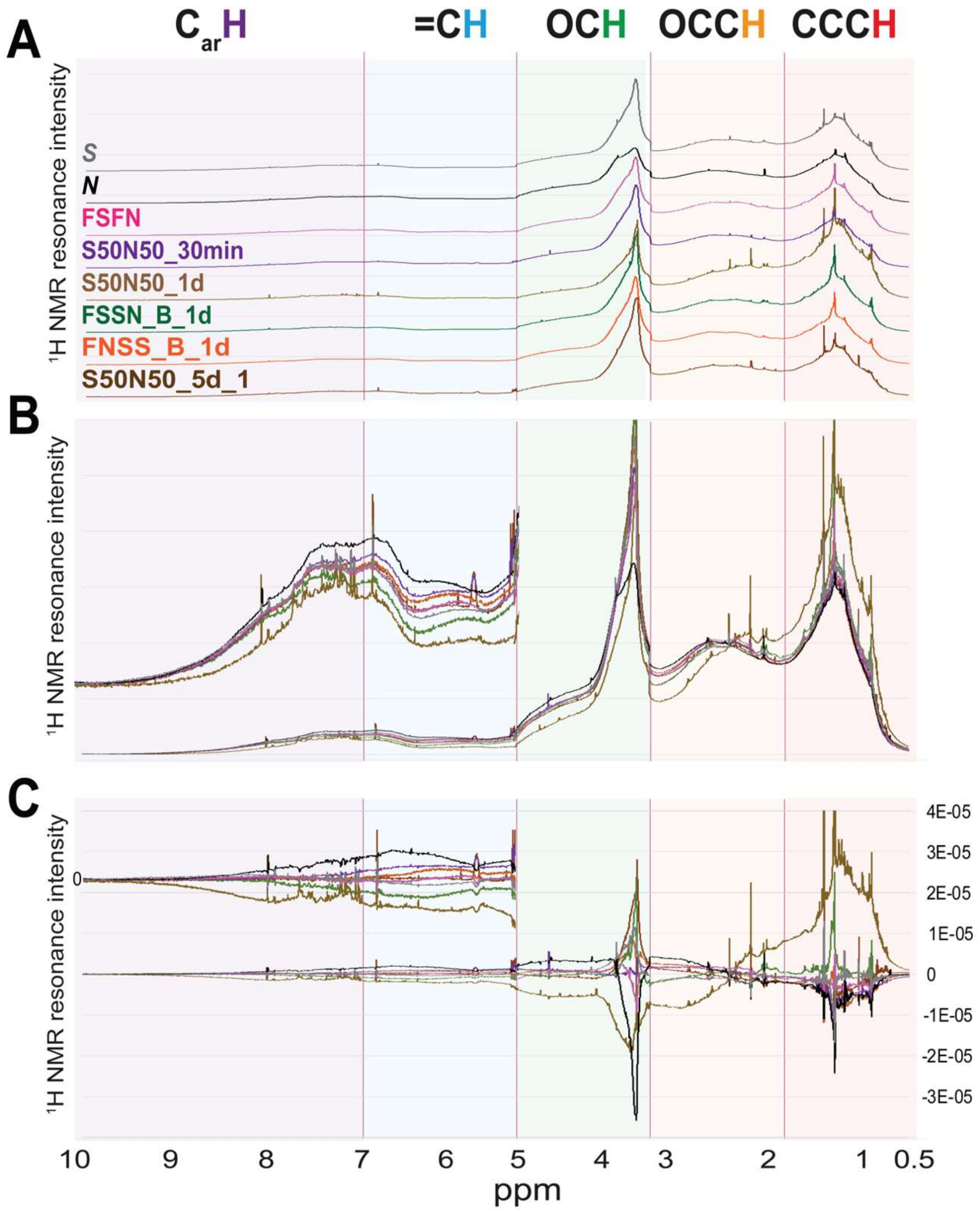

Figure S5. ^{1}H NMR spectra of SPE-DOM in the unmixed and mixed S+N water pre- and post-incubation. The ^{1}H NMR spectra are exemplified with samples *S*, *N*, FSFN, S50N50_30min, S50N50_1d, FSSN_B_1d, FNSS_B_1d, and S50N50_5d_1. ^{1}H NMR spectra are area normalized (800 MHz, CD_3OD; δH = 0.5-10.0 ppm with the exclusion of residual water and methanol NMR resonances). The ^{1}H NMR spectra referred to average abundance are edited by each normalized spectra subtract the average spectra (C).

Table S6. ^{1}H NMR section integrals (percent of non-exchangeable protons, 800 MHz; CD_3OD, exclusion of residual water, and methanol)) and key substructures of SPE-DOM in the unmixed and mixed S+N water pre- and post- incubation. The fundamental substructures include aromatics C_{ar}**H**, δ_H ~ 7.0-10.0 ppm; olefins δ_H ~ 5.3-7.0 ppm; oxygenated aliphatic units (OC**H**) and "carbohydrate-like" and methoxy OC**H**$_3$ units δ_H ~ 3.2-4.9 ppm; branched aliphatic units (C**H**$_2$)$_n$, "acetate-analogue" and CRAM δ_H ~ 1.9-3.2 ppm; functionalized aliphatics δ_H ~ 1.35-1.9 ppm; polyethylene group δ_H ~ 1.25-1.35 ppm, OCC**H** units; pure aliphatics δ_H ~ 0.5-1.25 ppm, CCC**H** units.

δ (^{1}H) [ppm]	10.0-7.0	7.0_5.3	4.9-3.2	3.2-1.9	1.9-1.35	1.35-1.25	1.25-0.5	1.9-0.5
key substructures	C_{ar}**H** (%)	C=C**H**, O_2C**H** (%) / O_2C**H** (%)	OC**H** (%)	OCCH (%)	OCCC**H** (%)	(C**H**$_2$)$_n$ (%)	CCCH$_3$ (%)	total aliphatic section (%)
S	*5.8*	*4.6*	*30.3*	*28.0*	*14.5*	*4.0*	*12.8*	*31.3*
SM	5.1	3.5	26.6	29.6	15.5	5.1	14.6	35.3
N	*5.9*	*5.8*	*26.8*	*30.7*	*14.8*	*4.2*	*11.8*	*30.8*
NaM	5.5	5.2	26.5	30.9	15.0	4.5	12.4	31.9
MZ	5.9	4.3	24.6	29.0	16.8	5.5	13.8	36.2
A1	4.6	4.1	25.1	31.7	16.3	4.9	13.3	34.5
A2	5.4	4.8	27.4	30.4	15.1	4.4	12.4	31.9
AM1	4.8	4.5	27.7	30.9	15.3	4.5	12.3	32.1
AM2	5.0	4.8	28.6	30.4	15.0	4.3	11.8	31.2
FSFN	5.6	4.8	28.6	30.0	14.4	4.2	12.3	31.0
S50N50_30min	5.7	5.7	29.2	29.7	14.4	3.9	11.5	29.8
S20N80_1d	4.8	2.7	24.8	28.1	14.3	9.0	16.2	39.6
S40N60_1d	4.7	2.9	28.9	29.5	14.4	6.3	13.2	34.0
S50N50_1d	4.0	2.9	21.7	26.9	18.7	6.0	19.7	44.5
S60N40_1d	4.2	2.3	27.0	28.0	14.3	9.3	14.9	38.5
S80N20_1d	4.7	1.1	27.4	26.1	14.3	9.9	16.5	40.7
FSSN_B_1d	5.1	3.9	29.1	28.8	15.1	4.9	13.1	33.1
FSSN_S_1d	5.0	3.6	30.2	28.4	14.7	4.6	13.5	32.8
FNSS_B_1d	5.6	5.3	29.9	29.6	14.1	4.2	11.3	29.6
FNSS_S_1d	5.7	5.4	30.9	29.5	13.7	4.0	10.9	28.6
S20N80_5d	4.5	3.7	26.7	27.9	15.0	6.6	15.6	37.2
S40N60_5d	4.3	3.6	26.9	29.8	15.3	5.7	14.5	35.4
S50N50_5d_1	5.5	5.0	30.3	28.8	14.0	4.0	12.5	30.4
S50N50_5d_2	6.4	5.9	29.0	29.1	14.1	3.8	11.7	29.6
S50N50_5d_3	6.9	2.2	17.2	25.9	17.8	6.9	23.1	47.8
S60N40_5d	3.9	1.9	15.0	25.5	19.0	12.7	22.0	53.7
S80N20_5d	3.9	2.2	28.8	28.0	14.7	7.0	15.5	37.1

Footnote: 1) Nearly unchanged C_{ar}**H**; 2) Considerable decrease of olefins =C**H**; 3) uneven evolution in general.

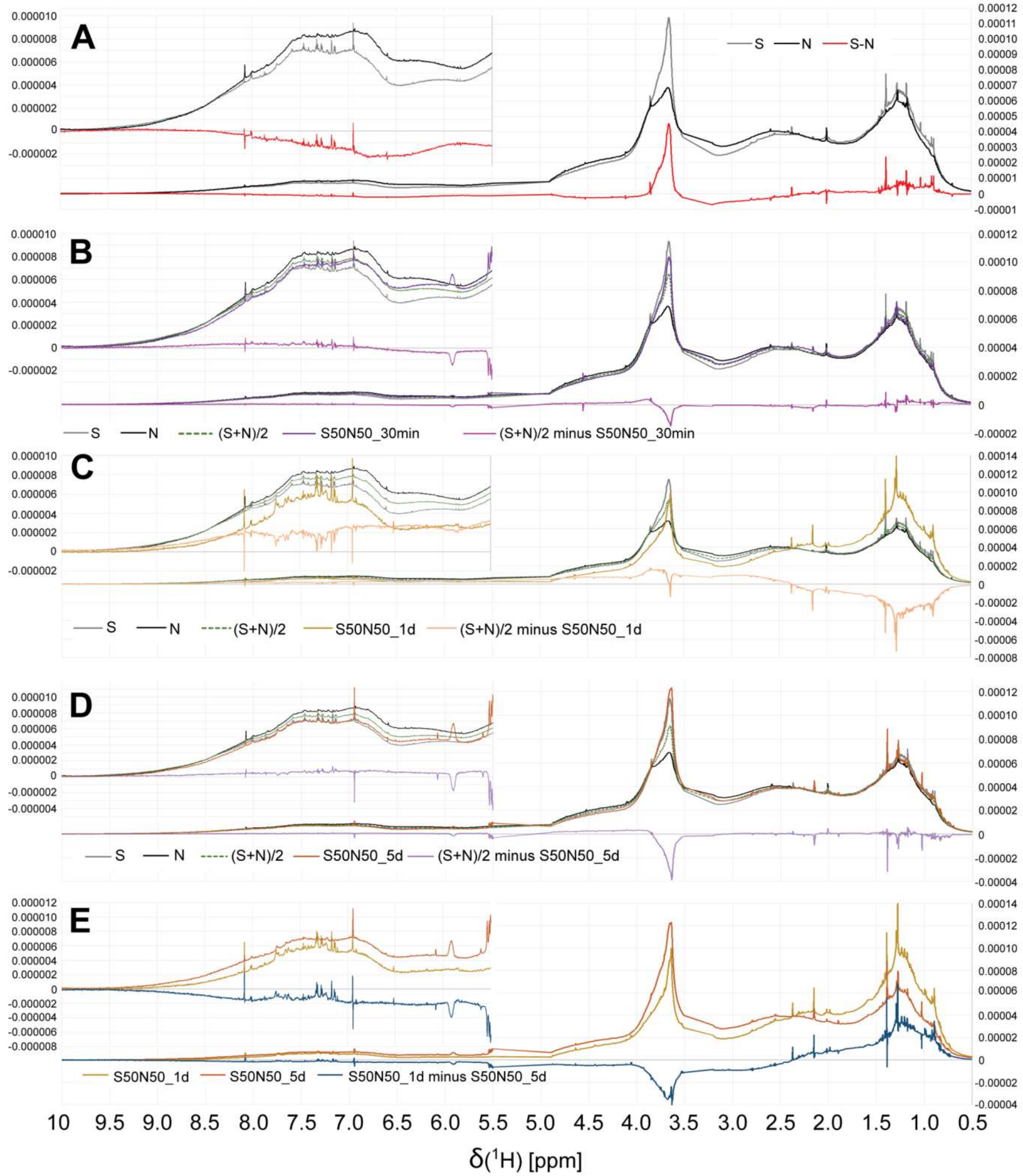

Figure S6. Difference ^{1}H NMR spectra (800 MHz, CD_3OD) of Solimões and Negro SPE-DOM according to status of incubation. (A) Solimões minus Negro, with Solimões (S), Negro (N), and Solimões minus Negro (S-N). (B) difference ^{1}H NMR spectrum after 30 minutes of incubation, with S, N, averaged Solimões and Negro ((S+N)/2), S50N50_30min, and (S+N)/2 minus S50N50_30min. (C) difference ^{1}H NMR spectrum after one day of incubation, with S, N, (S+N)/2, S50N50_1d, and (S+N)/2 minus S50N50_1d. (D) difference ^{1}H NMR spectrum after five days of incubation, with S, N, (S+N)/2, S50N50_5d, and (S+N)/2 minus S50N50_5d. (E) difference ^{1}H NMR spectrum of one day minus five days of incubation, with S50N50_1d, S50N50_5d, and (S50N50_1d minus S50N50_5d).

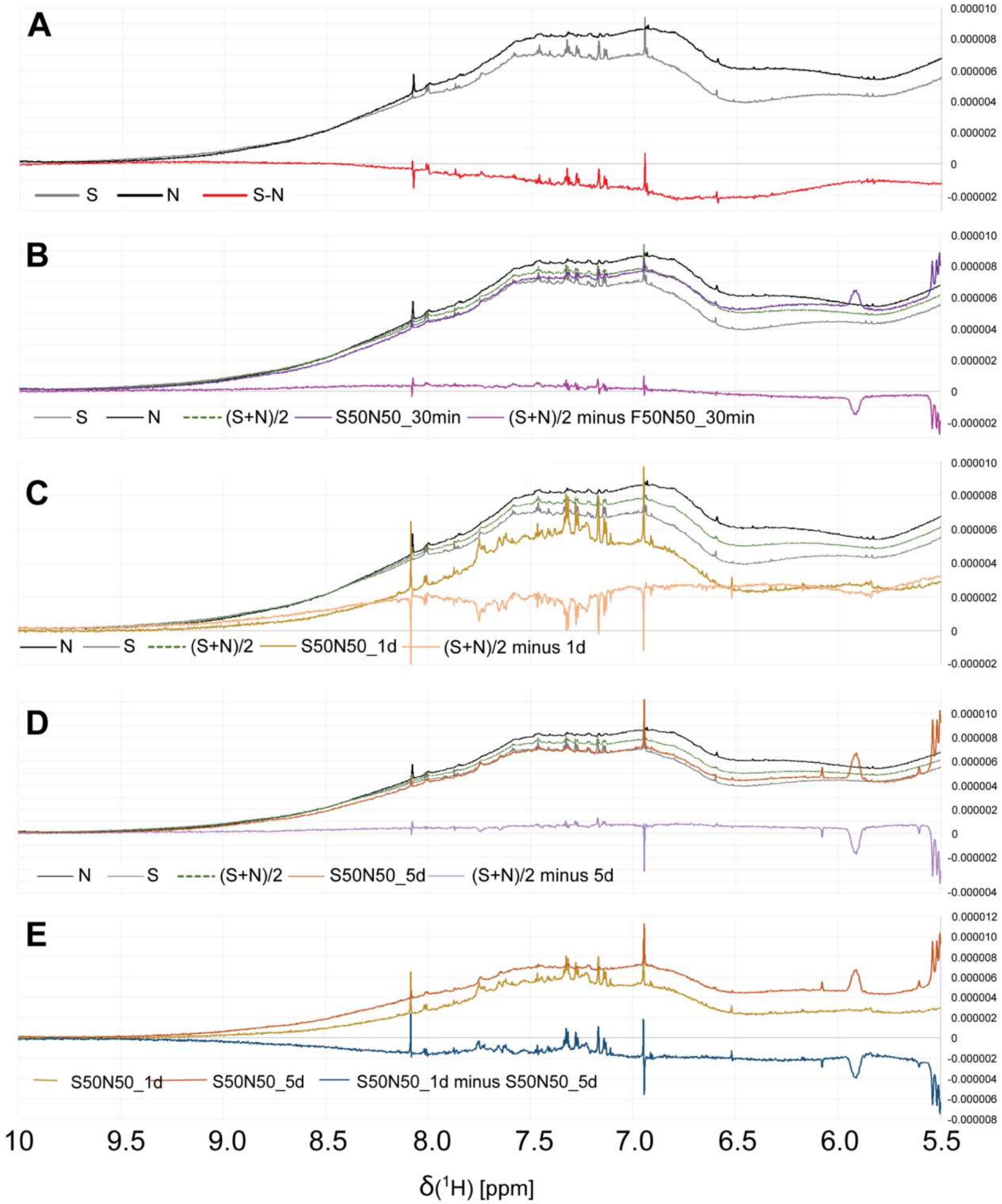

Figure S7. Difference ^{1}H NMR spectra (800 MHz, CD_3OD) of Solimões and Negro SPE-DOM according to status of incubation, section of unsaturated $C_{sp2}H$ protons δ_H ~ 5.5-10 ppm in Figure S6. (A) Solimões minus Negro, with Solimões (S), Negro (N), and Solimões minus Negro (S-N). (B) difference after 30 minutes of incubation, with S, N, averaged Solimões and Negro ((S+N)/2), S50N50_30min, and (S+N)/2 minus S50N50_30min. (C) difference after one day of incubation, with S, N, (S+N)/2, S50N50_1d, and (S+N)/2 minus S50N50_1d. (D) difference ^{1}H NMR spectrum after five days of incubation, with S, N, (S+N)/2, S50N50_5d, and (S+N)/2 minus S50N50_5d. (E) difference ^{1}H NMR spectrum one day minus five days of incubation, with S50N50_1d, S50N50_5d, and (S50N50_1d minus S50N50_5d).

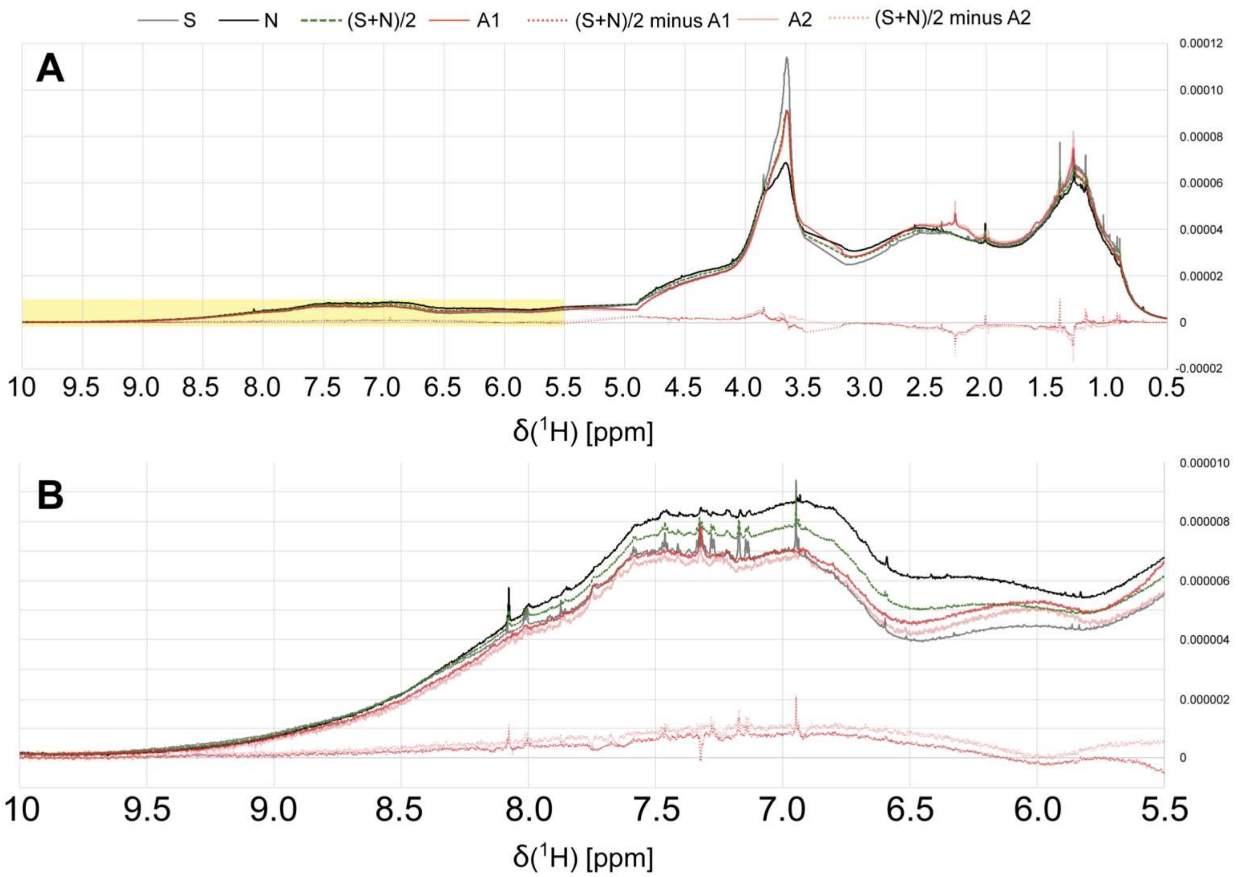

Figure S8. Difference ^{1}H NMR spectra (800 MHz, CD_3OD) of Solimões and Negro river waters, entire section δ_H ~ 0.5-10 ppm), and proximate Amazon SPE-DOM samples A1 and A2 (sampling locations see Figure 1 and Table S1). (A) (averaged Solimões and Negro) river minus proximate downstream Amazon River sampling points A1 (orange, dotted line) and A2 (pink, dotted line), with Solimões (S, grey), Negro (N, black), and averaged Solimões and Negro ((S+N)/2, dotted green line). (B) same spectra, section of unsaturated $C_{sp2}H$ protons (δ_H ~ 5.5-10 ppm).

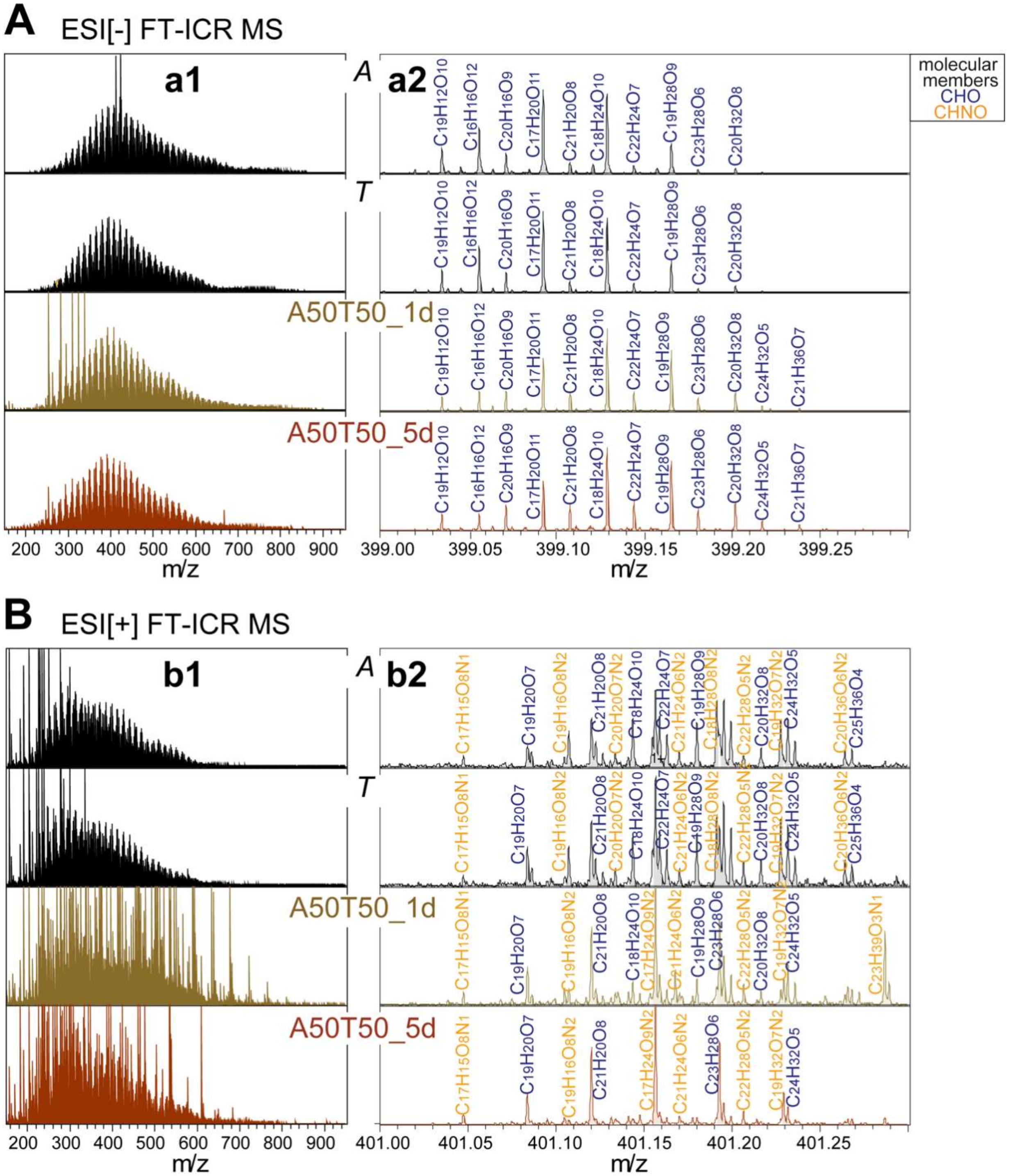

Figure S9. (A) ESI[-] and (B) ESI[+] FT-ICR mass spectra of SPE-DOM in unmixed and mixed A+T water pre- and post- incubation. The FT-ICR mass spectra are exemplified with samples *A*, *T*, A50T50_1d, and A50T50_5d. The ESI[-] and ESI [+] FT-ICR mass spectra in (a1) and (b1) show the regular shaped signal distribution over a wide mass range (m/z 150-950), as well as distinct signatures at nominal neutral mass 400. Respective assignments of molecular compositions are provided for CHO (blue) and CHNO (orange) molecules.

Table S7. ESI[-] FT-ICR MS derived counts of mass peaks and intensity-weighted average bulk parameters for all assigned molecular compositions present in unmixed and mixed A+T water pre- and post- incubation. FT-ICR MS derived bulk parameters comprise percentages of counts of CHO, CHNO, CHOS, and CHNOS molecular classes, computed experimental *m/z*, DBE/C, H/C, N/C, and S/C ratios.

Sample	Total counts of mass peaks	CHO%	CHNO%	CHOS%	CHNOS%	*m/z*	DBE/C	H/C	O/C	N/C $\times10^{-2}$	S/C $\times10^{-3}$
Ad1	4473	64.1	30.2	5.6	0.2	478.5	0.52	1.07	0.55	0.53	0.91
Ad2	4515	64.2	30.7	4.9	0.2	476.1	0.52	1.07	0.54	0.54	0.81
A	4787	65.0	30.2	4.7	0.0	480.2	0.52	1.06	0.54	0.55	0.54
At	3870	68.2	29.5	2.3	0.0	479.8	0.52	1.06	0.55	0.58	0.39
T1	5276	62.4	33.8	3.8	0.0	472.1	0.52	1.07	0.55	0.68	0.83
T2	5858	60.9	35.3	3.8	0.1	466.3	0.53	1.05	0.54	0.79	0.99
T3	5813	58.7	32.1	6.9	2.2	446.6	0.52	1.06	0.55	0.71	1.21
T	5898	57.9	34.4	6.5	1.2	462.4	0.50	1.11	0.51	0.76	0.94
A20T80_1d	5713	66.2	30.8	3.1	0.0	478.2	0.45	1.20	0.46	0.61	0.78
A40T60_1d	5186	67.6	29.9	2.5	0.0	469.6	0.46	1.19	0.46	0.58	0.52
A50T50_1d	6210	63.7	32.4	3.9	0.0	475.7	0.44	1.22	0.45	0.63	1.77
A60T40_1d	5072	68.3	28.9	2.7	0.0	463.3	0.46	1.19	0.46	0.56	0.58
A80T20_1d	5837	65.2	31.5	3.3	0.0	480.0	0.45	1.19	0.46	0.61	0.71
A20T80_5d	4730	68.6	28.5	2.9	0.0	455.0	0.45	1.20	0.45	0.58	0.65
A40T60_5d	3694	72.4	24.6	3.0	0.0	427.6	0.43	1.24	0.44	0.67	3.01
A50T50_5d	4773	68.8	28.1	3.2	0.0	453.7	0.44	1.22	0.44	0.56	0.69
A60T40_5d	4690	68.3	28.3	3.4	0.0	451.7	0.45	1.21	0.45	0.58	0.85
A80T20_5d	4699	69.5	27.2	3.3	0.0	450.1	0.44	1.22	0.44	0.55	1.01

Footnote: 1) No clear trend in count of mass peaks and %element ratios when changing mixing ratio; 2) *m/z* overall decreases from 1d to 5d incubation; 3) Overall small gain in saturation as expressed by decrease in DBE/C and increase in H/C ratios during 5d incubation; 4) Considerable change from original to 1d and very minor loss of O/C ratio, most probably because of loss of oxygen-rich thermodynamic endmember molecules.

Table S8. ESI[+] FT-ICR MS derived counts of mass peaks and intensity-weighted average bulk parameters for all assigned molecular compositions present in unmixed and mixed A+T water pre- and post- incubation. FT-ICR MS derived bulk parameters comprise percentages of counts of CHO, CHNO, CHOS, and CHNOS molecular classes, computed experimental *m/z*, DBE/C, H/C, N/C, and S/C ratios.

Sample	Total counts of mass peaks	CHO%	CHNO%	CHOS%	CHNOS%	*m/z*	DBE/C	H/C	O/C	N/C $\times 10^{-2}$	S/C $\times 10^{-3}$
Ad1	6060	30.4	67.6	0.5	1.5	404.8	0.39	1.36	0.40	3.71	0.17
Ad2	5845	30.1	68.3	0.5	1.1	415.4	0.39	1.38	0.36	3.72	0.14
A	3943	32.0	68.0	0.0	0.1	395.0	0.39	1.37	0.38	3.95	0.02
At	5809	30.2	69.4	0.1	0.3	410.7	0.40	1.34	0.40	3.87	0.07
T1	4709	30.5	69.3	0.1	0.1	397.6	0.41	1.33	0.41	4.05	0.04
T2	4365	29.6	70.3	0.1	0.0	390.0	0.40	1.35	0.39	4.07	0.03
T3	4189	29.8	69.8	0.2	0.2	389.4	0.41	1.33	0.40	4.08	0.06
T	3564	32.3	67.5	0.1	0.1	386.7	0.39	1.36	0.39	3.91	0.04
A20T80_1d	3382	32.3	67.1	0.5	0.1	380.9	0.37	1.40	0.37	3.69	0.00
A40T60_1d	4070	34.0	65.5	0.4	0.1	387.6	0.39	1.37	0.37	3.66	0.00
A50T50_1d	3580	28.2	71.3	0.5	0.1	422.0	0.32	1.50	0.33	3.63	0.00
A60T40_1d	3131	35.6	63.9	0.4	0.1	376.5	0.38	1.39	0.37	3.60	0.01
A80T20_1d	2698	35.2	63.6	0.9	0.2	375.1	0.38	1.39	0.38	3.87	0.02
A20T80_5d	2574	33.0	65.8	1.0	0.2	362.6	0.38	1.39	0.35	4.24	0.02
A40T60_5d	2319	27.3	71.6	1.0	0.2	376.3	0.40	1.36	0.36	5.12	0.03
A50T50_5d	2578	31.3	67.7	0.9	0.2	364.0	0.39	1.38	0.34	4.12	0.01
A60T40_5d	2927	31.4	67.9	0.7	0.1	362.7	0.39	1.38	0.34	3.95	0.01
A80T20_5d	2015	31.5	67.1	1.4	0.1	357.6	0.38	1.40	0.35	4.55	0.03

Footnote: 1) Counts of assigned mass peaks and average *m/z* decrease during incubation (original > 1d > 5d); 2) %CHO increases from original water to 1d, and somewhat decreases from 1d to 5d; 3) While %CHNO remains near constant, %CHOS goes up (but S/C ratio goes down); 4) Average *m/z* decline from original to 1d to 5d; 5) Small initial decrease of DBE/C followed by "tiny rebound" from 1d to 5d; Similarly, H/C ratio increases "a little bit" from original to 1d; 6) Small deoxygenation (O/C ratio original > 1d > 5d) may result from loss of oxygen-rich thermodynamic endmember molecules H_2O and CO_2 upon oxidative degradation of DOM; 7) Small increase of %CHNO and N/C ratio at 5d may reflect higher longevity of CHNO molecules.

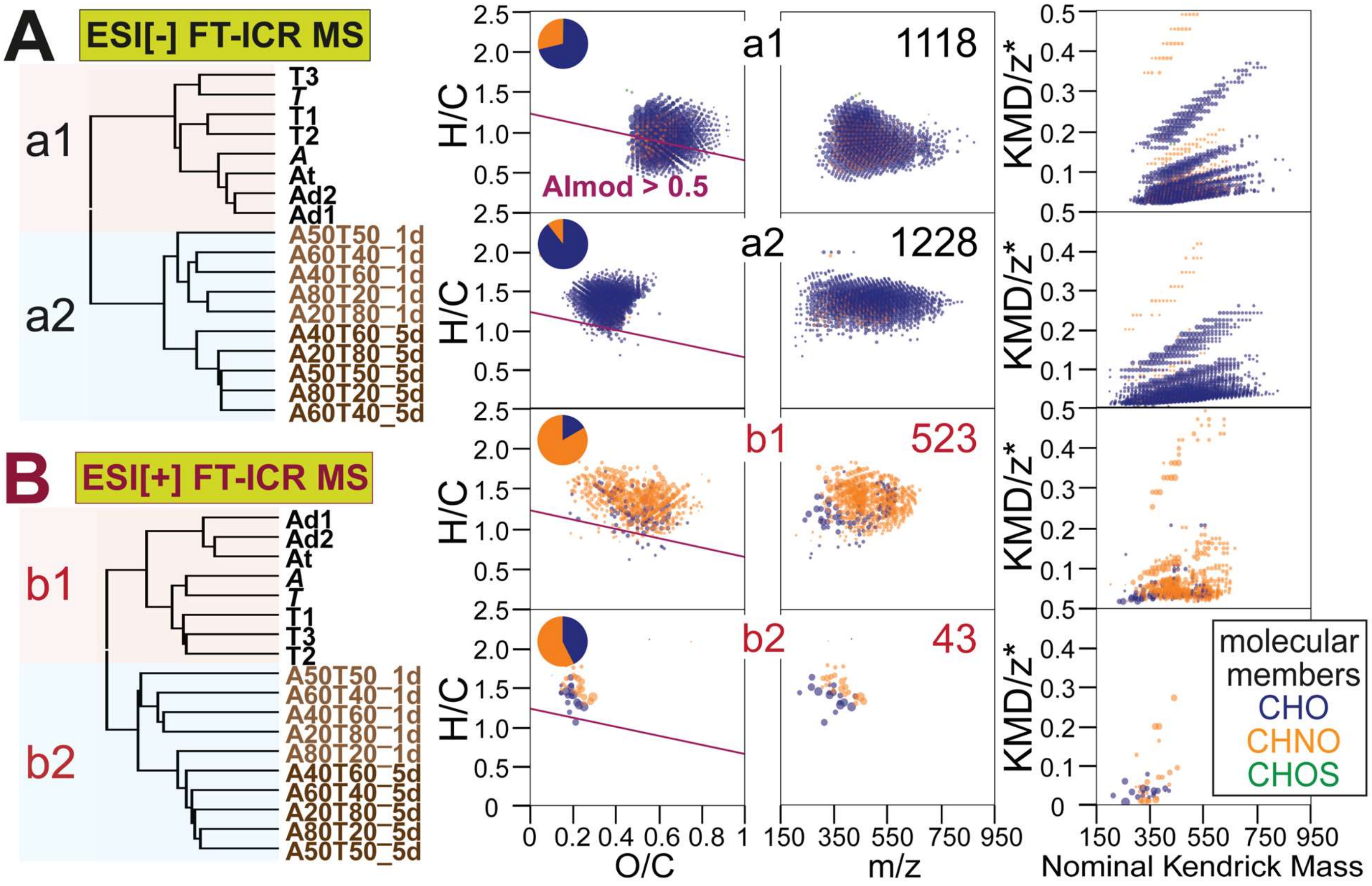

Figure S10. HCA of assigned SPE-DOM molecular formulae in the unmixed and mixed A+T water pre- and post-incubation. Van Krevelen, mass-edited H/C, and KMD/z* diagrams[16] show CHO (blue), CHNO (orange), and CHOS (green) molecular classes that were relatively more abundant in clusters a1/a2, b1/b2, respectively. The pie plots depict percentages of the counts of different molecular classes. The numbers show counts of compounds. The molecular formulae positioned below the purple line in the van Krevelen diagrams have modified aromaticity index (AI_{mod})[15] higher than 0.5.

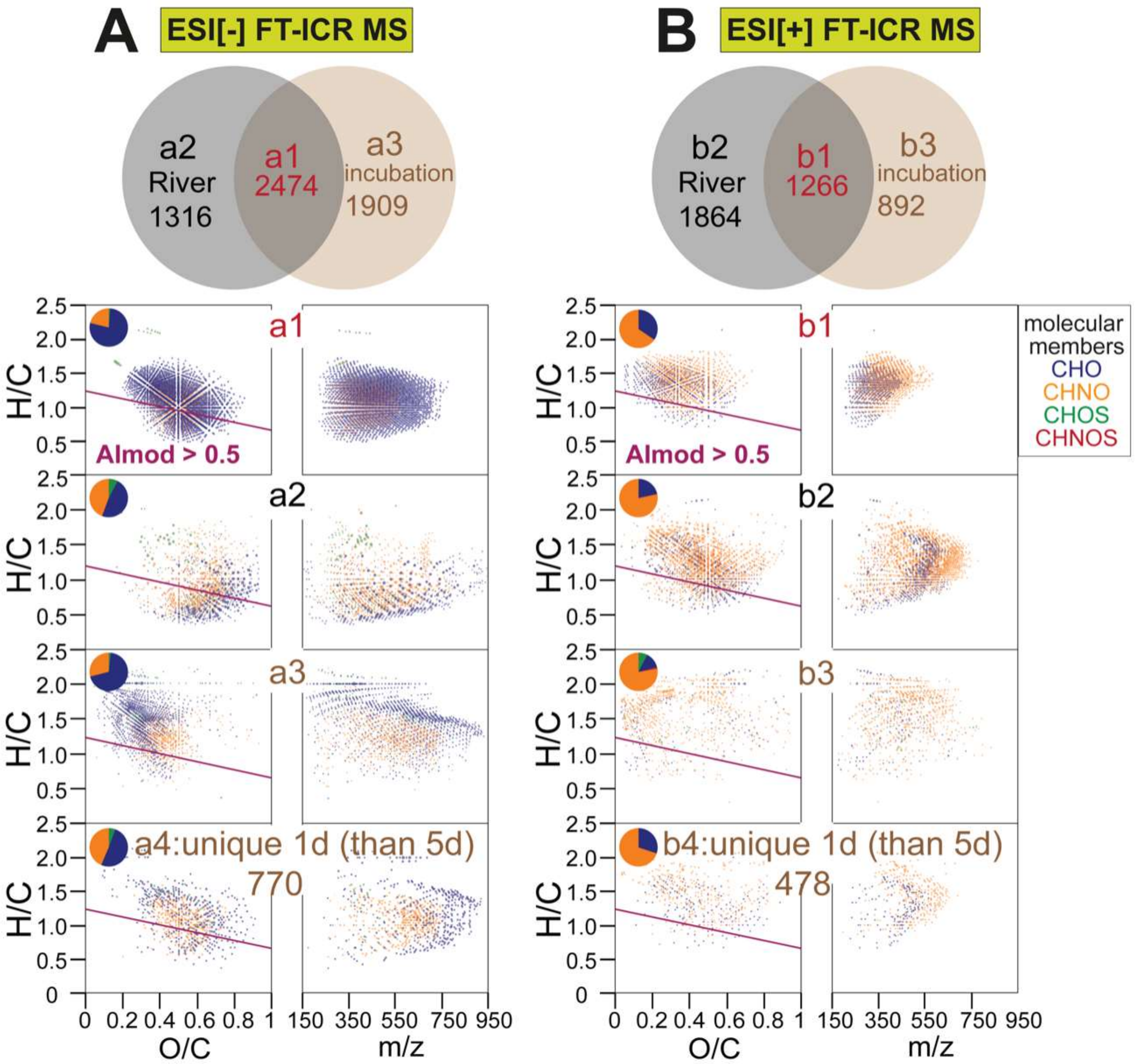

Figure S11. Common and unique molecular signatures in unmixed and mixed A+T water pre- and post-incubation. The numbers show counts of compounds. Van Krevelen and mass-edited H/C diagrams show CHO (blue), CHNO (orange), CHOS (green), and CHNOS (red) *m/z* ions that were shared before and after incubation (a1 and b1); unique in original water but were not in incubated water (a2 and b2); unique in incubated water but were not in original water (a3 and b3); unique in one-day incubated water (1d) but were not present in five-day incubated water (5d) (a4 and b4). There was no compound unique to 5d and not present 1d. The pie plots depict percentages of the counts of different molecular classes. The numbers show counts of compounds. The molecular formulae positioned below the purple line in the van Krevelen diagrams have modified aromaticity index (AI_{mod})[15] higher than 0.5.

Footnote: (a1, b1) ESI[-] MS denotes more CHO than CHNO compounds of near average H/C and O/C ratio for which number of isomers for given molecular composition in maximal island of stability[17], chemodiversity of observed CHO molecules is larger than that of CHNO compounds as shown by coverage in van Krevelen and mass-edited H/C diagrams. ESI[+] MS denotes more CHNO than CHO compounds and displacement of CHNO molecules toward higher H/C ratio and higher mass. (a2, b2) ESI[-] MS demotes loss or certain hydrogen-deficient polyphenols, and lower mass CHOS compounds from original waters; ESI[+] MS denotes a surprising large-scale loss of CHNO molecules at near average H/C and O/C ratios. (a3, b3) Synthesis of several long-range series of CHO molecules near H/C ratio ~2, and a higher mass (*m/z* ~ 500-950) group of less oxygenated (O/C ratio < 0.45) molecules.

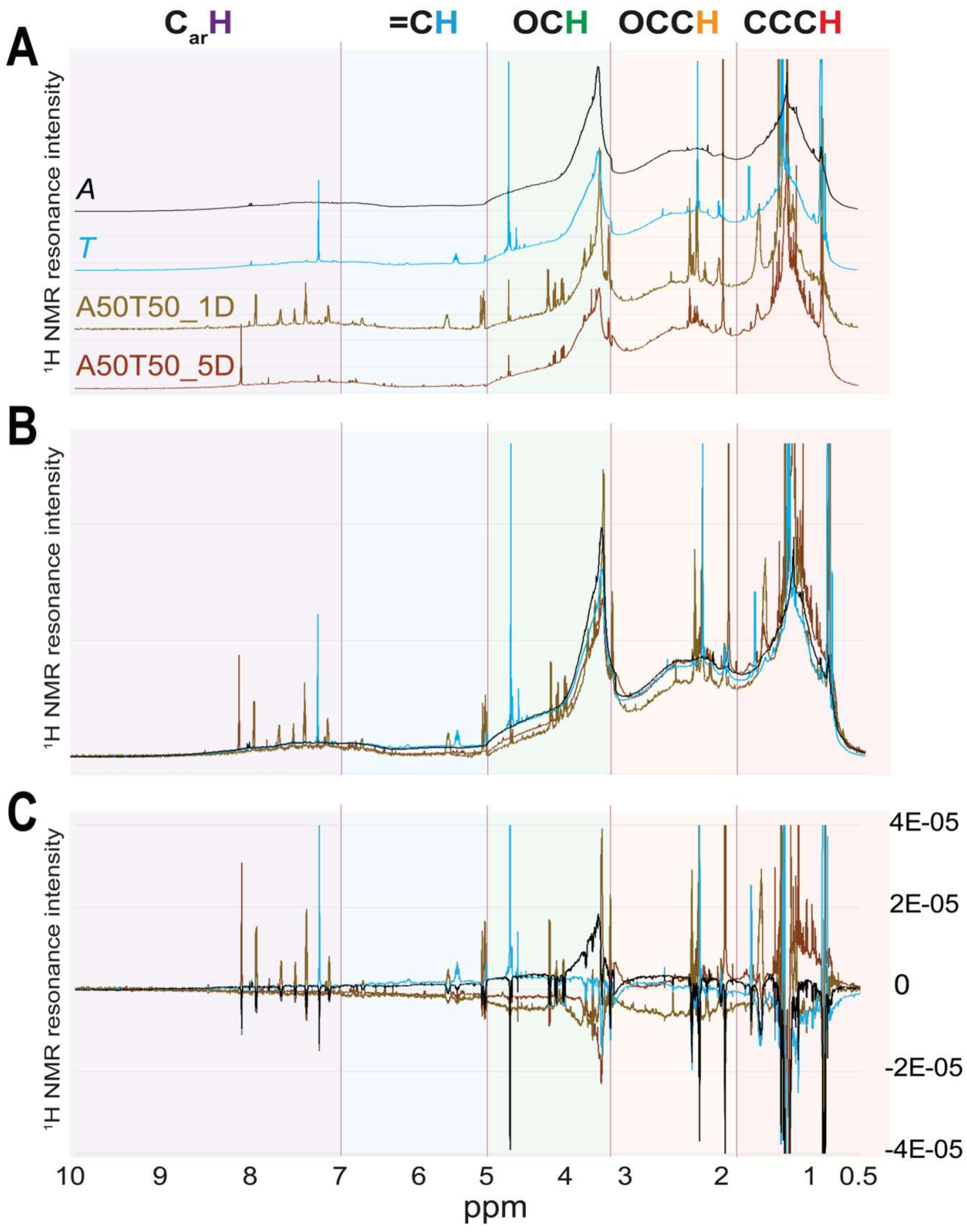

Figure S12. ^{1}H NMR spectra of SPE-DOM in unmixed and mixed A+T water pre- and post- incubation. The ^{1}H NMR spectra are exemplified with sample *A*, *T*, A50T50_1d, and A50T50_5d. ^{1}H NMR spectra are entire region normalized (800 MHz, CD_3OD; δ_H = 0.5-10.0 ppm with exclusion of residual water and methanol NMR resonances). The ^{1}H NMR spectra referred to average abundance in the four DOM are edited by each normalized spectra subtract the average spectra of the four DOM (C).

Table S9. ^{1}H NMR section integrals (percent of non-exchangeable protons, 800 MHz; CD_3OD, exclusion of residual water, and methanol)) and key substructures of SPE-DOM in unmixed and mixed A+T water pre- and post- incubation. The fundamental substructures include aromatics C_{ar}**H**, $\delta_H \sim 7.0\text{-}10.0$ ppm; olefins $\delta_H \sim 5.3\text{-}7.0$ ppm; oxygenated aliphatic units (OC**H**) and "carbohydrate-like" and methoxy OC**H**$_3$ units $\delta_H \sim 3.2\text{-}4.9$ ppm; branched aliphatic units (C**H**$_2$)$_n$, "acetate-analogue" and CRAM $\delta_H \sim 1.9\text{-}3.2$ ppm; functionalized aliphatics $\delta_H \sim 1.35\text{-}1.9$ ppm; polyethylene group $\delta_H \sim 1.25\text{-}1.35$ ppm, OCC**H** units; pure aliphatics $\delta_H \sim 0.5\text{-}1.25$ ppm, CCC**H** units.

δ (^{1}H) [ppm]	10.0-7.0	7.0_5.3	4.9-3.2	3.2-1.9	1.9-1.35	1.35-1.25	1.25-0.5	1.9-0.5
key substructures	C_{ar}**H** (%)	C=C**H**, O_2C**H** (%)<br>O_2C**H** (%)	OC**H** (%)	OCCH (%)	OCCC**H** (%)	(C**H**$_2$)$_n$ (%)	CCCH$_3$ (%)	total aliphatic section (%)
Ad1	4.8	3.4	31.0	26.6	14.8	4.7	14.7	34.2
Ad2	4.9	5.1	32.8	26.5	14.6	4.3	11.9	30.7
A	*4.8*	*4.6*	*30.0*	*27.4*	*15.1*	*4.6*	*13.4*	*33.1*
At	4.9	3.8	32.5	25.8	14.5	4.6	13.8	33.0
T1R	4.9	5.2	32.8	26.3	14.2	4.7	11.9	30.8
T2R	4.9	4.0	29.7	26.5	14.8	5.1	14.8	34.8
T3R	4.8	4.7	28.9	28.1	15.2	5.0	13.3	33.5
T	*4.8*	*6.7*	*27.3*	*26.0*	*14.2*	*5.4*	*15.5*	*35.1*
A20T80_1d	3.4	1.2	24.5	27.9	15.5	12.6	14.9	43.0
A40T60_1d	4.2	2.1	23.6	28.2	15.9	9.3	16.8	42.0
A50T50_1d	4.3	2.1	20.0	25.0	16.2	17.8	14.7	48.6
A60T40_1d	4.3	2.0	24.8	29.3	16.1	8.0	15.4	39.5
A80T20_1d	4.4	3.1	25.0	28.9	15.9	8.8	13.9	38.6
A20T80_5d	3.5	1.7	21.6	30.9	17.1	8.3	16.8	42.2
A40T60_5d	3.6	1.6	21.7	30.3	16.7	6.8	19.2	42.7
A50T50_5d	3.7	2.0	19.9	31.9	16.8	8.3	17.4	42.5
A60T40_5d	4.1	2.2	22.1	29.7	17.0	7.5	17.4	41.8
A80T20_5d	3.5	1.4	22.3	29.3	16.9	8.1	18.5	43.5

Footnote: 1) C_{ar}**H** decrease original > 1d > 5d; 2) Olefins strongly decline original > 1d, 5d; 3) OC**H** units decline original > 1d > 5d; 4) Remotely functionalized aliphatic units and carboxylic acids increase original < 1d < 5d; 5) (CH_2)$_n$ strongly increases from original to 1d; then somewhat declines; 6) Continual increase of purely aliphatic units including alkyl; 7) strong increase original < 1d < 5d.

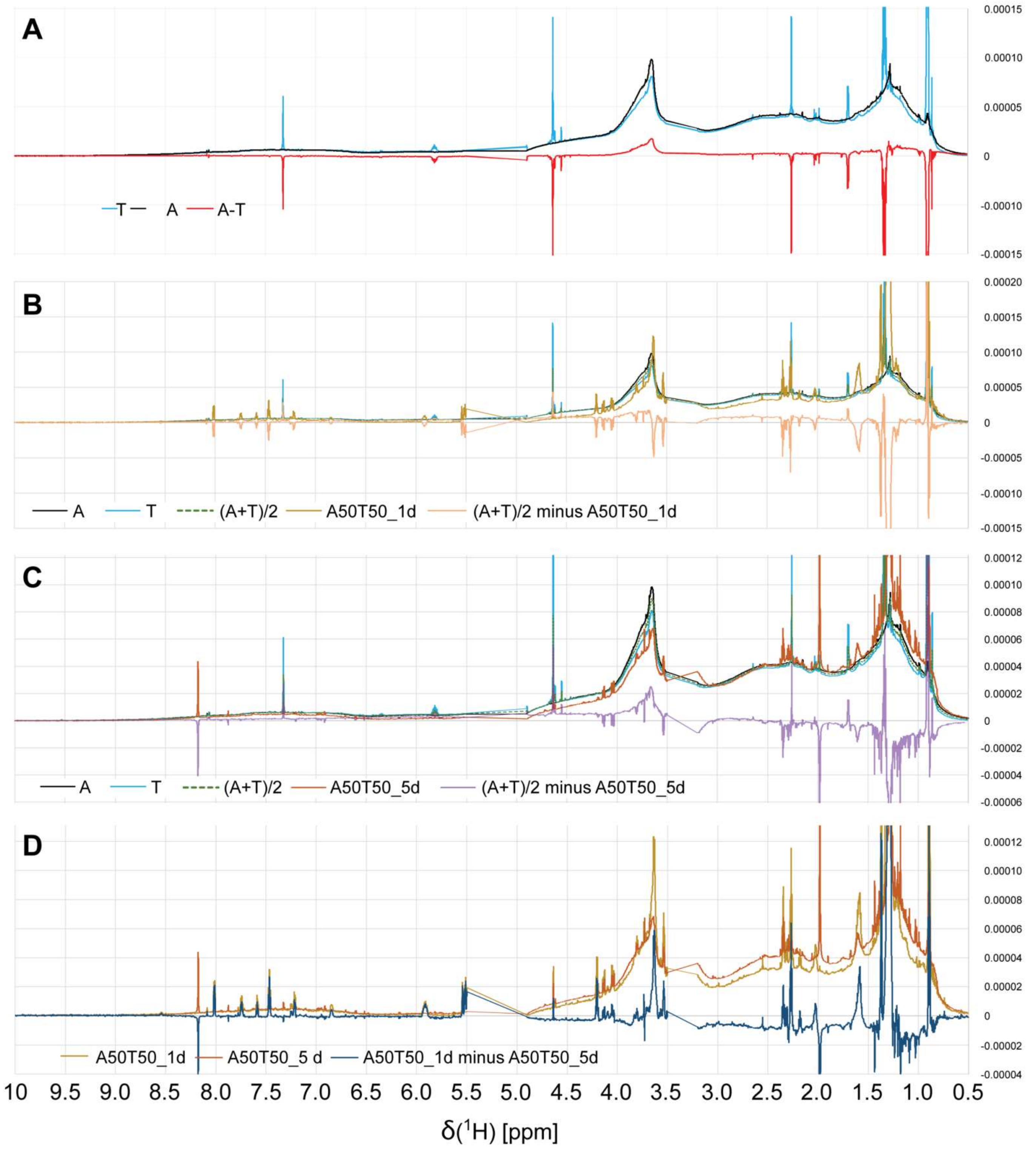

Figure S13. Difference ^{1}H NMR spectra (800 MHz, CD_3OD) of Amazon and Tapajós river SPE-DOM according to status of incubation (full vertical expansion cf. Figure S14). (A) Amazon minus Tapajós river, with Amazon (A), Tapajós (T), and Amazon minus Tapajós (A-T). (B) difference ^{1}H NMR spectrum after one day of incubation, with A, T, averaged Amazon and Tapajós ((A+T)/2), A50T50_1d, and (A+T)/2 minus A50T50_1d. (C) difference ^{1}H NMR spectrum after five days of incubation, with A, T, (A+T)/2, A50T50_5d, and (A+T)/2 minus A50T50_5d. (D) difference ^{1}H NMR spectrum one day minus five days of incubation, with A50T50_1d, A50T50_5d, and (A50T50_1d minus A50T50_5d).

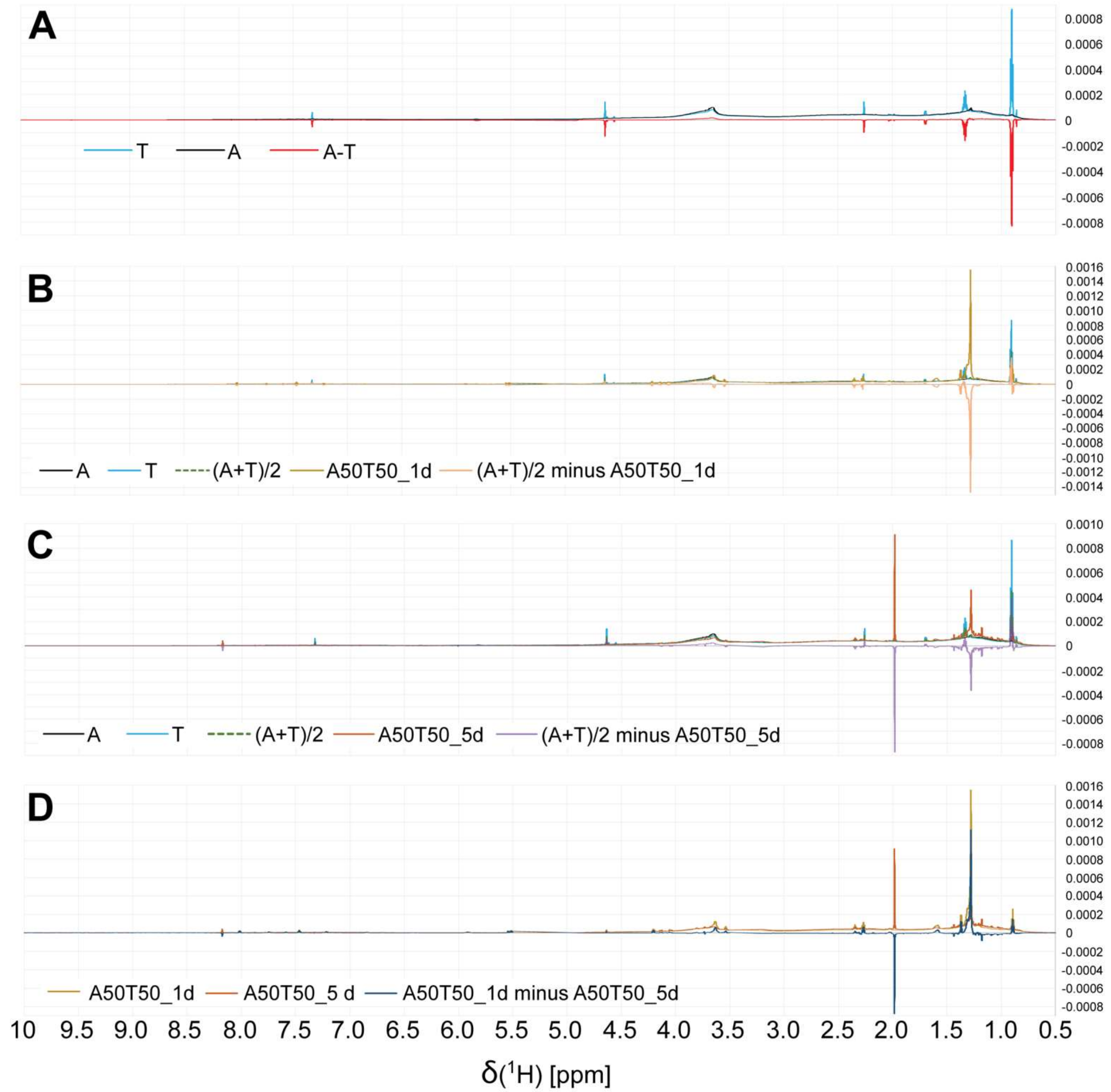

Figure S14. Difference ^{1}H NMR spectra (800 MHz, CD_3OD) of Amazon and Tapajós river SPE-DOM according to status of incubation. (A) Amazon minus Tapajós river, with Amazon (A), Tapajós (T), and Amazon minus Tapajós (A-T). (B) difference ^{1}H NMR spectrum after one day of incubation, with A, T, averaged Amazon and Tapajós ((A+T)/2), A50T50_1d, and (A+T)/2 minus A50T50_1d. (C) difference ^{1}H NMR spectrum after five days of incubation, with A, T, (A+T)/2, A50T50_5d, and (A+T)/2 minus A50T50_5d. (D) difference ^{1}H NMR spectrum one day minus five days of incubation, with A50T50_1d, A50T50_5d, and (A50T50_1d minus A50T50_5d).

Table S10. HBP and DCF in unmixed and mixed S+N and A+T waters pre- and post- incubation. SD, Standard Deviation; n, number of replicates; N.A., not available.

Sample	HBP (mean) μgC $L^{-1}h^{-1}$	HBP (SD) μgC $L^{-1}h^{-1}$	HBP (n)	DCF (mean) μgC $L^{-1}h^{-1}$	DCF (SD) μgC $L^{-1}h^{-1}$	DCF (n)
S	0.0317	0.0049	5	0.7594	0.0929	5
N	0.0284	0.0028	5	1.6634	0.6510	5
S+N mixing zone	0.0274	0.0042	6	6.0521	0.7198	5
S50N50_1h	0.0261	0.0027	5	N.A.	N.A.	N.A.
A1	3.5606	0.7900	5	13.2677	1.5780	5
A2	17.2304	2.3757	5	0.6946	0.8074	5
A	0.1535	0.0212	4	0.4730	0.5498	3
T	0.1492	0.0075	4	0.4395	0.2414	3
A+T mixing zone	0.1535	0.0065	4	1.7905	1.1563	3

Table S11. Ranges of heterotrophic bacterial production (HBP) and dark carbon fixation (DCF) in this study and in the Amazon River other aquatic systems.

Ecosystem	HBP (µgC $L^{-1}h^{-1}$)	HBP (Ref.)	DCF (µgC $L^{-1}h^{-1}$)	DCF (Ref.)
Amazon river	<0.001 to 27.2	This Study	<0.001 to 16.0	This Study
Amazon river	0.1 to 7.0	Benner et al., 1995a[18]; Farjalla, 2014[11]; Farjalla et al., 2002[19]; Vidal et al., 2015[20]; Wissmar et al., 1981[21]	NA	
Rodrigo de Freitas Lagoon (Brazil)	0.7 to 47.5	Signori et al., 2020[22]	0.6 to 4.3	Signori et al., 2020[22]
Guanabara Bay (Brazil)	2.20 to 122.61	Signori et al., 2018[23]	0.001 to 2.5	Signori et al., 2018[23]
Boreal lakes (Sweden)	<0.001 to 1.1	Bastviken et al. 2003[24]	<0.001 to 0.3	Bastviken et al. 2003[24]
Scheldt estuary (Belgium)	0.2 to 25	Goosen et al., 1997[25]	0.001 to 0.2	Andersson et al., 2006[30]
Ebro River estuary (Spain)	0.7 to 5.6	Calderón-Paz et al., 1993[26]	0.04 to 0.7	Casamayor et al., 2001[31]
Columbia River estuary (USA)	0.13 to 4.5	Crump et al., 1998[27]	<0.001 to 2	Brauer et al., 2013[32]
Karstic Lakes Spain	<0.001 to 8.75	Overman et al., 1996[28]	0.05 to 41	Casamayor et al., 2001[31]; Noguerola et al. 2015[33]
Lake Cadagno	0.002 to 0.006	Saini et al., 2022[29]	1.9 to 4.4	Di Nezio et al., 2021[34]

References

1 McClain, M. E. & Naiman, R. J. Andean Influences on the Biogeochemistry and Ecology of the Amazon River. *BioScience* **2008***, 58,* 325-338.

2 Aucour, A. M. et al. The Amazon River: behaviour of metals (Fe, Al, Mn) and dissolved organic matter in the initial mixing at the Rio Negro/Solimões confluence. *Chem. Geol.* **2003***, 197,* 271-285.

3 Richey, J. E. et al. Biogeochemistry of carbon in the Amazon River. *Limnol. Oceanogr.* **1990***, 35,* 352-371.

4 Dvorski, S. E. et al. Geochemistry of Dissolved Organic Matter in a Spatially Highly Resolved Groundwater Petroleum Hydrocarbon Plume Cross-Section. *Environ. Sci. Technol.* **2016***, 50,* 5536-5546.

5 Schmitt-Kopplin, P. et al. Dissolved organic matter in sea spray: a transfer study from marine surface water to aerosols. *Biogeosciences* **2012***, 9,* 1571-1582.

6 Schmitt-Kopplin, P. et al. Analysis of the unresolved organic fraction in atmospheric aerosols with ultrahigh-resolution mass spectrometry and nuclear magnetic resonance spectroscopy: organosulfates as photochemical smog constituents. *Anal. Chem.* **2010***, 82,* 8017-8026.

7 Hertkorn, N. et al. High-field NMR spectroscopy and FTICR mass spectrometry: powerful discovery tools for the molecular level characterization of marine dissolved organic matter. *Biogeosciences* **2013***, 10,* 1583-1624.

8 Valderrama, J. C. The simultaneous analysis of total nitrogen and total phosphorus in natural waters. *Mar. Chem.* **1981***, 10,* 109-122.

9 Gonsior, M. et al. The chemodiversity of algal dissolved organic matter from lysed Microcystis aeruginosa cells and its ability to form disinfection by-products during chlorination. *Water Res.* **2019***, 155,* 300-309.

10 Kirchman, D., K'nees, E. & Hodson, R. Leucine incorporation and its potential as a measure of protein synthesis by bacteria in natural aquatic systems. *Appl. Environ. Microbiol.* **1985***, 49,* 599-607.

11 Farjalla, V. F. Are the mixing zones between aquatic ecosystems hot spots of bacterial production in the Amazon River system? *Hydrobiologia* **2014***, 728,* 153-165.

12 Simon, M. & Azam, F. Protein content and protein synthesis rates of planktonic marine bacteria. *Mar. Ecol. Prog. Ser.* **1989***, 51,* 201-213.

13 Santoro, A. L. et al. Dark carbon fixation: an important process in lake sediments. *PLoS One* **2013***, 8,* e65813.

14 Åberg, J. & Wallin, M. Evaluating a fast headspace method for measuring DIC and subsequent calculation of pCO2 in freshwater systems. *Inland Waters* **2014***, 4,* 157-166.

15 Koch, B. P. & Dittmar, T. From mass to structure: an aromaticity index for high-resolution mass data of natural organic matter. *Rapid Commun. Mass Spectrom.* **2016***, 30,* 250-250.

16 Hsu, C. S., Qian, K. & Chen, Y. C. An innovative approach to data analysis in hydrocarbon characterization by on-line liquid chromatography-mass spectrometry. *Anal. Chim. Acta.* **1992***, 264,* 79-89.

17 Hertkorn, N. et al. High-precision frequency measurements: indispensable tools at the core of the molecular-level analysis of complex systems. *Anal. Bioanal. Chem.* **2007***, 389,* 1311-1327.

18 Benner, R., S. et al. Bacterial carbon metabolism in the Amazon River system. *Limnol. Oceanogr.* **1995***, 40,* 1262-1270.

19 Farjalla, V. F. et al. Nutrient limitation of bacterial production in clear water Amazonian ecosystems. *Hydrobiologia* **2002**, *489,* 197-205.
20 Vidal, L. O. et al. Hydrological pulse regulating the bacterial heterotrophic metabolism between Amazonian mainstems and floodplain lakes. *Frontiers in Microbiology* **2015**, *6,* 1054.
21 Wissmar, R. et al. Plankton metabolism and carbon processes in the Amazon River, its tributaries, and floodplain waters, Peru-Brazil, May-June 1977. *Ecology* **1981**, *62,* 1622-1633.
22 Signori, C. N. et al. Bacterial production prevails over photo-and chemosynthesis in a eutrophic tropical lagoon. *Estuarine, Coastal and Shelf Science* **2020**, *243,* 106889.
23 Signori, C. N. et al. Temporal variability of dark carbon fixation and bacterial production and their relation with environmental factors in a tropical estuarine system. *Estuaries and Coasts* **2018**, *41,* 1089-1101.
24 Bastviken, D. et al. Methane as a source of carbon and energy for lake pelagic food webs. *Ecology* **2003**, *84,* 969-981.
25 Goosen, N. K. et al. Regulation of annual variation in heterotrophic bacterial production in the Schelde estuary (SW Netherlands). *Aquatic Microbial Ecology* **1997**, *12,* 223-232.
26 Calderón-Paz, J. I. et al. Heterotrophic bacterial production in systems of the northern Spanish Mediterranean Region. *Internationale Vereinigung für theoretische und angewandte Limnologie: Verhandlungen* **1993**, *25,* 739-742.
27 Crump, B. C. et al. Dominance of particle-attached bacteria in the Columbia River estuary, USA. *Aquatic Microbial Ecology* **1998**, *14,* 7-18.
28 Overmann, J. et al. Purple sulfur bacteria control the growth of aerobic heterotrophic bacterioplankton in a meromictic salt lake. *Applied and Environmental Microbiology* **1996**, *62,* 3251-3258.
29 Saini, J. S. et al. Bacterial, Phytoplankton, and Viral Distributions and Their Biogeochemical Contexts in Meromictic Lake Cadagno Offer Insights into the Proterozoic Ocean Microbial Loop. *Mbio.* **2022**, *13,* e00052-22.
30 Andersson, M. G. et al. Comparison of nitrifier activity versus growth in the Scheldt estuary—a turbid, tidal estuary in northern Europe. *Aquatic Microbial Ecology* **2006**, *42,* 149-158.
31 Casamayor, E. O. et al. Primary production in estuarine oxic/anoxic interfaces: contribution of microbial dark CO_2 fixation in the Ebro River Salt Wedge Estuary. *Marine Ecology Progress Series* **2001**, *215,* 49-56.
32 Bräuer, S. L. et al. Dark carbon fixation in the Columbia River's estuarine turbidity maxima: molecular characterization of red-type cbbL genes and measurement of DIC uptake rates in response to added electron donors. *Estuaries and coasts* **2013**, *36,* 1073-1083.
33 Noguerola, I. et al. Heterotrophic bacterial production in systems of the northern Spanish Mediterranean Region. *FEMS microbiology ecology* **2015**, *91,* fiv086.
34 Di Nezio, F. et al. Anoxygenic photo-and chemo-synthesis of phototrophic sulfur bacteria from an alpine meromictic lake. *FEMS microbiology ecology* **2021**, *97,* fiab010.
